# Signaling pathways and targeted therapy for pulmonary hypertension

**DOI:** 10.1038/s41392-025-02287-8

**Published:** 2025-07-01

**Authors:** Joseph Adu-Amankwaah, Yue Shi, Hequn Song, Yixuan Ma, Jia Liu, Hao Wang, Jinxiang Yuan, Kun Sun, Qinghua Hu, Rubin Tan

**Affiliations:** 1 Department of Physiology, School of Basic Medical Sciences, Xuzhou Medical University, Xuzhou, Jiangsu China; 2 First Clinical Medical School, Xuzhou Medical University, Xuzhou, Jiangsu China; 3 Second Clinical Medical School, Xuzhou Medical University, Xuzhou, Jiangsu China; 4 Lin He’s Academician Workstation of New Medicine and Clinical Translation, Jining Medical University, Jining, Shandong China; 5 Institute for Developmental and Regenerative Cardiovascular Medicine, Xinhua Hospital, School of Medicine, Shanghai Jiao Tong University, Shanghai, China; 6 Department of Pathophysiology, School of Basic Medicine, Tongji Medical College, Huazhong University of Science and Technology (HUST), Wuhan, China; 7 Key Laboratory of Pulmonary Diseases of Ministry of Health, Tongji Medical College, HUST, Wuhan, China

**Keywords:** Cardiovascular diseases, Preclinical research, Inflammation, Cell biology

## Abstract

Pulmonary hypertension (PH) is a global health issue characterized by high mortality. The main targets for current therapies in PH focus on the prostacyclin, nitric oxide, and endothelin pathways. While the approaches targeting these pathways form the foundation of standard PH treatment, the challenge remains to develop more effective therapeutic strategies. Evidence of pathological characteristics in PH illustrates other cell signaling pathways that also participate in the proliferation, apoptosis, extracellular matrix remodeling, mitochondrial dysfunction, inflammation, endothelial-to-mesenchymal transition, ferroptosis, pyroptosis, and the intricate network of cell-cell interactions of endothelial cells, smooth muscle cells, fibroblasts, and macrophages. In this review, we explore the roles of twenty key signaling pathways in PH pathogenesis. Furthermore, the crosstalks among some pathways offer a more detailed understanding of the complex mechanisms of PH. Considering the crucial role of signaling pathways in PH progression, targeting these aberrant signaling or their hub molecules offers great potential for mitigating PH pathology. This review delves into a variety of therapeutic approaches for PH that target critical signaling pathways and network interactions, including gene therapy, cell therapy, and pharmacological interventions. Supported by evidence from both animal studies and clinical trials, these strategies aim to reverse pathological alterations in pulmonary vessels and restore their normal function, addressing the significant health challenges associated with PH.

## Introduction

Pulmonary hypertension (PH) is a complex and diverse condition characterized by an elevated mean pulmonary artery pressure (mPAP) exceeding 20 mmHg at rest.^1,2^ First identified in 1891 by Dr. Ernst von Romberg, PH remained understudied until the 1960s when appetite suppressant cases sparked interest;^3^ however, its mechanisms remain unclear. Epidemiologically, PH presents a complex landscape, with significant variability in prevalence and survival across different regions and patient populations, with incidence ranging from 15 to 48 per million people globally, at least 1% of the global population.^4^

According to the 2022 ESC/ERS Guidelines, PH is classified into five distinct groups to capture its diverse etiologies and clinical manifestations.^2,4^ Pulmonary arterial hypertension (PAH, Group 1, rare), which encompasses idiopathic (IPAH), heritable (HPAH), drug-induced, HIV; PH associated with left-sided heart disease (Group 2, very common); PH associated with lung disease (Group 3, common), such as hypoxia-PH and chronic obstructive pulmonary disease (COPD)-PH; PH associated with pulmonary artery obstructions (Group 4, rare), such as chronic thromboembolic PH (CTEPH); PH with unclear and/or multifactorial mechanisms, including metabolic (Group 5, rare).^2,4^ The varying proportions of these groups across different populations highlight the diverse underlying causes of PH and underscore disparities in healthcare access and management strategies.^2,4^ Current therapies for PH primarily focus on pharmacological agents that target the PGI, nitric oxide (NO), and ET-1 pathways.^4^ However, these treatments, predominantly developed for PAH, provide symptomatic relief for only a subset of patients, and the overall mortality rate remains unacceptably high. Additionally, there is a significant paucity of drug development targeting other groups, underscoring a critical need for more diverse therapeutic options in the management of PH.

**Fig. 1:**
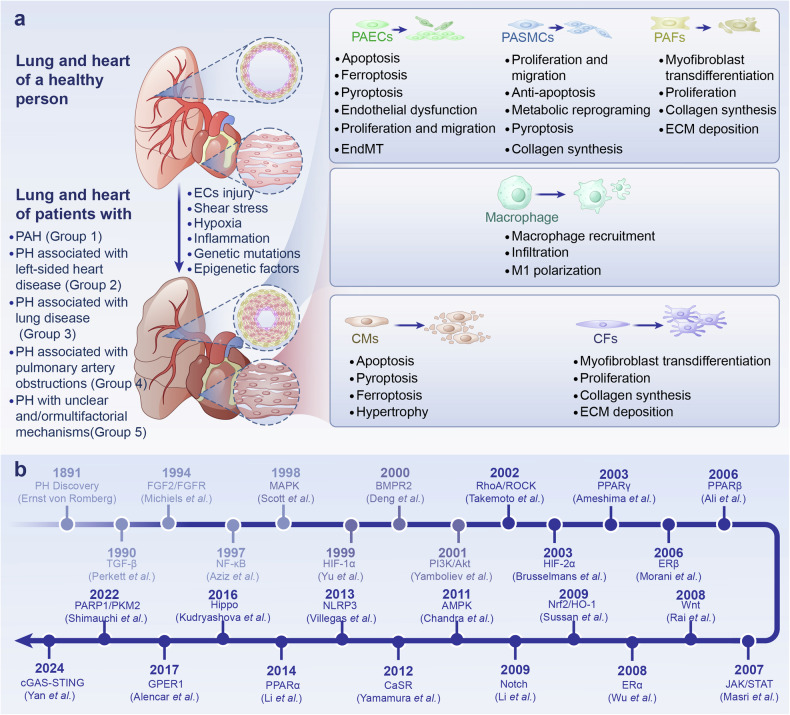
Classification, Cellular Processes and Key Signaling Milestones in PH. **a** This panel illustrates the World Health Organization (WHO) classification of pulmonary hypertension, highlighting how the lungs and heart of a healthy individual can undergo structural remodeling in response to various factors, including endothelial cell injury, shear stress, hypoxia, inflammation, genetic mutations, and epigenetic influences. The figure further depicts key cellular processes involved in the remodeling of the pulmonary artery and heart, focusing on the roles of different cell types: PAECs, PASMCs, PAFs, CMs, CFs, and macrophages. These processes include apoptosis, necrosis, ferroptosis, pyroptosis, EndMT, proliferation, migration, metabolic reprogramming, polarization, and transdifferentiation, which contribute to the pathogenesis of pulmonary hypertension. **b** This timeline of significant milestones in PH research, spanning from 1891, when the condition was first discovered, to 2024, when key signaling pathways associated with PH were identified. *PAECs* pulmonary artery endothelial cells, *PASMCs* pulmonary artery smooth muscle cells, *PAFs* pulmonary artery fibroblasts, *CMs* cardiomyocytes, *CFs* cardiac fibroblasts, *EndMT* endothelial-to-mesenchymal transition, *PH* pulmonary hypertension, *PAH* pulmonary artery hypertension

Central to the pathophysiology of PH is the dysregulation of cellular interactions in the pulmonary vasculature especially pulmonary arterioles, where pulmonary artery endothelial cells (PAECs), pulmonary artery smooth muscle cells (PASMCs), pulmonary artery fibroblasts (PAFs), immune cells, and the extracellular matrix (ECM) form a dynamic microenvironment. The disease begins with endothelial cell (EC) apoptosis, which triggers a cascade of pathological responses, including endothelial-to-mesenchymal transition (EndMT),^5^ the proliferation and migration of PASMCs and PAFs, leading to ECM remodeling and fibrosis to stiffen vascular and increased pulmonary vascular resistance (PVR). Over time, this hyper-proliferative state becomes resistant to apoptosis, resulting in occlusive neointimal lesions that severely restrict blood flow.^6,7^ Additionally, the recruitment of inflammatory cells, particularly macrophages, plays a critical role in the pathological remodeling process by secreting growth factors and cytokines that enhance the proliferation of PASMCs and PAFs.^8^ Moreover, mitochondrial dysfunction and metabolic reprogramming in PASMCs shift the cell phenotype toward hyper-proliferation and resistance to apoptosis, further driving the narrowing of the pulmonary arteries (Fig. 1a).^9,10^

So, a deeper understanding of the signaling pathways governing cell-cell interactions is crucial for developing targeted therapies for PH. Several vital signaling pathways play pivotal roles in regulating vascular remodeling via inflammation, metabolic reprogramming, and various forms of cell death, including apoptosis, ferroptosis, pyroptosis, and fibrosis. Each of these pathways influences critical processes in various cells. For instance, BMPR2 signaling impacts PASMC proliferation and ECM deposition,^11^ while transforming growth factor TGF-β and Wnt pathways are central to fibrosis and ECM remodeling.^12^ HIF, NF-κB, and NLRP3 pathways regulate the inflammatory response and metabolic changes in the pulmonary vasculature.^13,14^ Additionally, PI3K/Akt is involved in the apoptosis of PAECs, while AMPK and MAPK pathways contribute to metabolic adaptation and stress responses.^15^ Therefore, this review explores the roles of twenty key signaling pathways in PH: BMPR2, TGF-β, HIF, MAPK, PI3K/Akt, NF-κB, NLRP3, Notch, AMPK, Wnt, FGF2/FGFR, PPAR, RhoA/ROCK, Estrogen Receptors, JAK/STAT, CaSR, Hippo, Nrf2/HO-1, PARP1/PKM2, and cGAS-STING signaling pathways (Fig. 1b). Cross-talks among some pathways shape the dynamic interplay between different cell types, contributing to the progression of PH. Understanding how these pathways interact and contribute to the dysregulated pulmonary vascular environment offers promising avenues for developing targeted, multifaceted therapeutic strategies. By dissecting the molecular mechanisms underlying these interactions, we aim to identify potential therapeutic targets that could disrupt the pathological remodeling process and improve clinical outcomes for patients with PH.

## BMPR2 signaling pathway in PH

In 2000, the International primary pulmonary hypertension Consortium and Deng et al. identified mutations in the *BMPR2* gene as a cause of familial PH.^16,17^ By then, further investigations confirmed that mutations in *BMPR2* are responsible for familial PH, with ~26% of IPAH cases also linked to *BMPR2* mutations.^18^

BMPR2 is a critical receptor for transmitting signals from BMPs, which are cytokines belonging to the TGF-β superfamily. BMP signaling occurs through type I receptors (ALK1–7) and type II receptors (ActRIIA/ActRIIB; BMPR2; TGF-β receptor type 2).^19^ BMPR2 specifically binds to ligands such as BMP2 and BMP4, leading to the phosphorylation of BMPR1A/B and the subsequent activation of Smad1/5/8. These activated Smad proteins then translocate to the nucleus to regulate gene expression.^20^ Type I receptors exhibit a higher affinity for BMPs than type II receptors, and the specific distribution of these receptors influences how various cell types within tissues respond to BMPs.^21^ In PAECs, BMPR2 forms a complex with ALK1,^22^ while in PASMCs, it binds to ALK3.^23^ Proper expression and function of BMPR2 at different developmental stages are essential for the normal development of human cardiovascular and lung tissues.^24^

**Fig. 2:**
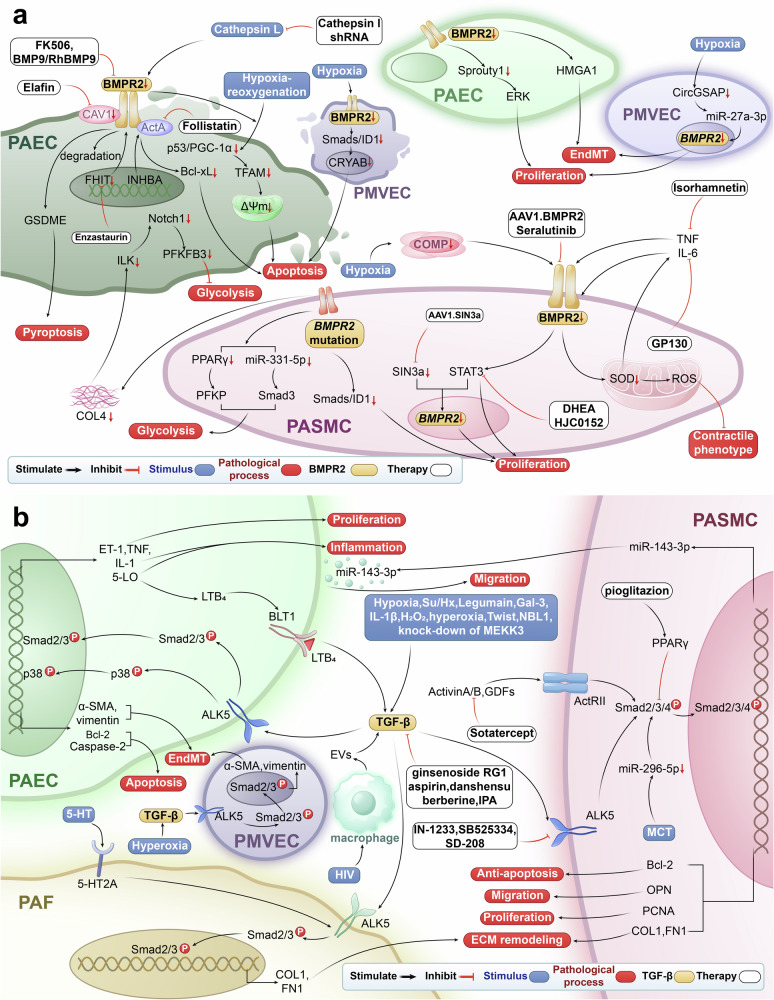
BMPR2 and TGF-β signaling pathways in PH. **a** BMPR2 signaling pathway and targeted therapy in PH. Cathepsin L, hypoxia, MCT, and direct *BMPR2* knockdown or mutations, inhibit BMPR2 signaling, which regulates GSDME, P53/PGC1-α, NOTCH, ERK, JNK, p38, PPARγ, miRNAs, STAT3, and SOD/ROS, leading to apoptosis or pyroptosis of PAECs and PMVECs in the early stage of PH, followed by hyperproliferation of PAECs, PMVECs and PASMCs. Additionally, EndMT, ECM remodeling (COL4 and COMP), contractile phenotype inhibition of PASMCs, and inflammation (IL-6, TNF, and HMGB1) are involved in PH progression. FK506, enzastaurin, follistatin, elafin, cathepsin l shRNA, seralutinib, AAV1.BMPR2, AAV1.SIN3a, isorhamnetin, GP130, DHEA, HJC0152, BMP9, RhBMP9 alleviate PH by upregulating or activating BMPR2 and its related pathways. **b** TGF-β signaling pathway and targeted therapy in PH. Su/Hx, galectin-3, Hypoxia, EVs derived from HIV-infected macrophages, IL-1β, H₂O₂, MCT and NBL1 upregulate TGF-β, which targets Smad2/3/4, or p38, decrease COL1, FN1, α-SMA, vimentin, OPN and PCNA, and increase Bcl-2, leading to ECM remodeling, proliferation, migration, anti-apoptotic, EndMT. Sotatercept, ginsenoside Rg1, aspirin, danshensu, berberine, IPA, pioglitazone, IN-1233, SB525334, and SD-208 mitigate PH by modulating TGF-β signaling. *PASMC* pulmonary artery smooth muscle cell, *PAEC* pulmonary artery endothelial cell, *PMVEC* pulmonary microvascular endothelial cell, *PAF* pulmonary artery fibroblast, *BMPR2* bone morphogenetic protein receptor type 2, *TGF-β* transforming growth factor-β, *FHIT* fragile histidine triad, *INHBA* inhibin-β-A, *ActA* activin-A, *GSDME* gasdermin E, *PGC-1α* peroxisome proliferator-activated receptor gamma coactivator1-alpha, *TFAM* transcription factor A mitochondrial, Δ*ψm* mitochondrial membrane potential, *ILK* integrin-linked kinase, *Smad* small mothers against decapentaplegic, *PFKFB3* 6-phosphofructo-2-kinase/fructose-2,6-biphosphatase 3, *ID1* inhibitor of differentiation 1, *CRYAB* a-crystallin B, *nmMLCK* non-muscle myosin light chain kinase, *HMGA1* high mobility group at-hook 1, *ERK* extracellular signal-regulated kinase, *JNK* c-Jun N terminal kinase, *MCT* monocrotaline, *NF-κB* nuclear factor kappa B, *CircGSAP* circular RNA-γ-secretase-activating protein, *PPARγ* peroxisome proliferator-activated receptorγ, *PFKP* phosphofructokinase 1 platelet isoform, *Drp1* dynamin-related protein 1, *HMGB1* high-mobility group box 1, *TLR4* Toll-like receptor-4, *COMP* cartilage oligomeric matrix protein, *SIN3a* switch-independent 3a, *DHEA* dehydroepiandrosterone, *HJC0152* a STAT3 inhibitor, *GP130* Glycoprotein 130, *Su/Hx* SU5416-hypoxia, *GDF* growth and differentiation factor, *FN1* fibronectin1, *OPN* osteopontin, *COL1* type I collagen, *ET-1* endothelin-1, *PCNA* proliferating cell nuclear antigen, *NBL1* neuroblastoma suppressor of tumorigenicity 1, *EV* extracellular vesicle, *TNF* tumor necrosis factor, *LTB*_4_ leukotrieneB_4_, *IPA* Inactivated Pseudomonas aeruginosa, *5-HT* 5-hydroxytryptamine, *5-LO* 5-lipoxygenase, *EndMT* endothelial-to-mesenchymaltransition, *Caspase* cysteinyl aspartate specific proteinase, *miR* microRNA, *COL4* collagen type IV

As of 2013, over 300 distinct *BMPR2* mutations have been identified, with frameshift and nonsense mutations being the most common.^25,26^ These mutations lead to BMPR2 protein truncation and nonsense-mediated messenger RNA (mRNA) decay, resulting in disrupted BMPR2 signaling.^26^ *BMPR2* mutations can occur across various functional domains, including the extracellular/ligand-binding, transmembrane, kinase, and cytoplasmic tail regions.^26^ These mutations exhibit complex polymorphisms, with some directly associated with PH.^26^ Recent studies have increasingly highlighted the role of BMPR2 signaling in various pathological processes in PH, including cell proliferation, apoptosis, metabolic and mitochondrial dysfunction, inflammation and immune regulation, ECM remodeling, EndMT, and cell pyroptosis (Fig. 2a).

### BMPR2 regulates apoptosis and cell proliferation in PH

BMPR2 abnormalities play a critical role in pulmonary vascular remodeling by regulating the transformation of PAECs from an early pro-apoptotic to an anti-apoptotic state and promoting the excessive proliferation of PASMCs, characterized by vascular muscularization and the formation of plexiform lesions.^27^

### Endothelial apoptosis

BMPR2 signal dysfunction predisposes pulmonary vascular endothelial cells to apoptosis during the early onset of PH. Teichert-Kuliszewska et al. revealed knockdown of *BMPR2* in PAECs increases apoptosis in response to injurious stress like serum deprivation.^28^ This might be associated with downregulation of the anti-apoptotic protein Bcl-xL.^29^ The over-expression of inhibin-β-A in PAECs enhances the generation of ActA, which interacts with BMPR2, facilitating its endocytosis and lysosomal degradation. This process exacerbates PAEC apoptosis, impairs angiogenesis, and contributes to hypoxia-PH in mouse models.^30^ Downregulation of *FHIT* and PTPN1, identified in the lungs or blood of PAH patients, was demonstrated to decrease BMPR2, inducing apoptosis of PAECs.^31,32^ Yang et al. noted a loss of ID1 expression in pulmonary arterial lesions of PAH patients with mutant *BMPR2*.^33^ In lung tissues of mice with hypoxia-PH, ID1 was downregulated due to impaired activation of BMPR2/ALK1, followed by diminished transcription of a-crystallin B, increasing apoptosis of pulmonary microvascular endothelial cells (PMVECs).^34^

### Anti-apoptosis and cell proliferation

With the progression of PH, impaired BMPR2 signaling makes pulmonary vascular endothelial cells manifest proliferative phenotype instead. Sun et al. reported that decreased CircGSAP allowed microRNA (miR)-27a-3p for enhanced inhibition of BMPR2, promoting hypoxia-induced hyper-proliferation and migration of PMVECs.^35^ The transition from pro-apoptotic to proliferative signaling in PAECs, driven by BMPR2 abnormalities, warrants further investigation to identify the factors influencing this regulatory shift.

Importantly, BMPR2 signaling abnormalities are involved in the excessive proliferation of PASMCs. In *Bmpr2*^*R899X*^ mutant mice, Nasim et al. observed that while the effect of Smad/ID1 signaling was diminished, TGF-β signaling was upregulated, activating TGF-β-activated kinase 1 and p38, inhibiting apoptosis and promoting proliferation of PASMCs.^36^ The mutations in the BMPR2 kinase domain (C347Y, C347R) inhibit BMP4-mediated phosphorylation of Smad1/5, reducing its nuclear localization and downregulating ID1 expression, thereby enhancing the proliferation of PASMCs.^37^ PDGF- and BMP4-stimulated activation of ERK1/2 mediated phosphorylation of Smad1 (at linker region Ser206), which was also responsible for the reduction of ID1 and proliferation of PASMCs, particularly in the presence of *Bmpr2* mutations like W9X.^37^ Collectively, *BMPR2* mutations diminish the anti-proliferative effects of the BMPR2/Smads/ID1 signaling pathway, resulting in excessive proliferation of PASMCs during PH. PGI analogs, such as iloprost and treprostinil, can counteract the hyper-proliferation of PASMCs by restoring the Smad/ID1 signaling disrupted by these mutations.^33^ The *R899X* nonsense mutation in the tail domain has been extensively studied to create models that closely mimic human PAH.^38–40^ West et al. established a mouse model with a smooth muscle-specific *R899X* mutation, which led to p38 phosphorylation, PASMC proliferation, vascular muscularization, and potentially stimulated EC hyperplasia through paracrine signaling, resulting in pulmonary vessel occlusion and increased mPAP.^38^ They also identified CD133^+^ cells within the smooth muscle layer, suggesting that some PASMCs may differentiate from infiltrating circulating cells, although the timing of this event in disease progression remains unclear.^38^

In addition to *BMPR2* mutation, downregulation of BMPR2 is of note for hyper-proliferation of PASMCs. BMPR2 repression resulted from SIN3a depletion, which increased BMPR2 promoter methylation and facilitated binding with CTCF, ultimately promoting human PASMC proliferation.^41^

### BMPR2 dysfunction promotes ECM remodeling in PH

ECM remodeling is involved in the pathophysiology of different groups of PH, with some ECM alterations being common and others unique. ECM changes in PH were discussed by Jandl et al. primarily in relation to PAH^42^ and were demonstrated in pulmonary hypertension due to left heart disease (PH-LHD),^43,44^ PH associated with chronic lung diseases.^45–47^ There is a correlation between BMPR2 abnormalities and the remodeling of specific ECM components. For instance, defects in BMPR2 signaling mediate endothelial damage and subsequent vascular remodeling by reducing type IV collagen production in PASMCs.^48^ Enhancing BMPR2 signaling through the inhibition of miR-17 has been shown to improve collagen deposition in blood vessels and heart.^11^ Cartilage oligomeric matrix protein also plays a role in the regulation of BMPR2 as an ECM protein. Yu et al. found that decreased cartilage oligomeric matrix protein in PASMCs inhibits BMP2/BMPR2 signaling.^49^ In bovine pulmonary arteries, hypoxia was shown to reduce cartilage oligomeric matrix protein expression in the ECM, decrease BMPR2 protein levels, downregulate SOD2 expression, increase reactive oxygen species (ROS), and inhibit the contractile phenotype of PASMCs.^50^

### BMPR2 dysfunction causes impaired mitochondrial metabolism and other abnormalities in PH

*Bmpr2* mutations have been demonstrated to sufficiently cause metabolic dysfunction, which is involved in the development of PAH.^51^ Abnormal BMPR2 function regulates the expression of various metabolism-related genes through alterations in downstream signaling pathways. Actually, under normal BMPR2 function in human PASMCs, BMP2 stimulation activated PPARγ, which inhibited Smad3 phosphorylation and nuclear translocation through direct interaction, while elevating miR-331-5p to downregulate PFKP, ultimately suppressing glycolysis and TGF-β-enhanced proliferation.^52^

In addition to mitochondrial metabolic alterations, *BMPR2* defects can induce other mitochondrial abnormalities. In mouse PMVECs, *Bmpr2* defects increase 3-(2-deoxy-β-d-erythro-pentofuranosyl)pyrimido[1,2-α]purin-10(3H)-one, causing mitochondrial damage as one of ROS-mediated mitochondrial DNA adducts.^53^ Mitochondria-mediated apoptosis and altered kinetics are also implicated in PAH development. In lung PAECs from *Bmpr2* knockout mice and human PAECs with *BMPR2* knockdown, hypoxia-reoxygenation conditions downregulate p53 transcriptional activity, and reduce PGC1α protein levels, leading to diminished activity of Nrf2, decreased transcription factor A mitochondrial, and reduced mitochondrial membrane potential (Δψm), ultimately contributing to Caspase-3/7-dependent apoptosis in PAECs.^54^ This cascade results in a decrease in the number of distal pulmonary arteries and an elevation of right ventricular (RV) systolic pressure (RVSP) in mice.^54^

### BMPR2 dysfunction promotes inflammation in PH

The interplay between inflammatory immune mechanisms and the BMPR2 signaling pathway has garnered increasing attention due to its significant role in PH. In models of *Bmpr2* knockdown and *Bmpr2*^*R899X*^ mutant mice, IL-15/IL-15Rα signaling is inhibited, resulting in a deficiency of natural killer cells. The defect in IL-15 signaling exacerbates PAH and contributes to RV remodeling, including capillary reduction and hypertrophy in hypoxia-PH and MCT-induced PAH (MCT-PAH) rats.^55^ The role of IL-6/STAT3 signaling in promoting PAH through stimulation of PASMC proliferation has been established in patients, animal models, and cellular studies. Specifically, in PASMCs from PAH patients with *BMPR2* mutations and *Bmpr2*^*+/−*^ mice, chronic BMPR2 deficiency inhibits the expression of SOD3, increases ROS production, enhances lipopolysaccharide-induced IL-6 expression and secretion, and promotes proliferation via activated STAT3.^56^ Elevated IL-6 levels in rat lung tissues with MCT-PAH have been shown to downregulate BMPR2 and activate STAT3, creating a vicious cycle that further stimulates the proliferation of PASMCs.^57^

### BMPR2 dysfunction promotes EndMT in PH

Piera-Velazquez and Jimenez define EndMT as a complex process wherein ECs lose their characteristic phenotype and adopt a mesenchymal phenotype.^58^ This transition is marked by the loss of cell-cell connections, alterations in cell morphology, increased motility, invasion, and contractility, with TGF-β identified as the primary inducer of EndMT.^58^ Ranchoux et al. demonstrated that the transcription factor Twist-1, alongside mesenchymal markers such as α-SMA and vimentin is upregulated in the lung tissue of *Bmpr2* mutant rats exhibiting pulmonary artery muscularization. This finding provides direct evidence linking BMPR2 signaling defects to EndMT and pulmonary vascular pathology.^7^ Downregulation of BMPR2 in PAECs induces EndMT by upregulating HMGA1 or activating AKT due to DLL4/Notch1 signaling impairment, leading to a spindle-like cell morphology with increased levels of markers such as α-SMA, SM22α, calponin, phospho-vimentin, and Slug.^59^

Similar to EndMT, pericytes are stimulated by CXCL12/CXCR4/CXCR-7, FGF2, and IL-6, enhancing their proliferation and migration. Subsequently, TGF-β signaling induces their transformation into smooth muscle-like cells, facilitating pulmonary vascular remodeling.^60,61^ Although direct regulation of pericytes by BMPR2 has not been reported, its association with inflammation and vascular remodeling warrants further investigation.

### BMPR2 dysfunction promotes pyroptosis in PH

Pyroptosis is a form of programmed cell death characterized by a strong inflammatory response. Upon exposure to pathogens or damaging stimuli, inflammasomes assemble within the cell, activating Caspase-1 through the classical pathway or Caspase-4/5/11 through the non-classical pathway, resulting in the cleavage of GSDMD. This process leads to cell membrane perforation, swelling, and rupture, along with the release of pro-inflammatory factors such as IL-1β and IL-18.^62,63^ Cathepsin L activated Caspase-3/GSDME-induced pyroptosis of PAECs, dependent on BMPR2 degradation and subsequent Smad1 downregulation, contributing to pulmonary vascular remodeling of MCT-PAH and SU5416-hypoxia-induced PAH (Su/Hx-PAH) rats.^64^

### The role of BMPR2 in sex difference in PAH

PH presents a puzzling sex bias, being more prevalent in women yet often less severe than in men.^65^ The underlying reasons remain unclear, but this difference could be attributed, at least in part, to the influence of sex hormones.^66,67^ Sex hormones may play a role in regulating BMPR2 and PAH. Dehydroepiandrosterone enhances BMPR2 expression in PASMCs from PAH patients by inhibiting STAT3 activity.^68^ Conversely, the estrogen metabolite 16α-hydroxyestrone exacerbates the effects of the *Bmpr2*^*R899X*^ mutation, leading to increased miR-29 levels in mouse lungs, decreased expression of CD36 and PPARγ in PMVECs, and heightened ceramide deposition in lung tissues, contributing to aberrant cellular metabolism and promoting PAH in *Bmpr2* mutant mice.^69^ The contrasting effects of 16α-hydroxyestrone and dehydroepiandrosterone underscore the importance of hormonal metabolic homeostasis for lung tissue and pulmonary vascular health.

## TGF-β signaling pathway in PH

TGF-β as a key member of the TGF-β superfamily, binds to type I (ALK1-7 or TGFβR1) and type II receptors (TGFβR2), leading to the phosphorylation of the kinase domain of the type I receptor by the type II receptor. This activation initiates two types of downstream signaling: canonical (Smads) and non-canonical (PI3K/Akt, MAPK, and RhoA GTPases).^52,70^ Smads which mediate TGF-β signaling, can be categorized into three functional groups: R-Smads, Co-Smads, and I-Smads. R-Smads (Smad1, 2, 3, 5, and 8) are phosphorylated by type I receptors, forming heterodimeric complexes with Co-Smad (Smad4) before translocating to the nucleus to regulate target gene transcription. In contrast, I-Smads (Smad6 and Smad7) negatively regulate TGF-β signaling by competing with R-Smads for receptor binding or promoting receptor degradation.^71^

Perkett et al. first identified TGF-β in sheep lung lymph, noting its increased expression during early PH development.^72^ Recent studies indicate that PH may arise from an imbalance in TGF-β signaling within pulmonary vascular cells. Furthermore, TGF-β is implicated in various pathological processes associated with PH, including cell proliferation, apoptosis, metabolic dysregulation, mitochondrial dysfunction, inflammation, immunomodulation, ECM remodeling, fibrosis, and EndMT (Fig. 2b).

### TGF-β promotes anti-apoptosis and cell proliferation in PH

#### Canonical pathway

Increased expression of various TGF-β superfamily ligands (activins, GDFs), as well as TGF-β1 and 3) in distal small pulmonary arteries of IPAH patients and *BMPR2* mutation hypoxia-PH, activate Smad2/3 and Smad1/5/9 pathways, enhancing proliferation and reducing apoptosis of PMVECs and PASMCs.^73^ In MCT-PAH rats, miR-125a-5p downregulation and TGF-β/IL-6 upregulation increases STAT3, Smad2/3, PCNA, Survivin, and Bcl-2, driving proliferation and anti-apoptotic effects.^74^ In MCT-PAH rats, key components of the TGF-β signaling pathway such as TGFβR2, endoglin, ActA receptor type I, Smad3, and Smad4 are downregulated in lung tissues. Additionally, reduced expression of Smad2 and Smad3 in PASMCs leads to increased expression of PCNA and subsequent PASMC proliferation.^75^ Notably, the downregulation of Smad2 and Smad3 in the later stages of the disease is attributed to prolonged exposure to TGF-β.

Hypoxia activates the TGF-β/Smad2/3 pathway to induce eNOS dysfunction in human PAECs.^76^ Extracellular vesicles (EVs) released by HIV-infected human monocyte-derived macrophages elevate TGF-β levels and enhance Smad2 phosphorylation in rat PASMCs and PAECs. This results in increased expression of ET-1, TNF, and cTnI, promoting PASMC proliferation and PAEC apoptosis, ultimately contributing to HIV-related PH.^77^

In bovine PAECs, TGF-β stimulation activates the ALK5 receptor, resulting in increased phosphorylation of Smad2 and Smad1/5, leading to elevated levels of Bcl-2 and decreased Caspase-3 activity, which subsequently inhibits apoptosis.^78^ Conversely, in rat PMVECs, TGF-β activation of ALK5 reduces Bcl-2 and cFLIP levels, increases Caspase-8 activity, and promotes mitochondrial permeabilization, driving apoptosis.^79^ Notably, Lu et al. found that TGF-β promotes apoptosis in bovine PMVECs, indicating that the varied responses of ECs to TGF-β result from phenotypic differences among ECs in different regions of the pulmonary vascular system, rather than interspecies variations.^79^

### TGF-β promotes ECM remodeling and collagen deposition in PH

#### Canonical pathway

Research indicates that Calpain is linked to TGF-β activity. In lung tissues of MCT-PAH rats, EGF levels were elevated, and Calpain-1 and Calpain-2 were upregulated.^80^ Additionally, Calpain-4 was upregulated in the lungs of mice with hypoxia-PH.^80^ These changes resulted in increased levels of PDGF, activation of the TGF-β1/Smad2/3 pathway, excessive deposition of COL1, and thickening of the smooth muscle layer of pulmonary arterioles, contributing to pulmonary vascular remodeling and the development of PH.^80^

5-hydroxytryptamine (5-HT) in rat PAFs induces ECM remodeling through binding to 5-HT2A, upregulation of TGF-β1, and increased Smad2/3 phosphorylation, leading to an increase in COL1, FN1 content.^81^

In Su/Hx-PAH mice, upregulated Legumain activates extracellular MMP-2, leading to upregulation of TGF-β1 and increased Smad2/3 phosphorylation in PASMCs, which results in deposition of ECM proteins (COL1, FN1, and tenascin C) in pulmonary arteries.^82^

#### Noncanonical pathway

In lung homogenates from *Fra-2* transgenic mice, upregulation of TGF-β leads to increased JUN-B expression and binding of the AP-1 complex to the *meprin* β promoter, resulting in elevated meprin β expression. Meprin β, a metalloproteinase that processes procollagen, promotes collagen deposition and enhances protofibril assembly. This process decreases MMP-2 and MMP-9 levels while increasing COL1, ultimately inducing ECM remodeling.^83^

### TGF-β promotes mitochondrial metabolic reprogramming and altered kinetics in PH

In MCT-PAH and Su/Hx-PAH rat PASMCs, platelet-derived TGF-β increases the expression of PKM2 through the mTOR/c-Myc/PTBP1-hnRNP A1 signaling pathway. This pathway facilitates the conversion of PKM1 isoforms to PKM2, which is essential for shifting cellular metabolism towards aerobic catabolism, thereby enhancing cellular aerobic glycolysis.^84,85^ In RV fibroblasts (RVFs) from MCT-PAH rats, there is an elevated expression of TGF-β, CTGF, and Drp1. This is accompanied by a significant increase in mitochondrial fragmentation, decreased oxygen consumption rate, adenosine triphosphate (ATP) production, maximal respiratory rate, and reserve respiratory capacity, as well as increased lactate production and glucose consumption.^86^

In rat PAECs stimulated with TGF-β, upregulation of IP3R3 enhances calcium ion release from the endoplasmic reticulum. The influx of calcium ions into the mitochondria increases mitochondrial ROS levels and decreases ΔΨm and the activity of complexes I, III, IV, and V, impairing mitochondrial function.^87^ In sheep PAECs, TGF-β decreases PPARγ, which increases NOX activity and NOX-derived superoxide. This leads to Akt1 nitration and elevated p617eNOS levels, causing a redistribution of eNOS in mitochondria. Consequently, there is increased nitrifying CrAT, upregulation of acyl levulinic acid, heightened mitochondrial ROS levels, and decreased Δψm. These changes result in significantly reduced maximum and reserve respiratory capacities, lower ATP levels, and diminished eNOS-hsp90 interactions, impairing NO release and causing cell dysfunction.^88^

### TGF-β promotes inflammation in PH

#### Canonical pathway

Upregulation of TGF-β1/3 and PAI-1 expression in lung tissues of MCT-PAH rats increases Smad2 phosphorylation, leading to increased expression of IL-6, IL-1β, and ICAM.^89^ Bouchet et al. found that stimulating human PASMCs with IL-1β and PAECs with H_2_O_2_ upregulates TGF-β1. This leads to phosphorylation of Smad3 and p38 in human PASMCs, while only p38 phosphorylation occurs in human PAECs.^90^

#### Non-canonical pathway

In PAECs from rats with a *Bmpr2* mutation, the TGF-β/Smad2/3 signaling pathway is enhanced, leading to increased nucleoplasmic expression of 5-LO. This results in elevated transcripts of *Il1r1, Il6r, Tlr2*, and *Tlr4*, and activation of the Smad2/3-dependent and p38-dependent TGF-β pathways through 5-LO-mediated inflammation. This cascade promotes increased synthesis of endogenous leukotriene B_4_, 5-LO nuclear envelope translocation, and the development of PAECs.^91^

### TGF-β promotes EndMT in PH

#### Canonical pathway

In MCT-PAH rats, upregulation of TGF-β and increased phosphorylation of Smad2 leads to elevated expression of Gal-3 and α-SMA, facilitating EndMT.^92^ Furthermore, the neuroblastoma suppressor of tumorigenicity 1, a secreted glycoprotein associated with congenital heart disease-related PH, exacerbates the phenotypic transformation of PASMCs. In *neuroblastoma suppressor of tumorigenicity 1*-knockout rats and human PAECs subjected to left cervical shunt and right pulmonary artery ligation, TGFβR2 levels increase, and Smad2 phosphorylation is enhanced. This leads to decreased cadherin-5 and eNOS levels, along with increased expression of α-SMA and vimentin, promoting EndMT.^93^

Over-expression of *Twist1* by transduction of human PAECs with lentivirus activates the TGF-β/Smad2/3 pathway. This process results in the upregulation of α-SMA, Slug, and vimentin expression and downregulation of endothelial markers CD31 and vascular endothelial-cadherin expression,^94^ which can ultimately induce EndMT.

In a neonatal mouse model exposed to hyperoxia for 72 h and subsequently returned to normoxia, time-dependent vascular remodeling was observed.^95^ After 60 days, a significant reduction in pulmonary artery numbers and an increase in the Fulton index and RVSP were noted, indicating RV remodeling and PH.^95^ Primary mouse PMVECs and mouse fetal lung EC lines (MFLM-91U) exposed to hyperoxia exhibit increased phosphorylation of Smad2 and Smad3, alongside downregulation of Smad7. This results in elevated levels of α-SMA, vimentin, and Snail2 (which inhibits VE-cadherin transcription), and decreased levels of vWF and PECAM1, ultimately, promoting EndMT.^95^ Knockdown of *MEKK3* in human umbilical vein endothelial cells (HUVECs) increases the expression of TGFβR1, TGFβR2, TGF-β, phosphorylated Smad2/3, SM22α, and α-SMA. This induces EndMT and contributes to the inward remodeling of pulmonary and systemic arteries, ultimately leading to primary PAH and systemic arterial hypertension.^96^

### TGF-β promotes cell migration in PH

Hypoxia-induced TGF-β binds to the ALK5 receptor in human PASMCs, resulting in upregulation of Smad3. This leads to increased expression of miR-143/145 and secretion of exosomes enriched in miR-143, which promote the migration of PAECs.^97^ Additionally, Gal-3 stimulates TGF-β expression and increases Smad2/3 phosphorylation in PASMCs, further enhancing proliferation and migratory capacity.^98^

### Differences in TGF-β/Smad signaling across PAH models and patients

In the lung tissues of patients with PH-LHD, the expressions of the elastin-degrading enzymes cysteine cathepsin and metalloproteinase with thrombospondin motifs (ADAM-4) are upregulated, which promotes the degradation of elastin. This leads to an increase in the content of TGF-β.^44^

### Sex differences in TGF-β signaling and susceptibility to PAH

Research indicates that TGF-β signaling can affect X chromosome inactivation in women, contributing to an imbalance in TGF-β signaling in PAH.^99^ Exposure to hyperoxia resulted in more pronounced EndMT in human PMVECs from male donors compared to those from female donors.^95^ Likewise, male mice showed a greater susceptibility to neonatal hypoxia-PH in animal models.^95^

## HIF signaling pathway in PH

In 1999, Yu et al. first discovered that hypoxia-induced damage to vascular remodeling in *Hif-1*α^+/-^ mice was reduced, and the onset of PH was delayed; In 2003, Brusselmans et al. first discovered that *Hif-2*α^+/−^ mice exhibited a lack of pulmonary vascular remodeling during hypoxia and were protected from PH.^100,101^ HIF consists of three subtypes: HIF-1, HIF-2, and HIF-3, and serves as a key regulator in sensing and adapting to cellular oxygen levels.^102,103^ HIF comprises an oxygen-sensitive α subunit and an oxygen-insensitive β subunit.^104,105^ In cells with adequate oxygen and iron (Fe^2+^), two distinct sites, Pro402 and Pro564, within the oxygen-dependent degradation domain of the α subunit, are hydroxylated by PHD1, PHD2, and PHD3 in a process that depends on Fe^2+^ and oxygen.^106^ The β domain of the tumor suppressor protein then directly binds to the hydroxylated α subunit and acts as a recognition component of the E3 ubiquitin ligase complex to promote the hydrolysis of the α subunit.^104,105,107–110^ The asparagine hydroxylase, which relies on both Fe^2+^ and 2-oxogluconate, hydroxylates asparagine residues to prevent p300 from binding to the hypoxia-induced COOH terminal activation domain or directly competing with p300 for COOH terminal activation domain binding to inhibit the transcriptional activity of HIF.^104,105,107–110^ In the absence of oxygen or Fe²⁺, hydroxylation of the α subunit is blocked, which prevents its degradation and allows it to accumulate. This subunit then migrates to the nucleus to form a heterodimer with the β subunit. Additionally, inhibition of asparagine hydroxylation enables p300 to bind to the COOH-terminal activation domain, promoting HIF transcription.^104,105,110^ Although HIF-1α and HIF-2α share structural similarities, they differ in tissue distribution and function. HIF-1α primarily regulates the growth of human PASMCs, whereas HIF-2α is more involved in the growth and remodeling of human PAECs, significantly influencing angiogenic spheroid formation.^111^

**Fig. 3:**
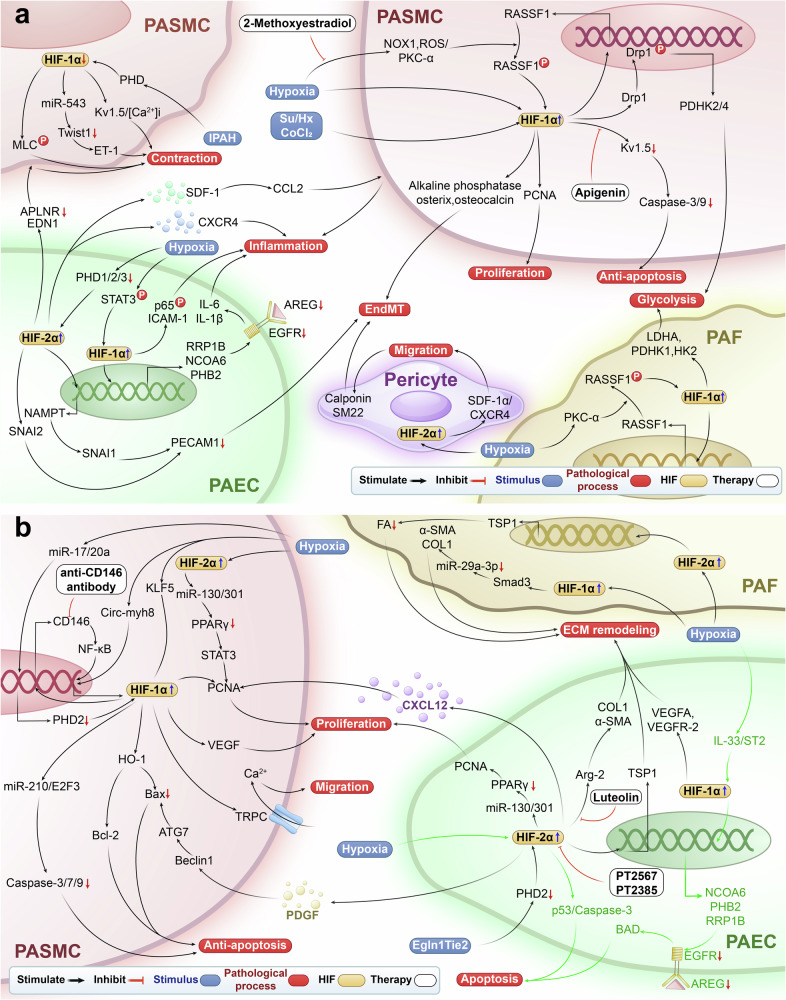
HIF signaling pathway and targeted therapy in PH. **a** The involvement of the HIF signaling pathway in cell proliferation, apoptosis, anti-apoptosis, ECM remodeling, migration, and cell-cell interactions in PH. Hypoxia and Tie2Cre-mediated Egln1 deletion upregulate HIF-1α and HIF-2α, leading to increased ECM protein deposition. In PASMCs, this results in the upregulation of PCNA and intracellular calcium ion concentration, along with the downregulation of Caspase-3/7/9, promoting cell proliferation, migration, and resistance to apoptosis. In PAECs, the same conditions induce the upregulation of Caspase-3, promoting apoptosis. Luteolin, PT2567, PT2385 and anti-CD146 antibody mitigate PH by regulating HIF signaling. **b** HIF signaling pathway also plays a crucial role in EndMT, glycolysis, inflammation, contraction, and cell-cell interactions in PH. Hypoxia, Su/Hx, and cobalt chloride upregulate HIF-1α and HIF-2α, resulting in metabolic reprogramming (e.g., PDHK), upregulation of IL and COL1, promoting inflammation, EndMT, which influences anti-apoptosis, proliferation, and migration. Conversely, the downregulation of HIF-1α in IPAH patients may lead to PASMC contraction. 2-Methoxyestradiol and apigenin mitigate PH by regulating HIF signaling. *PAEC* pulmonary artery endothelial cell, *PASMC* pulmonary artery smooth muscle cell, *PAF* pulmonary artery fibroblast, *HIF-1α* hypoxia-inducible factor-1 alpha, *HIF-2α* hypoxia-inducible factor-2 alpha, *COL1* type I collagen, *KLF5* Kruppel-like factor 5, *Circ-myh8* circ_chr11_67292179-67294612, *NF-κB* NF-kappaB, *PPARγ* peroxisome proliferator-activated receptor gamma, *STAT3* signal transducer and activator of transcription 3, *E2F3* E2F transcription factor 3, *HO-1* heme oxygenase-1, *Bcl-2* B-cell lymphoma 2, *Bax* Bcl-2 associated X, *ATG7* autophagy-related gene 7, *VEGFR-2* vascular endothelial growth factor receptor 2, *VEGFA* vascular endothelial growth factor A, *PCNA* proliferating cell nuclear antigen, *Arg-2* arginase-2, *TSP1* thrombospondin 1, *IL-33/ST2* interleukin 33/the suppression of tumorigenicity 2 receptor, *NCOA6* nuclear receptor co-activator 6, *PHB2* prohibitin 2, *RRP1B* ribosomal RNA processing 1 homolog B, *AREG* amphiregulin, *EGFR* epidermal growth factor receptor, *BAD* BCL2-associated agonist of cell death, *FA* focal adhesion, *IL-6* interleukin 6, *IL-1β* interleukin-1beta, *Twist1* Twist-related protein 1, *ICAM-1* intercellular adhesion molecule-1, *CXCR4* C-X-C chemokine receptor 4, *SDF-1* stromal cell-derived factor 1, *EDN1* endothelin 1, *APLNR* apelin receptor, *NAMPT* nicotinamide phosphoribosyltransferase, *PECAM1* platelet endothelial adhesion molecule 1, *ROS* reactive oxygen species, *NOX1* NADPH oxidase 1, *PKC-α* protein kinase C alpha, *RASSF1* Ras association domain family 1, *Drp1* dynamin-related protein 1, *Kv1.5* Kv1.5 channels, *PDHK* pyruvate dehydrogenase kinase, *IPAH* diopathic pulmonary arterial hypertension, *MLC* myosin light chain, *ET-1* endothelin-1, *SM22* smooth muscle 22, *PHD* proline hydroxylase, *Smad3* small mother against decapentaplegic family member 3, *CXCL12* C-X-C chemokine ligand 12, *α-SMA* α-smooth muscle actin, *CCL2* C-C motif ligand 2, *CoCl*_2_ cobalt chloride, *Su/Hx* SU5416-hypoxia, *Egln1Tie2* Tie2Cre-mediated Egln1 deletion, *PDGF* platelet-derived growth factor, *Snail* Snail family transcriptional repressor, *AK4* adenylate kinase 4, *VEGF* vascular endothelial growth factor, *[Ca*^2+^*]i* intracellular Ca^2+^ concentrations, *miR* microRNA, *p65* a protein subunit of NF-kappaB, *Caspase* cysteinyl aspartate specific proteinase, *siRNA* small interfering RNA, *CD146* cluster of differentiation 146, *Ca*^2+^ calcium ion, *p53* tumor protein p53

Some studies indicate that HIF-1α protein expression is reduced in PASMCs of patients with IPAH.^112,113^ In yaks, HIF-1α levels in the lungs increase with age.^114^ Similarly, increased HIF-1α levels have been observed in the smooth muscle of the distal pulmonary artery in adult male Sprague Dawley rats exposed to hypoxia.^115^ Conversely, HIF-2α protein expression is elevated in lung parenchyma and pulmonary artery samples from patients with IPAH.^116,117^ Additionally, the absence of HIF-2α in the PAECs of mice can prevent hypoxia-PH.^118^ The above evidence indicates that the activation or inhibition of HIF-1α and the activation of HIF-2α are linked to various pathological processes, such as cell proliferation, apoptosis, ECM remodeling, EndMT, mitochondria dysfunction, metabolic reprogramming, inflammation, vascular constriction, and relaxation, which may play a crucial role in PH (Fig. 3).

### HIF promotes endothelial apoptosis in PH

Hypoxia upregulates HIF-1α and HIF-2α expression to promote apoptosis in PAECs. In hypoxic human PAECs, increased HIF-1α upregulates *NCOA6*, *PHB2*, and *RRP1B* genes, leading to downregulation of AREG/EGFR, thereby increasing Bcl-2-associated cell death agonist to promote apoptosis.^119^ Additionally, hypoxia upregulates HIF-2α in PAECs, leading to enhanced expression of the p53 protein, which subsequently increases the levels of cleaved Caspase-3 and promotes apoptosis.^120^

### HIF promotes cell proliferation in PH

Hypoxia leads to the upregulation of HIF-1α, promoting the proliferation of PASMCs. However, in the later stages, an adaptive response inhibits HIF-1α, thereby limiting PASMC proliferation. In PASMCs, HIF-1α binds directly to the *hypoxia-responsive elements (HRE)* in the *CD146* promoter, upregulating CD146 during hypoxia. This accumulation and dimerization of CD146 enhance *HIF-1*α transcription via NF-κB activation, creating a feedback loop between CD146 and HIF-1α. This cross-regulation increases the expression of COL1, FN1, and vimentin, ultimately promoting cell proliferation.^121^ In hypoxia-induced PASMCs, over-expressed Circ-myh8 recruits KAT7 to the promoter of the *Hif-1*α gene, resulting in acetylation of H4K5 and subsequent activation of HIF-1α transcription, which in turn upregulates PCNA and ultimately induces proliferation.^122^ Interestingly, research has shown that in the early stages of hypoxia-induced human and mouse PASMCs, the 3’-untranslated region of miR-17/20a directly targeted and primarily inhibited PHD2, leading to HIF-1α degradation to promote VEGF expression, and subsequently enhances proliferation.^123^ In the late stages, however, the expression of miR-17 and miR-92 is suppressed, resulting in increased PHD2 levels to limit HIF-1α activity, and subsequently reduces VEGF expression, thereby slowing the progression of PH.^123^

Hypoxia increases HIF-2α expression in PAECs and PASMCs, promoting cell proliferation. In the co-culture of PAECs from Tie2Cre-mediated disruption of Egln1 mice, elevated HIF-2α levels enhance PASMC proliferation. Under hypoxic conditions, elevated HIF-2α upregulates POU5F1/Oct4, which induces the expression of the miR-130/301 family and inhibits PPARγ. This results in downregulation of the APLN-miR-424/503-FGF2 axis in PAECs and upregulation of the STAT3-miR-204 pathway in PASMCs, ultimately increasing PCNA levels and promoting cell proliferation.^124^ In PAECs from Tie2Cre-mediated disruption of Egln1 mice, decreased PHD2 expression leads to HIF-2α upregulation and subsequent release of CXCL12. This induces increased PCNA expression in PASMCs during co-culture, ultimately promoting proliferation.^125^

### HIF promotes anti-apoptosis of PASMCs in PH

Hypoxia upregulates HIF-1α and HIF-2α expression to inhibit apoptosis in PASMCs. In hypoxic human PASMCs, increased KLF5 enhances HIF-1α levels, which decreases the Bax/Bcl-2 ratio and reduces the expression of cleaved Caspase-3 and Caspase-9, promoting anti-apoptotic effects.^115^ Similarly, hypoxia induces PASMCs to upregulate HIF-1α, which decreases the Bax/Bcl-2 ratio by upregulating HO-1, ultimately leading to the inhibition of apoptosis.^114^ Additionally, HIF-1α upregulates miR-210, which directly downregulates E2F3, leading to reduced Caspase-3 and Caspase-7 activity and inducing anti-apoptotic effects in hypoxic human PASMCs.^126^

Hypoxia elevates HIF-2α in PAECs, which activates a paracrine pathway to induce anti-apoptotic effects in PASMCs. Specifically, hypoxia upregulates HIF-2α in PAECs, leading to enhanced expression of the p53 protein, which subsequently increases the levels of cleaved Caspase-3 and promotes apoptosis.^120^ Under hypoxic conditions, upregulated HIF-2α in PAECs increases PDGF in PASMCs, which in turn upregulates Beclin1 and ATG7, inhibiting Bax expression and ultimately promoting anti-apoptotic effects.^127^ Furthermore, the research by Hu et al. revealed that under hypoxic conditions, the elevation of HIF-2α mediates the activation of PAECs, which results in the upregulation of *Bcl-2*, *Bcl2l1*, and *Birc5* gene expression in PASMCs, ultimately leading to anti-apoptotic effects in PASMCs.^128^

### HIF promotes ECM remodeling in PH

Hypoxia-induced HIF-1α upregulation enhances the angiogenic capacity of PAECs and promotes the migration of PASMCs and PAFs. In human PAECs, hypoxia promotes the expression of HIF-1α by upregulating the IL-33/ST2 axis, which then increases the expression of VEGFA and VEGFR-2, leading to enhanced adhesion and angiogenesis.^129^ In chronic hypoxia-induced PASMCs, increased HIF-1α upregulates TRPC expression, thereby activating the SOCs to increase the entry of stored and transported calcium, thereby elevating intracellular calcium concentration to facilitate migration.^130^ In PAFs, hypoxia upregulates HIF-1α and subsequently downregulates miR-29a-3p expression by increasing Smad3, thereby increasing α-SMA and COL1 expression to promote migration.^131^

The elevated levels of HIF-2α in *R200WVhl* mutation mice promote the involvement of myofibroblasts in ECM remodeling. Hypoxia-induced HIF-2α drives both human PAECs and myofibroblasts to participate in this remodeling process. *R200WVhl* mutation mice, which have a mutation in codon 200 of the VHL tumor suppressor protein, develop PH that resembles the condition observed in Chuvash patients.^132^ In the lungs of *Vhl*^*R/R*^ mice, elevated HIF-2α activity leads to an upregulation of CXCL12 expression, thereby stimulating an increase in FN1 production in myofibroblasts by enhancing the infiltration of inflammatory cells, primarily macrophages, ultimately promoting fibrosis.^132^ Additionally, in human PAECs, hypoxia-induced HIF-2α binds to the *HRE* in the proximal promoter region of *TSP-1*, to drive vascular remodeling by destabilizing cell connectivity and increasing cell permeability.^116^ In hypoxic PAECs, increased levels of HIF-2α promote the expression of Arg-2, elevating the content of collagen and α-SMA, ultimately contributing to vascular and ECM remodeling.^118^ Moreover, hypoxia can stimulate the breakdown of fatty acids in myofibroblasts via the HIF-2α/TSP-1 pathway, thereby reducing adhesion to FN substrates and facilitating cell migration.^116^

### HIF promotes mitochondria dysfunction and metabolic reprogramming in PH

Hypoxia in PASMCs triggers an increase in HIF-1α expression, which causes mitochondrial structural and functional abnormalities, ultimately inhibiting apoptosis. In PASMCs of hypoxic rats, elevated HIF-1α lowers the Bax to Bcl-2 expression ratio by suppressing Kv1.5 expression, thereby triggering the release of cytochrome C from the mitochondria into the cytoplasm, leading to the activation of Caspase-3 and Caspase-9, ultimately inhibiting mitochondria-dependent apoptosis.^133^

On the contrary, mitochondrial structural and functional abnormalities can also induce HIF-1α expression to promote PASMC proliferation. In rats with IPAH, chromosomal abnormalities on chromosome 1 result in reduced expression of electron transport chain complexes I, III, SOD2, and COX4 in the mitochondria of PASMCs, lowers overall ROS production, promoting the nuclear translocation of HIF-1α, which subsequently decreases Kv1.5 expression.^134^ However, the decreased expression of Kv1.5 in PASMCs of hypoxic rats inhibited Caspase-3, thereby preventing apoptosis.^135^ Moreover, hypoxia-induced dephosphorylation of FUNDC1 in PASMCs strengthened its interaction with LC3B-II, resulting in enhanced mitochondrial autophagy. This, in turn, elevated ROS production, which increased HIF-1α levels and ultimately upregulated PCNA expression, promoting cell proliferation.^136^

In PASMCs and PAFs, elevated HIF-1α promotes glycolysis through various pathways. HIF-1α is upregulated in PASMCs of chronic cobalt chloride-induced PAH rats, which phosphorylates Drp1 by increasing Cyclin B/CDK1, subsequently leading to increased expression of the *PDHK2* and *PDHK4* genes, resulting in increased lactate production and promotion of glycolysis.^137^ In PASMCs, hypoxia-induced mitochondrial ROS inactivates PHD3, leading to upregulation of HIF-1α, which subsequently promotes α-subunit phosphorylation of PDH-E1 through increased PDHK1 and PDHK, thereby increasing glucose consumption and lactic acid accumulation.^138^ Additionally, in PASMCs and PAFs, hypoxia activates NOX1, raising ROS levels and triggering PKC-α activation, which phosphorylates and stabilizes RASSF1A, preventing HIF-1α degradation.^139^ Meanwhile, HIF-1α binds to *HRE* in the *RASSF1A* promoter, creating a feedforward loop that activates PDHK1, HK2, and LDHA, further increasing lactic acid production and promoting glycolysis.^139^ HIF-2α primarily regulates lipid metabolism and oxidative stress, but its involvement in mitochondrial abnormalities and metabolic reprogramming requires further investigation.

### HIF promotes inflammation in PH

According to Florentin et al., the upregulation of HIF-1α downregulates AREG/EGFR by promoting *NCOA6, PHB2*, and *RRP1B* gene expression, thereby elevating IL-1β, IL-6, TNF, and IFN-β expression, ultimately leading to the aggregation of inflammatory white blood cells.^119^ In addition, strong HIF-1α expression was detected in inflammatory cells in patients with severe PH, especially alveolar macrophages.^140^ HIMF, after acting on *Hif-1*α^+/+^ mice, promotes the expression of IL-6 in macrophages by upregulating HIF-1α and aggregation into the reconstructed pulmonary small blood vessels.^141^ After exposing immature mouse bone marrow-derived macrophages to intact pulmonary artery explants from hypoxia-PH calves, the bone marrow-derived macrophages exhibited upregulation of STAT3, HIF-1α, and C/EBPβ. This occurred in response to increased IL-6 expression in PAFs, leading to enhanced transcription of *CD163, CD206, SOCS3*, and *IL4RA*, which ultimately polarized the bone marrow-derived macrophages into M2 macrophages.^142^

Hypoxia-induced upregulation of HIF-2α can enhance the release of inflammatory mediators from PAECs. In turn, activated PAECs stimulate PASMCs to release inflammatory mediators, which promotes macrophage accumulation. In hypoxia-PH rats, the increased expression of HIF-2α in PAECs boosts the expression of SDF-1, CXCR4, and ICAM-1, and the elevated SDF-1 levels in PAECs promote the expression of CCL2 in PASMCs, initiating the recruitment of macrophages.^128^

### HIF promotes EndMT in PH

The elevation of HIF-1α facilitates the transformation of hypoxic human PAECs and PASMCs of patients with PAH into mesenchymal cells. In cultured Su/Hx-PAH PASMCs in vitro, reduced miR-204 upregulates HIF-1α through the expression of RUNX2. This, in turn, upregulates alkaline phosphatase, osteocalcin, and osterix, leading to the differentiation of osteoblast-like cells that contribute to the mineralization of the blood vessel wall.^143^

The elevation of HIF-2α can drive the transformation of PAECs and pericytes into mesenchymal cells. Under hypoxic conditions, the inactivation of PHD2 in human PAECs increases HIF-2α, thereby enhancing *NAMPT* promoter activity to raise the expression of Snail1 and decrease the expression of PECAM1, ultimately inducing EndMT.^144^ The mRNA expression of PHD1, PHD2, and PHD3 are decreased in PAECs induced by hypoxia, resulting in the stabilization and increased expression of HIF-2α, which downregulates PECAM1 by upregulating Snail1 and Snail2, ultimately promoting EndMT.^145^ Under hypoxia, elevated HIF-2α upregulates SDF-1α/CXCR4 in pericyte cells, promoting pericytes to migrate from capillaries to arterioles, where they increase the expression of Calponin and SM22, transforming into smooth muscle cell (SMC)-like cells and contributing to vascular remodeling.^146^

### HIF regulates vasoconstriction in PH

HIF-1α contributes to vasoconstriction in PASMCs of patients with IPAH. The expression of HIF-1α is diminished in PASMCs from IPAH patients.^113^ This results in increased expression of miR-543, which subsequently upregulates Twist1 and enhances the expression of ET-1, ultimately promoting vasoconstriction.^113^ In patients with IPAH, elevated levels of O_2_ and ascorbate lead to increased PHD activity and reduced HIF-1α protein levels. This in turn decreases Kv1.5 protein expression, raises intracellular calcium levels, and promotes the dephosphorylation of MLC kinase, enhancing the activity of phosphorylated MLC and resulting in increased contractility of PASMCs.^112^ Hypoxia-induced upregulation of HIF-2α in human PAECs promotes vascular constriction. Hypoxia leads to the inactivation of PHD2 in human PAECs, resulting in the accumulation of HIF-2α. This accumulation causes dysregulation of vasodilation and contraction by upregulating ET-1 expression while inhibiting APLN receptor expression, ultimately triggering PH.^147^

## MAPK signaling pathway in PH

MAPK is a serine-threonine protein kinase that gets activated by various stimuli, including cytokines, growth factors, neurotransmitters, hormones, cell stress, and cell adhesion. It plays a key role in regulating gene expression, mitosis, metabolism, motility, survival, apoptosis, and differentiation. The MAPK family is evolutionarily conserved and operates through a phosphorylation cascade, with MAPK activation occurring via the phosphorylation of tyrosine and threonine residues, catalyzed by members of the MAPK/MEK family.^148,149^ In turn, MEKs are activated by serine/threonine phosphorylation catalyzed by a range of different protein kinases (collectively referred to as MAPK kinase).^149^ The three main pathways of MAPK signaling are named after the terminal kinases in each cascade, namely ERK, p38, and JNK. The wide range of functions regulated by MAPK is mediated by the phosphorylation of several substrates, including members of the protein kinase family called MKs, such as MSK, and MK2/3.

**Fig. 4:**
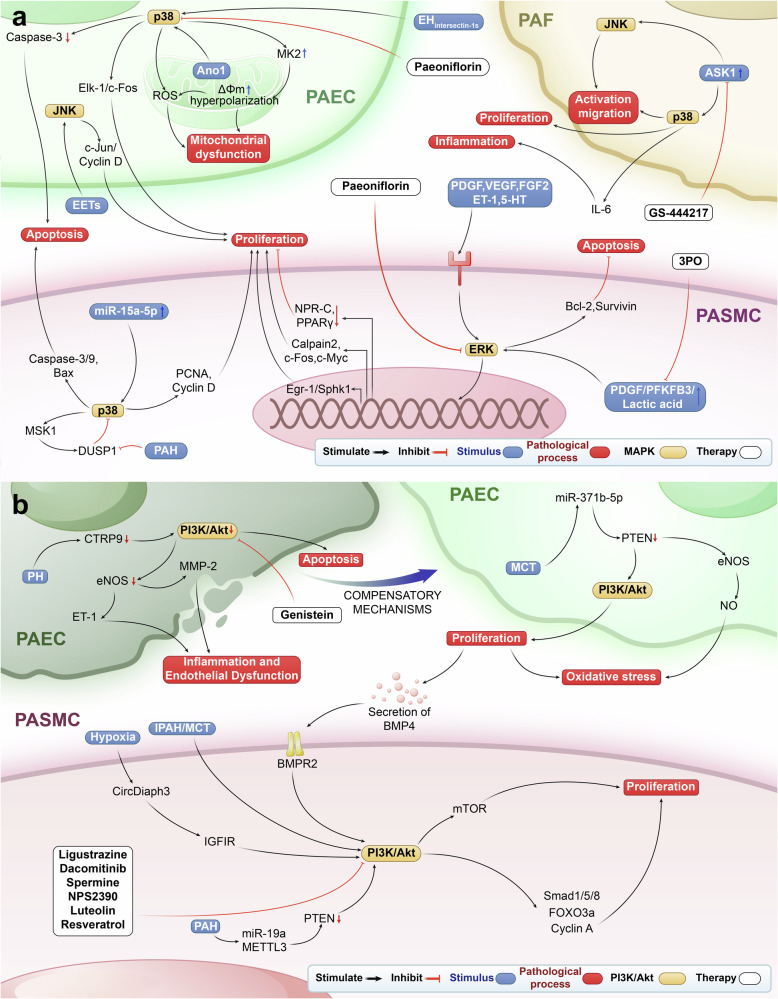
MAPK and PI3K/Akt signaling pathways in PH. **a** MAPK signaling pathway and targeted therapy in PH. MAPK pathway is activated by cytokines and other stimuli, leading to the upregulation of cell cycle proteins and subsequently promoting proliferation. The ERK pathway inhibits cell apoptosis, while the p38 pathway promotes cell apoptosis. Activated p38 signaling contributes to mitochondrial dysfunction and also affects inflammation by inducing the secretion of inflammatory factors. ASK1 activates p38 and JNK, thereby stimulating PAF activation, migration, and proliferation. Paeoniflorin, and 3PO alleviate PH by inhibiting ERK and its related pathways, paeoniflorin by inhibiting p38 and its related pathways, and GS-444217 alleviate PH by inhibiting ASK1/JNK/p38 axis. *PASMC* pulmonary artery smooth muscle cell, *PAEC* pulmonary artery endothelial cell, *PAF* pulmonary artery fibroblast, Δ*ψm* mitochondrial membrane potential, *ERK* extracellular signal-regulated kinase, *JNK* c-Jun N terminal kinase, *PPARγ* peroxisome proliferator-activated receptor γ, *PFKFB3* 6-phosphofructo-2-kinase/fructose-2,6-biphosphatase 3, *PDGF* platelet-derived growth factor, *ET-1* endothelin-1, *PCNA* proliferating cell nuclear antigen, *5-HT* 5-hydroxytryptamine, *EETs* epoxyeicosatrienoic acids, *Ano1* Anoctamin-1, *NPR-C* atrial natriuretic peptide clearance receptor, *VEGF* vascular endothelial growth factor, *FGF2* fibroblast growth factor 2, *ASK1* apoptosis signal-regulating kinase 1, *ROS* reactive oxygen species, *MK2* mitogen-activated protein kinase -activated protein kinase 2, *Elk-1* ETS-like transcription factor, *IL* interleukin, *Egr-1* early growth response protein 1, *Bcl-2* B-cell lymphoma 2, *Bax* Bcl-2 associated X, *MSK1* mitogen and stress-activated kinase 1, *DUSP1* dual specificity phosphatase-1, *SphK1* sphingosine kinase 1, *Caspase* cysteinyl aspartate specific proteinase, *miR* microRNA, *3PO* 3-(4-(trifluoromethyl phenyl)-1H-pyrazole. **b** PI3K/Akt signaling pathway and targeted therapy in PH. CTRP9 downregulation reduces PI3K/Akt in PAECs, causing apoptosis, inflammation (ET-1, MMP-2), and dysfunction. In late PH, miR-371b-5p/PTEN downregulation activates PI3K/Akt, promoting proliferation and oxidative stress (eNOS/NO) in PAECs. BMP4-induced activation of BMPR2/PI3K/Akt in PASMCs activates PI3K/Akt in PASMCs. PI3K/Akt activation in PH drives inflammation (NF-κB) and proliferation (Smad1/5/8, FOXO3a, Cyclin A) in PAECs and PASMCs. Inhibitors like Nobiletin and Resveratrol mitigate PH, while agent like Genistein counteract early PH. *PI3K* phosphoinositide 3-kinase, *Akt* protein kinase B, *CTRP9* C1q/TNF-related protein 9, *eNOS* endothelial nitric oxide synthase, *MMP-2* matrix metalloproteinase-2, *BMP4* bone morphogenetic protein 4, *BMPR2* bone morphogenetic protein receptor type 2, *PTEN* phosphatase and tensin homolog, *NF-κB* nuclear factor kappa B, *ECM* extracellular matrix, *MCT* monocrotaline, *IPAH* idiopathic pulmonary arterial hypertension, *CircDiaph3* circular RNA diaphanous-related formin 3, *IGFIR* insulin-like growth factor 1 receptor, *miR* microRNA

In 1998, Scott et al. discovered that hypoxia stimulated PAFs to activate JNK and p38.^150^ Dysregulation of MAPK signaling has been noted in various experimental models of PAH and PASMCs from patients with *BMPR2* mutations. Activation of MAPKs regulates excessive proliferation and increased motility of ECs and SMCs, and selectively inhibiting MAPKs can reverse this abnormal behavior (Fig. 4a).

### p38 promotes endothelial apoptosis in PH

The pro-and anti-apoptotic effects exhibited by p38 on PAECs in PAH may be achieved by the activation of different isoforms. The p38 α isoforms and the β isoforms have apoptotic and survival effects, respectively. The pro-apoptotic effect of p38 may have a beneficial role in PAH by helping to alleviate disease progression. In the ECs of patients with IPAH, the expression of Ano1 is compensatorily increased.^151^ In rat PAECs, activated Ano1 stimulates the phosphorylation of p38 by increasing mitochondrial ROS, thereby activating Caspase-3 to promote apoptosis.^151^

### MAPK promotes cell proliferation in PH

In patients with PAH, abnormal activation of the MAPK pathway leads to excessive proliferation and disrupted apoptosis in PASMCs and PAECs, contributing to pulmonary vascular remodeling. While all three major MAPK pathways promote cell proliferation, they have a dual role in regulating apoptosis.

#### ERK

The levels of growth factors (including VEGF, PDGF, TGF-β) and ET-1 were increased in PAH patients,^152,153^ promoting cell proliferation by activating ERK. In a murine PAH model with IL-6 over-expression, increased VEGF induced increased ERK activation in PASMCs, which subsequently promoted c-Myc generation and increased anti-apoptotic proteins survivin and Bcl-2, forming a proliferative and anti-apoptotic state.^154^ In PDGF-induced human PASMCs, the ERK pathway is activated to induce an increased expression of Egr-1, which directly binds the SphK1 promoter and promotes its expression and subsequent cell proliferation.^155^ Activation of tyrosine kinase receptors by PDGF or FGF2 in rat PASMCs leads to the downregulation of atrial natriuretic peptide clearance receptor expression via the MEK/ERK signaling pathway. This, in turn, indirectly contributes to cell proliferation in hypoxia-PH.^156^ In human PASMCs, ET-1 binds to the ETA receptor, which activates ERK1/2 through the Ras/Raf/MEK pathway, ultimately enhancing cell proliferation by increasing c-Fos expression.^157^ In 5-HT-induced PASMCs, ERK pathway activation mediates its inhibition of PPARγ expression, thereby exerting pro-proliferative and anti-apoptotic effects.^158^ In PASMCs and PDGF-stimulated PASMCs from PAH patients, PFKFB3 is upregulated, triggering the ERK1/2 pathway through glycolysis-generated lactate, which subsequently activates Calpain-2 and promotes cell proliferation.^159^ However, the inhibition of ERK1/2 using PD98059 simultaneously reduced the apoptosis of the PASMCs from hypoxic rats.^159^ This suggests that the ERK pathway may have both anti-and pro-apoptotic effects in PH.

#### p38

Abnormal activation of p38 promotes the development of PAH by stimulating the proliferation of PAECs and PASMCs. However, at the same time, p38 can play a pro-apoptotic role in PASMCs, potentially contributing positively through this pathway. Inflammation associated with PAH attracts CD8^+^ T cells, which secrete GzmB that cleaves intersectin-1s, producing the NH2-terminal fragment EH_intersectin-1s_ with proliferative potential in ECs.^160,161^ EH_intersectin-1s_ activates the p38/Elk-1 pathway, leading to the upregulation of c-Fos and promoting the proliferation of PAECs.^160,162^ Reduced expression of DUSP1 in PASMCs of PAH patients, along with an impaired p38/MSK1/DUSP1 negative feedback loop, leads to increased and uncontrolled cell proliferation.^163^ The pro-apoptotic p38 pathway is inhibited in a murine PAH model of IL-6 over-expression.^154^ In a rat PAH model induced by MCT, over-expression of miR-15a-5p inhibited VEGF, leading to the phosphorylation of p38 and an increase in MMP2 levels. This resulted in elevated activity of Caspase-3 and Caspase-9, increased expression of the pro-apoptotic protein Bax, decreased expression of the anti-apoptotic protein Bcl-2, and ultimately promoted apoptosis in PASMCs.^164^

#### JNK

JNK mainly plays a pro-proliferative and anti-apoptotic role in PAH. Ma et al. demonstrated through in vitro experiments that epoxyeicosatrienoic acids, activates the c-Jun pathway by stimulating JNK and facilitating its translocation to the nucleus.^165^ This process leads to the upregulation of cell cycle-related proteins, promoting the transition from the G0/G1 phase to the S phase, while also inhibiting Caspase-3 activity, consequently, playing a pro-proliferative and anti-apoptotic role.^165^ However, in PAH mice that over-express IL-6, JNK was inhibited as a pro-apoptotic factor, indicating that JNK has a dual role in regulating apoptosis in PAH.^154^

### MAPK promotes ECM remodeling in PH

The activated MAPK pathway observed in PAH regulates ECM remodeling in the pulmonary vasculature and right ventricle by promoting changes in fibroblast phenotype and enhancing the synthesis and secretion of ECM proteins.

#### ERK

ERK plays a role in ECM remodeling in PAH patients by promoting the synthesis of COL1, as well as the proliferation and migration of fibroblasts. In experiments involving PASMCs and PDGF-induced PASMCs from PAH patients, the activated ERK pathway enhances COL1 protein synthesis by activating Calpain-2, which subsequently coordinates ECM deposition.^159^

#### p38

In PAFs of PAH models, the activated p38 pathway promotes ECM remodeling by promoting cell proliferation, activation, migration, and inducing collagen synthesis. ASK1 is a member of the MAPK kinase family and is required for sustained JNK/p38 activation and apoptosis induced by TNF and oxidative stress.^166^ ASK1 activity, protein levels, and phosphorylation were increased in PAFs of IPAH patients, and ASK1 promoted pathological remodeling of pulmonary vessels and right ventricle in rat MCT- and Su/Hx-PAH models.^167^ ASK1 promoted the activation/migration of PAFs in the RVFs of pulmonary artery banding mice and IPAH patients through the activation of JNK/p38.^167^ Hypoxia activates the p38 pathway in rat PAFs, which in turn promotes a proliferative phenotype in fibroblasts and triggers the release of IL-6, which stimulates SMC proliferation via the STAT3 pathway, thereby promoting pulmonary vascular remodeling, with ASK1 potentially involved in this process.^168^ This indicates that p38 activation plays a role in inflammation and vascular remodeling through paracrine signaling of pro-inflammatory cytokines and chemokines.

### MAPK promotes mitochondria dysfunction and metabolic reprogramming in PH

#### p38

p38 can be activated by oxidative stress, contributing to the development of PAH by increasing ROS levels. Additionally, in hypoxia-induced PAECs and MCT-PAH rats, activation of the p38 pathway increases Δψm and hyper-polarization by elevating MK2 expression, resulting in increased levels of mitochondrial ROS.^169^

### MAPK promotes inflammation in PH

The activated MAPK pathway plays a crucial role in the inflammatory response in PAH by regulating the expression of inflammatory factors in PAECs and PASMCs, as well as addressing immune cell abnormalities. Additionally, there is a positive feedback loop in which the MAPK pathway enhances the production of inflammatory factors.

#### JNK

Phosphorylation of the JNK pathway plays a role in macrophage polarization, which is significant in pulmonary vascular remodeling associated with PAH. An imbalance between M1 and M2 macrophages has been noted in monocyte-derived macrophages from PAH patients.^170^ Previous research indicated that JNK phosphorylation promotes M1 polarization while inhibiting M2 polarization in macrophages.^171^ However, Zhang et al. discovered that tumor M2 macrophages over-expressing NOX4 exhibited increased JNK activity, leading to the expression and release of HB-EGF, which promotes non-small cell lung cancer proliferation in vitro.^172^ This suggests that the regulatory role of JNK in macrophage activation may also be relevant in the context of PAH.

### MAPK promotes EndMT in PH

In PAH, activated MAPK pathways facilitate EndMT by regulating downstream molecules like Snail and VCAM-1. Kong et al. observed that the phosphorylation levels of ERK1/2, JNK, and p38 were differentially elevated in hypoxia induced PAECs, contributing to the process of EndMT.^173^

## PI3K/Akt signaling pathway in PH

In 2001, Yamboliev et al. first identified that the PI3K signaling pathway mediated PDGF-induced spreading and migration of canine PASMCs.^174^ Activated Akt is the central molecule in PI3K signaling pathway, which promotes the proliferation and migration of human PASMCs after PDGF treatment.^174^ PI3K/Akt signaling pathway is also activated in the pulmonary arteries of patients with COPD-PH, CTEPH,^175^ and PAH,^176^ and animal models of hypoxia-PH,^177,178^ and MCT-PAH,^39,176^ to regulate inflammation, proliferation, mitochondrial dysfunction, oxidative stress, apoptosis, and metabolic dysregulation in the context of PH development (Fig. 4b). This occurs through downstream molecules such as eNOS,^39,177,178^ mTOR,^179,180^ GSK3β,^176,181^ STAT3, FOXO3,^182^ Smad1/5/8,^183^ Lipoxygenase, NO,^184^ PGI,^184^ and ET-1.^184^

### PI3K/Akt suppression in the early stage of PAH drives endothelial apoptosis and dysfunction

At the early stage of PAH, PAECs undergo apoptosis due to the inhibition of the PI3K/Akt pathway, leading to endothelial dysfunction. This phenomenon is supported by several studies, which demonstrate that activating the PI3K/Akt pathway can alleviate PH through the enhancement of eNOS signaling, promoting cell survival and restoring endothelial function.^185,186^

### PI3K/Akt excessive activation in the progressive stage of PH suppresses apoptosis and drives cell proliferation

As the disease progresses, compensatory mechanisms trigger excessive PI3K/Akt signaling, which exacerbates PH by promoting the over-proliferation of PAECs and PASMCs. Thus, studies have demonstrated that the PI3K/Akt pathway activation can trigger down signaling molecules including mTOR,^187^ FOXO3a,^182^ and Smad1/5/8^183^ to suppress apoptosis, thereby favoring the proliferation of endothelial and SMCs, subsequently promoting the development of PH. A growing body of evidence suggests that BMP4 plays a critical role in pulmonary fibrosis and vascular remodeling, impacting processes such as proliferation, cell migration, and apoptosis.^188^ However, it is important to note that BMP4 has been found to have different effects depending on the location of the pulmonary artery. In PASMCs isolated from the proximal pulmonary arteries, BMP4 inhibits proliferation, whereas in PASMCs from peripheral pulmonary arteries, it stimulates proliferation.^188,189^ Moreover, upregulation of miR-19a, miR-371b-5p, and METTL3 in PAH inhibit cell apoptosis by suppressing phosphatase and tension homolog, a key inhibitor of the PI3K/Akt pathway, in PASMCs and PAECs.^190,191^

### PI3K/Akt signaling enhances mitochondria dysfunction and metabolic reprogramming in PH

In the progression of PH, the occurrence of oxidative stress is evident, particularly with the activation of the PI3K/Akt/eNOS/NO signaling pathway in cells such as PAECs.^177^ This pathological process has implications for mitochondrial function and metabolism, serving as fundamental features in the development of PH. eNOS, belonging to the NOS enzyme family encoded by *Nos2*, facilitates the transformation of L-arginine into NO. Excessive production of NO can impact mitochondrial function, and influence processes such as oxidative phosphorylation. NO can reduce oxidative phosphorylation by directly inhibiting cytochrome c oxidase in the mitochondrial respiratory chain. This interference disrupts the normal flow of electrons, leading to a decrease in ATP production through oxidative phosphorylation which is crucial to PH development.^192^ Moreover, a study revealed that targeting glycolytic protein α-enolase might reduce experimental hypoxia-PH by improving endothelial and mitochondrial dysfunction via the PI3K-Akt-mTOR signaling pathway.^193^ mTOR, which exists in two distinct complexes, mTORC1, and mTORC2, each with unique functions, plays a contributory role in PH by mediating PI3K/Akt signaling in influencing mitochondria function and metabolism in endothelial and SMCs.^194,195^

### PI3K/Akt suppression in the early stage of PAH promotes endothelial inflammation and dysfunction

Numerous studies have demonstrated that CTRP9 has anti-inflammatory properties and modulates inflammatory responses, thereby attenuating EC dysfunction in PAH.^196,197^ Over-expression of CTRP9 significantly mitigates inflammation, apoptosis, and ECM accumulation in PAH by activating the Akt pathway.^198^

## NF-κB signaling pathway in PH

**Fig. 5:**
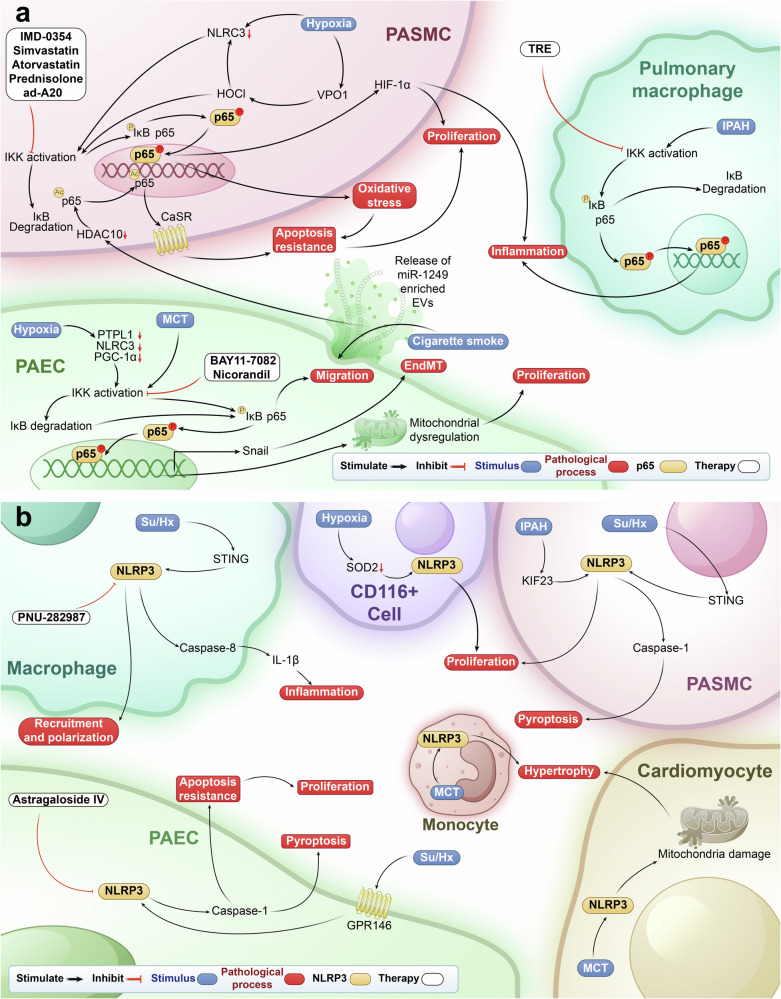
NF-κB and NLRP3 signaling pathways in PH. **a** NF-κB signaling pathway and targeted therapy in PH. PDGF, hypoxia, MCT and cigarette smoke activate NF-κB in PASMCs and PAECs, promoting proliferation, resistance to apoptosis, and inflammation via p65 nuclear translocation, HIF-1α activation, and CaSR upregulation. In hypoxia-PH, NLRC3 and PGC-1α are reduced, leading to activation of p65 in PAECs, which drives apoptosis, migration, and EndMT. In IPAH, NF-κB is activated in macrophages, promoting inflammation. NF-κB inhibition can alleviate PH using agents like IMD-0354, Simvastatin, Atorvastatin, Ruscogenin, Prednisolone, ad-A20, BAY11-7082, Nicorandil, and TRE. *IKK* IκB kinase, *IκB* inhibitor of NF-κB, *HIF-1α* hypoxia-inducible factor 1-alpha, *EV* extracellular vesicle, *HDAC10* histone deacetylase 10, *CaSR* calcium-sensing receptor, *NLRC3* NLR family CARD domain-containing protein 3, *PGC-1α* peroxisome proliferator-activated receptor gamma coactivator-1 alpha, *EndMT* endothelial-to-mesenchymal transition, *IPAH* idiopathic pulmonary arterial hypertension, *NF-κB* nuclear factor kappa B, *PASMC* pulmonary artery smooth muscle cell, *PAEC* pulmonary artery endothelial cell, *TRE* triterpenoid, *TGF-β* transforming growth factor-β, *VPO1* vascular peroxidase 1, *PTPL1* protein tyrosine phosphatase L1, *HOCl* hypochlorous acid, *MCT* monocrotaline. **b** NLRP3 signaling pathway and targeted therapy in PH. Su/Hx-PAH activates NLRP3 in PAECs, causing proliferation, apoptosis resistance, and pyroptosis. In macrophages, this trigger activates NLRP3, promoting inflammation and IL-1β release. In IPAH and Su/Hx-PAH, NLRP3 is activated in PASMCs, inducing pyroptosis and proliferation. In MCT-PH, NLRP3 activation in monocytes and cardiomyocytes drives hypertrophy and mitochondrial dysfunction. NLRP3 activation contributes to PH progression. PNU-282987 and Astragaloside IV inhibit NLRP3 in macrophages and PAECs. *PAEC* pulmonary artery endothelial cell, *ROS* reactive oxygen species, *NLRP3* NLR family pyrin domain containing 3, *Su/Hx-PAH* SU5416-hypoxia-induced pulmonary arterial hypertension, *GPR146* G-protein coupled receptor 146, *MCT* monocrotaline, *HMGB1* high-mobility group box 1, *STING* stimulator of interferon genes, *IL-1β* interleukin-1 beta, *SOD2* superoxide dismutase 2, *IPAH* idiopathic pulmonary arterial hypertension, *MCT-PH* monocrotaline-induced pulmonary hypertension, *PNU-282987* a selective α7-nicotinic acetylcholine receptor agonist, *Astragaloside IV* an active compound from Astragalus membranaceus

NF-κB is a transcription factor that regulates the expression of genes involved in inflammation, immune response, cell survival, and proliferation.^199^ In its inactive state, NF-κB is bound to inhibitory proteins (IκB), which are subsequently degraded by the IKK complex upon activation. This releases active NF-κB, allowing it to translocate into the nucleus and regulate the transcription of genes involved in inflammation, immune responses, cell survival, and proliferation. Various stressors, including hypoxia, inflammatory signals, and stimuli associated with PH, have been identified as triggers for the activation of NF-κB.^200^ Afterward, several studies have demonstrated the significant involvement of the NF-κB pathway in various cellular processes linked to the development of PH (Fig. 5a).^201–203^ According to Kimura et al. increased activity of NF-κB was observed in small pulmonary arterial lesions and alveolar macrophages in lungs of patients with PAH compared with lungs of control patients.^204^ A single-cell analysis of two rat models exhibiting PAH highlights robust activation of the NF-κB pathway in rats subjected to MCT and Su/Hx, compared to control rats.^202^

### NF-κB promotes anti-apoptosis and cell proliferation in PH

Preclinical studies involving both in vitro and in vivo settings show that hypoxia-induced proliferation of PASMCs occurs through the IKK/NF-κB p65 pathway.^205^ This cascade further enhances vascular remodeling and contributes to the progression of PH.^205^ ROS are integral to the process of pulmonary vascular remodeling linked with hypoxia-PH. VPO1, identified as a recently discovered haeme-containing peroxidase, expedites the development of oxidative stress in the vasculature. In the context of vascular remodeling associated with hypoxia-PH, You et al. illustrated that VPO1 facilitates hypoxia-induced proliferation, resistance to apoptosis, and migration in PASMCs through VPO1/hypochlorous acid/NF-κB signaling pathway.^206^ A study demonstrated that cigarette smoke increases plasma endothelial EVs and stimulates their release from PAECs. MiR-1249 was found to be predominantly and highly expressed in these endothelial EVs from cigarette smoke-exposed rats and humans, and this was confirmed in endothelial EVs from cigarette smoke extract-treated PAECs, but not in those from cigarette smoke extract-treated PASMCs. MiR-1249 downregulates HDAC-10, leading to increased levels of acetylated NF-κB and elevated CaSR expression.^201^ This signaling cascade, involving the activation of CaSR expression enhances the proliferative and anti-apoptotic properties of PASMCs and contributes to PH development.^201,207–210^ Repressing miR-1249 or manipulating its pathway significantly reduces SMC proliferation and alleviates PH.^201^ In diverse PH models, including hypoxia-PH, Su/Hx-PAH, and MCT-PAH, blocking the NF-κB pathway has been shown to restore the equilibrium between proliferation and apoptosis in PASMCs, alleviating vascular remodeling.^211,212^

### NF-κB promotes mitochondria dysfunction and metabolic reprogramming in PH

PGC-1α serves as a crucial controller of cellular energy metabolism and a master regulator of mitochondrial biogenesis. In the in vitro and in vivo experimental hypoxia-PH models, PGC-1α hampers oxidative metabolism and mitochondrial function, driven by elevated ROS formation, mitochondrial swelling, and activation of NF-κB.^213^

### NF-κB promotes inflammation in PAH

Preclinical investigations using models of PAH induced by MCT and hypoxia have linked the activation of the NF-κB pathway to elevated levels of IL-1β, IL-6, and TNF in both serum and lung tissues.^214^ For instance, at the animal level, a study utilizing an MCT-PAH model in rats demonstrated an increase in the relative expression levels NF-κB p65 in the lungs. This elevation was correlated with heightened levels of inflammatory markers in the serum, including IL-6, TNF, ICAM-1, and HMGB1.^215^

### NF-κB promotes EndMT in PAH

In a study involving rats with MCT-PAH, it was shown that the increased expression of EndMT-associated molecules, including N-cadherin, vimentin, Snail, and Slug, was partially attributed to the activation of the NF-κB pathway.^216^ In the lungs of MCT-PAH rats and during TGF-β1-induced EndMT in human PAECs, the level of phosphorylated IκBα increases while the overall IκBα content decreases. This leads to the activation of NF-κB, an increase in p65 DNA-binding activity, and subsequent upregulation of Snail transcription and expression. Additionally, hypoxia or IL-1β can suppress PTPL1 in vascular ECs. The downregulation of PTPL1 promotes NF-κB signaling by preventing the dephosphorylation of IκBα at tyrosine 42, thereby extending the half-life of IκBα. This process further increases Snail levels and drives EnMT.^217^

## NLRP3 signaling pathway in PH

The NLRP3 signaling pathway has emerged as a critical player in the pathogenesis of PH. Activation of the NLRP3 inflammasome, a multiprotein complex involving NLRP3, apoptosis-associated speck-like protein containing a domain-containing protein 3, and pro-Caspase-1, leads to the cleavage of Caspase-1 and subsequent release of pro-inflammatory cytokines, notably IL-1β and IL-18. Villegas et al. in 2013 were the first to demonstrate NLRP3 inflammasome activation, involving Caspase-1 cleavage, and the release of active IL-1β and IL-18 in chronic hypoxia-PH, along with its attenuation by the SOD mimetic, MnTE-2-PyP.^218^ In both patients with PAH and preclinical models of MCT-PAH, a study revealed the activation of NLRP3-macrophages in the decompensated right ventricle, and the enhancement of RV function was observed upon the inhibition of NLRP3 signaling.^13^ Moreover, research has implicated the activation of NLRP3 in influencing processes, including inflammatory responses, pyroptosis, and apoptosis, contributing to the pathogenesis of PH (Fig. 5b).^13,219^

### NLRP3 promotes anti-apoptosis and cell proliferation in PH

In a Su/Hx-PAH mouse model and MCT-PAH, increased proliferation of PASMCs was closely associated with activation of the NLRP3.^220^ SOD2, an essential antioxidant enzyme responsible for neutralizing superoxide radicals, plays a crucial role in PH with the implication of the NLRP3 pathway.^221^ In PH, the involvement of SOD2 extends beyond its antioxidant function, as studies have suggested its association with apoptosis and proliferation.^221^ SOD2, by regulating oxidative stress, can influence apoptotic pathways, potentially impacting cell survival and death mechanisms in pulmonary vascular cells. Furthermore, alterations in SOD2 expression may contribute to dysregulated cell proliferation observed in PH, influencing vascular remodeling and hypertrophic responses.^221^ A study found that SOD2 was downregulated in the obstructive sleep apnea/chronic intermittent hypoxia model, and this deficiency intensified chronic intermittent hypoxia-PH and pulmonary vascular hypertrophy.^221^ Under chronic intermittent hypoxia conditions, CD11b^+^ cells, particularly monocytic myeloid cells of the Ly6C^+^Ly6G^-^ subtype, exhibited elevated levels in the lung, bone marrow, and blood. The downregulation of SOD2 activated the NLRP3 inflammasome specifically in CD11b^+^ cells. In turn, SOD2-deficient CD11b^+^ myeloid cells were found to enhance apoptosis resistance and promote the over-proliferation of human PASMCs by upregulating NLRP3.^221^ Additionally, STING has been shown to affect the expression of the NLRP3 inflammasome, promoting the proliferation of PASMCs during Su/Hx-PAH.^8^

### NLRP3 promotes mitochondria dysfunction and metabolic reprogramming in PAH

Mitochondrial dysfunction and metabolic alterations are pivotal aspects in the pathophysiology of PAH, contributing to the progression of vascular remodeling and cardiac dysfunction.^222^ In a study highlighted within this broader context, monocytes cultured with MCT, a model of PAH induction, demonstrated the activation of NLRP3 inflammasomes.^13^ This activation initiated a cascade of events that resulted in cardiomyocyte (CM) mitochondrial damage, as observed in coculture experiments.^13^ Suppression of NLRP3 inflammasome with the administration of MCC950, enhanced RV function in MCT-treated rats while reducing monocyte-induced hypertrophy and mitigating mitochondrial damage in normal CMs in vitro.^13^ The interaction between monocytes and CMs underscores the intricate interplay between immune cells and the cardiovascular system in the context of PH. The involvement of NLRP3 in this process suggests a link between inflammatory responses and mitochondrial dysfunction, adding a layer of complexity to the multifactorial nature of PH pathogenesis.

### NLRP3 promotes inflammation in PH

Within the context of PH pathogenesis, macrophages play a pivotal role in orchestrating inflammatory responses, and their interaction with the NLRP3 inflammasome further amplifies the inflammatory environment associated with PH. In individuals with PAH experiencing decompensated right ventricles, the pathway involving macrophages and NLRP3 exhibited increased upregulation.^13^ NLRP3, collaborating with macrophages in the canonical inflammasome pathway, assumes a pivotal role in regulating inflammatory responses, with implications for conditions such as PH. Using a Su/Hx-PAH mouse model and an MCT-PAH rat model, it was discovered that the pathogenic involvement of macrophages in pulmonary perivascular inflammation is contingent on macrophage-derived IL-1β through a Caspase-8-dependent canonical inflammasome pathway.^220^ STING pathway serves as a crucial link in inflammatory reactions, promoting the upregulation of NLRP3.^8^ In a study focused on Su/Hx-PAH, it was observed that STING activation played a significant role by upregulating the expression of NLRP3 and enhancing the activation of the macrophage NLRP3 inflammasome. The concerted action of STING and NLRP3 in macrophages contributed to an intensified inflammatory response and enhanced vascular proliferation in rats with Su/Hx-PAH.^8^ Furthermore, several studies have demonstrated that directing interventions towards the NLRP3 signaling pathway holds promise in alleviating inflammation, leading to the attenuation of PH.^223–225^

### NLRP3 promotes pyroptosis in PAH

Pyroptosis, characterized by the maturation and release of inflammatory mediators during cell death, has been demonstrated to be a pivotal factor in the development of PH, facilitated by the NLRP3 signaling pathway. GPR146 is a G-protein coupled receptor involved in various physiological processes, including pyroptosis by transducing signals across the cell membrane.^226^ Jiang et al. through RNA sequencing, discovered a substantial 11.64-fold increase in GPR146 expression in the Su/Hx-PAH model compared to controls. Moreover, heightened GPR146 expression was observed in human lung tissues with PAH and in lung tissues from Su/Hx-PAH rats. The study proposed that GPR146 induces pyroptosis by activating the NLRP3/Caspase-1 signaling axis, ultimately leading to endothelial injury and vascular remodeling.^227^

### NLRP3 promotes PAEC ferroptosis in PAH

Ferroptosis, an apoptotic-independent mode of cell demise activated through iron-dependent lipid peroxidation, plays a pivotal role in the development of various inflammation-associated diseases.^228^ Its involvement in PH remains a subject of exploration. In in vitro and in vivo settings of the MCT-PAH rat model, ferroptosis was detected in PAECs, marked by diminished cell viability, elevated labile iron pool levels, heightened lipid peroxidation, increased expression of NOX4 and reduced expression of GPX4 and FTH1.^219^

## NOTCH signaling pathway in PH

**Fig. 6:**
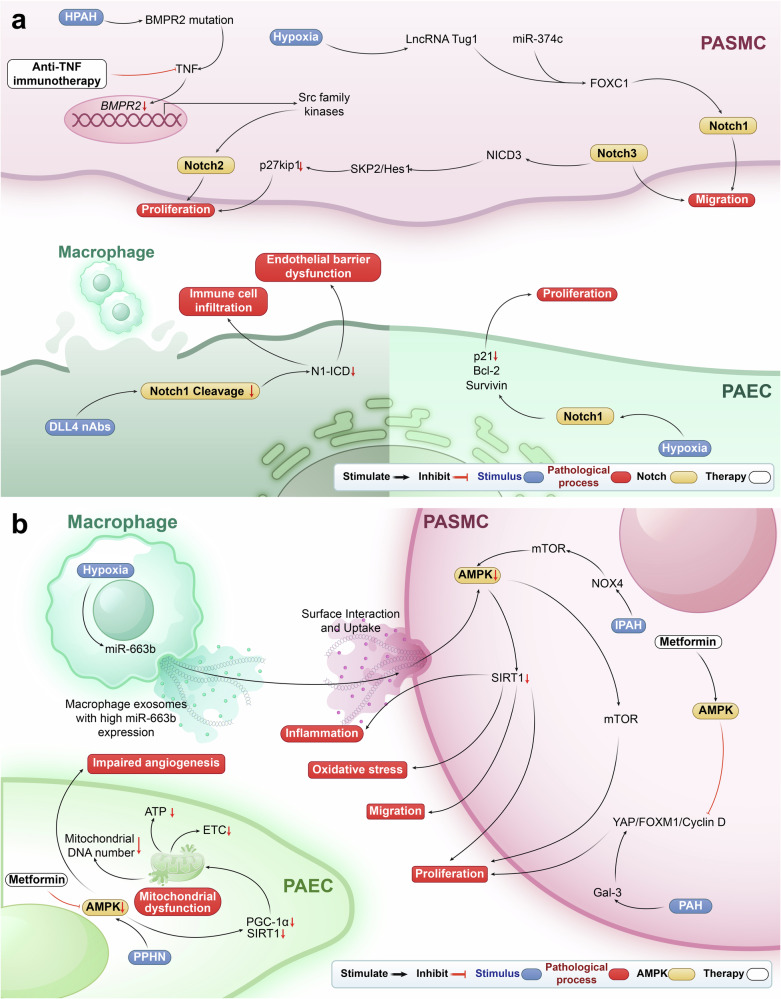
Notch and AMPK signaling pathways in PH. **a** Notch signaling pathway and targeted therapy in PH. *BMPR2* mutations in PASMCs increase TNF, reducing BMPR2 and activating Notch2. Hypoxia induces the expression of lncRNA Tug1, which activates Notch1 signaling, thereby promoting the migration of PASMCs. Notch3 activation leads PASMC migration. Additionally, it can cause the release of NICD3, which downregulates p27kip1, further enhancing PASMC proliferation. In PAECs, DLL4 nAbs impair barrier function, and hypoxia activates Notch1 to promote proliferation. Notch signaling contributes to PH, with anti-TNF and Propylthiouracil as potential therapies. *PASMC* pulmonary artery smooth muscle cell, *BMPR2* bone morphogenetic protein receptor type 2, *TNF* tumor necrosis factor, *miR* microRNA, *PAH* pulmonary arterial hypertension, *Notch1/2/3* Notch receptors 1/2/3, *FOXC1* forkhead box C1, *NICD* Notch intracellular domain, *SKP2* S-phase kinase-associated protein 2, *Hes1* hairy and enhancer of split 1, *p27kip1* cyclin-dependent kinase inhibitor 1B, *PAEC* pulmonary artery endothelial cell, *DLL4* Delta-like ligand 4, *N1-ICD* Notch1 intracellular domain, *p21* cyclin-dependent kinase inhibitor 1A, *Bcl-2* B-cell lymphoma 2, *survivin* Baculoviral IAP repeat-containing 5, *HPAH* heritable pulmonary arterial hypertension, *lncRNA* long non-coding RNA, *Tug1* taurine-upregulated gene 1. **b** AMPK signaling pathway and targeted therapy in PH. In hypoxia-PH, miR-663b is upregulated in macrophages, releasing exosomes that suppress AMPK in PASMCs, driving inflammation, oxidative stress, and proliferation. In IPAH PASMCs, NOX4 activates mTOR, inhibiting AMPK and promoting proliferation. In PAECs, suppressed AMPK in PPHN impairs angiogenesis and mitochondrial function. Metformin upregulate AMPK, mitigating PH progression. *AMPK* AMP-activated protein kinase, *PH* pulmonary hypertension, *PASMC* pulmonary artery smooth muscle cell, *PAEC* pulmonary artery endothelial cell, *miR* microRNA, *IPAH* idiopathic pulmonary arterial hypertension, *NOX4* NADPH oxidase 4, *mTOR* mechanistic target of rapamycin, *SIRT1* sirtuin 1, *YAP* Yes-associated protein, *FOXM1* forkhead box M1, *Cyclin D* cyclin D1, *Gal-3* galectin-3, *PGC-1α* peroxisome proliferator-activated receptor gamma coactivator 1-alpha, *ETC* electron transport chain, *PPHN* persistent pulmonary hypertension of the newborn, *ATP* adenosine triphosphate, *PAH* pulmonary arterial hypertension

In 2009, Li et al. initially discovered the significance of the Notch3-Hes-5 signaling pathway in the progression of PAH.^229^ The Notch signaling pathway, particularly through Notch1, Notch2, Notch3, and Notch4, plays a pivotal role in PH development.^229–231^ Located predominantly within ECs, Notch1, and Notch2 are closely tied to fostering cell proliferation and ensuring survival, critical elements in the initiation of PAH.^231,232^ Notch3 is expressed in arterial SMCs, influencing their behavior and phenotype,^233^ while Notch4 is highly expressed in the pulmonary vasculature media, upregulated in hypoxia-PH, and promotes SMC proliferation and migration.^230^ In patients with IPAH and PAH, heightened levels of Notch1 and Notch2 expression were observed in the lungs when compared to those in healthy individuals.^231,232^ Li et al. observed heightened Notch3 expression in small PASMCs, establishing a direct correlation between the severity of PH in both humans and rodents and the expression of Notch3 in the lungs.^229^ Moreover, Guo et al. show that there is a significant upregulation of Notch4 expression in the media of pulmonary vasculature, both in lung tissues from individuals with hypoxia-PH and in hypoxia-PH rats, when compared to control groups.^230^ The Notch signaling cascade commences with ligand-receptor interactions, initiating the cleavage of the Notch receptor and subsequent release of the intracellular domain. Upon activation of Notch signaling, the Notch intracellular domain is liberated. This Notch intracellular domain then translocates to the nucleus, where it forms a complex with CSL and Mastermind-like, regulating target gene expression and influencing critical cellular processes such as inflammation, apoptosis, and proliferation, which are crucial to the pathogenesis of PH as demonstrated by several clinical and preclinical studies (Fig. 6a).^230–232^

### Notch signaling enhances anti-apoptosis and proliferation in PH

The Notch signaling pathway has been shown to play a pivotal role in apoptosis and proliferation processes in PH.^232^ In vitro, studies manipulating Notch1 function revealed its capacity to enhance human PAECs proliferation through p21 downregulation while inhibiting apoptosis via Bcl-2 and Survivin during PH.^232^ Emerging evidence suggests that long non-coding RNAs (lncRNAs) and miRNAs may modulate the Notch signaling to influence key processes in PH, including apoptosis and proliferation. For instance, the inhibition of lncRNA Tug1 in hypoxia-PH has been shown to suppress FOXC1 through miR-374c binding, resulting in decreased proliferation and migration while enhancing apoptosis in PASMCs, ultimately impeding pulmonary vascular remodeling by modulating the Notch signaling pathway.^234^ In humans, there are three main types of Jagged proteins: Jagged-1, Jagged-2, and Jagged-3. These proteins serve as ligands for the Notch receptors and are integral transmembrane proteins. The transmembrane nature of Jagged proteins is essential for their role in initiating the Notch signaling. Upon cleavage, the resulting soluble Jagged engages with Notch receptors, instigating a signaling cascade that impacts diverse cellular processes, including proliferation. Notably, Jagged-1 has been demonstrated to play a crucial in modulating the pathogenesis of PH.^235,236^ Xiao et al. demonstrated that soluble Jagged-1 exhibits potential as a therapeutic intervention. It restricts the proliferation of PASMCs and restores the phenotype of PH-associated PASMCs, transitioning them from a dedifferentiated to a differentiated state by intervening in the Notch-HEY2 pathway.^235^ According to Zhang et al. Notch3 signaling activation stimulated pulmonary vascular cell proliferation by SKP2-and Hes1-mediated p27Kip1 reduction during MCT-PAH.^237^ Heightened expression and transcriptional activity of Notch1 in human PAECs have been demonstrated under hypoxic conditions, contributing to hypoxia-induced cell proliferation. Notably, the targeted knockdown of *Notch1* effectively impedes this proliferative response.^232^

### Notch signaling regulates inflammation in PH

Heterozygous germ-line mutations in the *BMPR2* gene underlie HPAH. In HPAH, heterozygous *BMPR2* mutations induce inflammation, as TNF selectively reduces *BMPR2* transcription and activates Notch2 signaling via Src family kinases. Anti-TNF immunotherapy reverses disease progression, highlighting a potential therapeutic strategy for restoring normal BMP/Notch signaling and mitigating PAH.^238^ In PH, neointima formation involves a Notch3-marked subset of SMCs and inhibition of the Notch signaling has been shown to improve inflammation-driven mPAP.^239^ However, it is crucial to recognize that distinct effects arise when inhibiting different components of the Notch pathway in PH. For instance, inhibiting Notch1 cleavage with Delta-like 4 neutralizing antibodies induces PH by compromising lung endothelial barrier function, promoting immune cell infiltration. This, in turn, contributes to elevated RV pressure and remodeling.^240^ This underscores the intricate balance within the Notch pathway, where inhibiting the receptor and its cleavage, though conceptually similar, yield distinct effects in the context of PH.

## AMPK signaling pathway in PH

AMPK serves as a pivotal regulator of cellular energy balance.^241^ Comprising α, β, and γ subunits, AMPK is activated in response to a heightened AMP to ATP ratio, a hallmark of low cellular energy. AMPK activation involves the binding of AMP to the γ subunit, allosterically promoting phosphorylation at Thr172 in the α subunit activation loop. This process, facilitated by upstream kinases like LKB1 and CaMKK, activates AMPK, which subsequently phosphorylates target involved proteins in metabolic pathways. The β subunit stabilizes the heterotrimeric complex, emphasizing the role of AMPK as a central energy sensor orchestrating cellular responses to maintain energy homeostasis.^15^ AMPK is ubiquitously expressed and its activation in certain conditions exerts multifaceted effects on cellular processes, including anti-inflammatory actions, anti-apoptotic effects, and the regulation of cell proliferation, promoting angiogenesis, facilitating new blood vessel formation, and enhancing NO bioavailability, contributing to improved vascular function and cardiovascular health.^242^ In 2011, Chandra et al. were pioneers in linking AMPK to PH, demonstrating that disruption of the APLN-APJ pathway worsens hypoxia-PH via reduced AMPK activation.^243^ Following this, numerous research contend that AMPK activation might offer protective effects in PH, fostering vasodilation, restraining vascular remodeling, and alleviating inflammation.^242,244^ On the contrary, few studies suggest that the activation of AMPKα1 in the early stages of PH may contribute to disease pathogenesis.^245,246^ Clinical research has demonstrated a correlation between the stimulation of the AMPK pathway and its potential application as a therapeutic approach for PAH.^244,247,248^ In diverse animal and cellular models of PH, such as the MCT-PAH, hypoxia-PH, Su/Hx-PAH, and persistent PH of the newborn (PPHN), investigations have shown that activating AMPK can alleviate aberrant cellular processes linked to the progression of PH (Fig. 6b).^244,249,250^

### AMPK regulates anti-apoptosis and cell proliferation in PH

In PAH, Gal-3 emerges as a promoter of PASMC proliferation. A study investigating the underlying mechanisms revealed that Gal-3 activates YAP/ FOXM1/Cyclin D signaling cascade, contributing to PASMC proliferation in PAH. Importantly, the activation of AMPK is identified as an inhibitory mechanism against Gal-3-induced PASMC proliferation by targeting YAP/FOXM1/Cyclin D pathway.^251^ The complex interplay between AMPK and the mechanistic target of rapamycin mTOR holds pivotal significance in processes associated with PH, influencing cellular proliferation, survival, and apoptosis regulation. A study indicated that both mTORC1 and mTORC2 are upregulated in IPAH. However, the activation of mTORC2 in IPAH is driven by increased levels of NOX4, which leads to the downregulation of the energy sensor AMPK. This downregulation results in the subsequent activation of the mTORC1-S6 pathway, promoting the proliferation and survival of PASMCs.^252^ These findings suggest that enhancing AMPK activity could mitigate the detrimental effects of mTOR signaling, offering a potential therapeutic benefit in PAH by reducing PASMC proliferation and promoting apoptosis.

In contrast to the aforementioned evidence, studies have intriguingly shown that hypoxia can rapidly activate AMPKα1 in PASMCs within 15 minutes to promote cell survival, and after ~30 min, AMPKα1 returns to baseline.^245,246^ Furthermore, another group found that under sustained hypoxic conditions (8 h), elevated levels of α-enolase in PASMCs lead to the activation of the AMPKα1-Akt signaling cascade, promoting PASMC proliferation, dedifferentiation, and resistance to apoptosis.^253^

### AMPK mitigates fibrosis and RVH in PAH

The AMPK activator, metformin has been shown to mitigate fibrosis in various organs including the lungs^254^ and the heart.^255^ For instance, a study demonstrated that metformin, attenuated pulmonary fibrosis by suppressing fibroblast proliferation through the downregulation of the transcription factor FOXM1.^254^ In another study, metformin was able to attenuate hyperhomocysteinemia-induced cardiac hypertrophy by decreasing myocardial fibrosis.^255^ Moreover, several studies have implicated myocardial fibrosis as a crucial factor characterizing RVH and dysfunction in PH.^256–258^ In a rat model of MCT-PAH, metformin has been shown to enhance eNOS activity, reduce pulmonary vascular remodeling, and inhibit fibrosis and RVH,^256,257^ suggesting the therapeutic potential of AMPK activation in mitigating fibrosis during the development of PH.

### Dual role of AMPK in mitochondrial dysfunction and metabolic reprogramming in PH

According to research, reduced AMPK function in PPHN led to mitochondrial dysfunction and impaired angiogenesis, suggesting a critical interplay between AMPK, mitochondria, and vascular development in PPHN.^250,259^ In PPHN, impaired vasodilation is associated with mitochondrial dysfunction, characterized by decreased mitochondrial DNA copy number, electron transport chain complex subunit levels, and ATP levels in PAECs and lung tissues. This dysfunction is linked to reduced threonine-172 phosphorylation of AMPK and decreased levels of PGC-1α and SIRT1.^259^ WNK1 is a serine/threonine kinase, a type of protein kinase, that plays a role in the regulation of ion transport in cells. The name “With No Lysine” refers to the unique amino acid sequence in the kinase domain of this protein. WNK1 is known to be involved in the control of electrolyte balance, blood pressure regulation, and cell volume homeostasis.^260^ In addition to its role, WNK1 is implicated in the regulation of cellular metabolism during the pathogenesis of PH. Inhibiting WNK1 in an MCT-PAH rat model has been demonstrated to activate AMPK, correcting metabolic dysregulation, and preserving mitochondrial enzyme levels, thereby improving both systolic and diastolic function in the right ventricle.^261^ Moreover, intermittent fasting has been shown to preserve RV function in PAH by activating AMPK to improve lipid metabolism and normalize mitochondrial and microtubule dynamics.^262^

However, despite the generally beneficial roles of AMPK in regulating energy homeostasis and metabolic function, research suggests that its activation may be maladaptive under specific pathological conditions in certain cells. For instance, in *BMPR2* mutant CMs, which are linked to heritable forms of PAH, AMPK is chronically hyper-activated, contributing to insulin resistance and lipotoxicity in the right ventricle. Although AMPK activation typically enhances fatty acid oxidation and mitochondrial function, in this case, it paradoxically reduces metabolic plasticity, impairs glucose metabolism, and promotes lipid accumulation. This altered metabolic profile is primarily coupled with increased MFGE8-driven signaling.^263^ This evidence highlights that, while AMPK is a key regulator of cellular metabolism, its effects may be context-dependent, and in certain disease states like HPAH, excessive or prolonged activation can exacerbate metabolic dysfunction and contribute to disease progression.

### AMPK suppression in PH exacerbates inflammation

Metformin, renowned for its role as an AMPK activator in diabetes management, is now gaining attention as a promising treatment for PH. Its potential lies in addressing key pathological processes linked to PH development, such as inflammation, while simultaneously enhancing endothelial function and mitigating vascular remodeling.^256,257^ Endothelial AMPK downregulation in PAH patients and hypoxia-PH mice has been shown to accelerate the disease progression.^244^ Omura et al. revealed that targeting AMPK with metformin attenuates PH by reducing inflammation, emphasizing AMPK as a novel therapeutic target for PAH treatment. Moreover, in an experimental rat model of mild PH induced by MCT, Remiszewski et al. showed that metformin, partially alleviated effects associated with PH. This involved a decrease in RVSP, hypertrophy, and inflammatory responses.^257^ In PH, upregulated miR-663b in hypoxia-induced PASMCs and M1 macrophages have been associated with increased inflammation and oxidative stress in PASMCs.^264^ A study revealed that miR-663b targeted AMPK, inhibiting the AMPK/SIRT1 pathway. Activation of AMPK mitigated the detrimental effects of miR-663b over-expression and M1 macrophage exosomes on PASMCs. In vivo, M1 macrophage exosomes with low miR-663b expression attenuated pulmonary vascular remodeling in PH rats, suggesting that exosomal miR-663b exacerbates PASMC dysfunction and PH progression by suppressing the AMPK/SIRT1 axis and promoting inflammation.^1,264^

## Wnt signaling pathway in PH

**Fig. 7:**
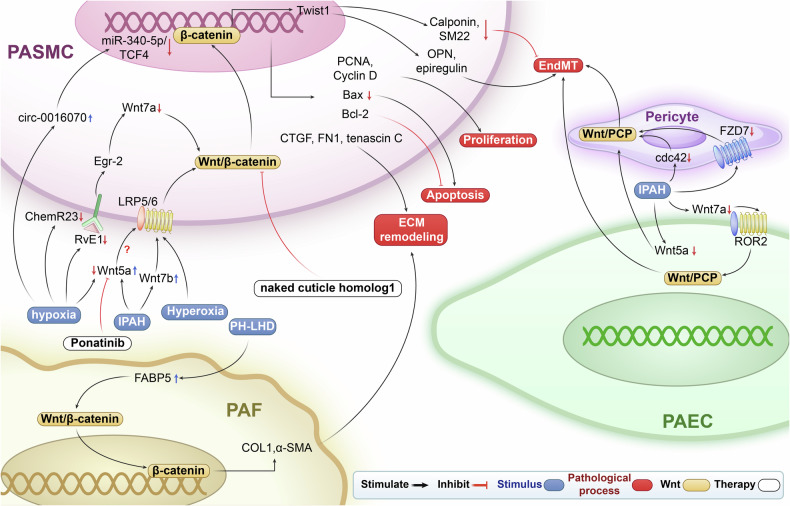
Wnt signaling pathway and targeted therapy in PH. In hypoxia-PH, hyperoxia and IPAH, the Wnt/β-catenin pathway is activated, binding to the transcription factor TCF4 and leading to pro-proliferative and anti-apoptotic effects. Activated Wnt/β-catenin also promotes EndMT and ECM remodeling. Activation of the Wnt/β-catenin pathway in PAF ultimately promotes ECM remodeling. Additionally, in PAH, Wnt/PCP signaling is inhibited in PAECs and pericytes, suppressing EndMT. Naked cuticle homolog 1 and ponatinib mitigate PH by inhibiting Wnt/β-catenin pathway. *PASMC* pulmonary artery smooth muscle cell, *PAEC* pulmonary artery endothelial cell, *PAF* pulmonary artery fibroblast, *BMPR2* bone morphogenetic protein receptor type 2, *PCP* planar cell polarity, *EndMT* endothelial-to-mesenchymaltransition, *α-SMA* α-smooth muscle actin, *IPAH* idiopathic pulmonary arterial hypertension, *ECM* extracellular matrix, *TCF4* transcription factor 4, *ChemR23* chemerin chemokine-like receptor 1, *RvE1* Resolvin E1, *CTEPH* chronic thromboembolic pulmonary hypertension, *SM22* smooth muscle protein 22, *PH-LHD* left ventricular secondary pulmonary hypertension, *Su/Hx* SU5416-hypoxia, *CTGF* connective tissue growth factor, *WISP-1* Wnt-induced signaling protein 1, *PCNA* proliferating cell nuclear antigen, *Egr-2* early growth response 2, *LRP5/6* low-density lipoprotein-related receptors 5 and 6, *FN1* fibronectin1, *ERK* extracellular signal-regulated kinase, *ROR2* receptor tyrosine kinase-like orphan receptor type 2, *COL1* type I collagen, *Drp1* dynamin-related protein 1, *FABP5* fatty acid-binding protein 5, *OPA* optic atrophy 1, *OPN* osteopontin

Wnt protein is a secreted glycoprotein that plays an important role in embryonic development and tumorigenesis. The Wnt signaling pathway is highly conserved and can be divided into canonical Wnt/β-catenin pathway, non-canonical Wnt/planar cell polarity (PCP) pathway, and Wnt/Ca^2+^ pathway.^265^ The Wnt signaling pathway has been proven to be a key regulator of many diseases, such as diabetes nephropathy.^266^ Previous studies have shown that the Wnt signaling pathway also plays an important role in PH. In 2008, Rai et al. found that Wnt7a was absent in ECs with cluster lesions in patients with severe PAH.^267^ In 2009, Laumanns et al. found that Wnt/PCP pathway mediators in IPAH were significantly upregulated in the pulmonary resistance vessel endodermis.^268^ The expression of Wnts varies across different etiologies of PAH, influencing the activation or inhibition of the Wnt signaling pathway. Kocak et al. found that the expression of Wnt1 and Wnt2 genes was significantly increased in patients with scleroderma-PAH.^269^ However, the expression of Wnt7a also varies in different PH models.^267,270^ In the classic Wnt/β-catenin pathway, activation leads to β-catenin translocating to the nucleus, where it binds transcription factors to enhance the expression of target genes. This mechanism fosters various pathological processes in PAH, such as cell proliferation, ECM remodeling, EndMT, and inflammation. Furthermore, activation of non-canonical Wnt signaling pathways also supports PAH development and is crucial for ECM remodeling, EndMT, inflammation, and mitochondrial dysfunction (Fig. 7).

### Wnt/β-catenin promotes cell proliferation in PH

Activation of the Wnt/β-catenin pathway facilitates downstream gene expression by promoting the translocation of β-catenin to the nucleus, which in turn enhances the proliferation of PASMCs, contributing to the development of PAH. In the plasma of patients with IPAH and the lung tissue of mice with hypoxia-PH, levels of the anti-inflammatory substance Resolvin E1 and its receptor, ChemR23, were found to be downregulated. This downregulation reduces the inhibition of PKA-mediated Egr-2 phosphorylation. Additionally, the upregulation of Wnt7a promotes PASMC proliferation via the canonical Wnt/β-catenin signaling pathway.^270^ In PASMCs from PH rats, the Wnt/β-catenin pathway is activated, allowing β-catenin to enter the nucleus and interact with TCF4. This interaction promotes cell proliferation and survival by increasing the expression of PCNA and Bcl-2 while decreasing the expression of Bax.^271^ In hypoxia-induced human PASMCs, the level of Wnt5a is downregulated, which reduces the inhibition of β-catenin, and then upregulates Cyclin D to promote cell proliferation.^272^ Takahashi et al. observed that Wnt5a expression was elevated in IPAH PASMCs.^273^ They found that Wnt5a inhibited β-catenin activation and proliferation in healthy PASMCs induced by PDGF, but it could not inhibit PDGF-B-dependent β-catenin activation in IPAH PASMCs.^273^

The Wnt/β-catenin pathway contributes to the development of hyperoxygen-induced bronchopulmonary dysplasia and PH by enhancing cell proliferation and ECM remodeling. In hyperoxygen-induced PASMCs, the activation of β-catenin also increases the expression of CTGF and FN1, further driving ECM remodeling and cell proliferation.^274^

### Wnt/β-catenin promotes ECM remodeling in PH

Activation of the Wnt/β-catenin pathway promotes ECM remodeling. In samples from PAH patients and allergen-induced asthma mouse models, the upregulation of Wnt7b enhances the expression of the ECM protein tenascin C through β-catenin activation.^275^ The elevated levels of fatty acid-binding protein 5 in PAFs of mice with left ventricular secondary PH activate the Wnt/β-catenin pathway. This activation results in increased expression of COL1 and α-SMA, ultimately contributing to fibrosis.^276^ Furthermore, in PASMCs exposed to high oxygen levels, β-catenin activation upregulates CTGF and FN1 expression, promoting ECM remodeling and cell proliferation.^274^ The Wnt/β-catenin pathway also contributes to right heart failure in PAH by facilitating ECM remodeling. In rat models of PAH induced by pulmonary artery banding or MCT, Wnt/β-catenin activation enhances the mRNA and protein expression of periostin, CTGF, and COL3 by activating FOSL1 and FOSL2.^277^ This process drives the differentiation of fibroblasts into myofibroblasts, ultimately leading to PAH and RV failure.^277^

### Wnt regulates EndMT in PH

#### Wnt/β-catenin promotes EndMT in PH

Activation of the Wnt/β-catenin pathway has been shown to promote EMT. The expression of circ_0016070 increases in PASMCs under hypoxic conditions, which inhibits the expression of miR-340-5p. This inhibition subsequently promotes TCF4 expression, forming a transcriptional complex with β-catenin. As a result, Twist1 expression increases, along with the upregulation of OPN and epiregulin, while Calponin and SM22α levels decrease.^278^

#### Downregulated Wnt/PCP promotes EndMT in PH

In PAH, the inhibition of the Wnt/PCP pathway in PMVECs and pericytes results in vascular damage. The expression of Wnt7a protein is diminished in PMVECs of PAH patients, leading to reduced binding to ROR2. This inhibition of Wnt/PCP decreases cellular motility and results in reduced formation of filamentous pseudopodia.^279^ The expression of FZD7 and cdc42 in pericytes is reduced in PAH, leading to the inhibition of the Wnt/PCP pathway. This results in impaired migration and attachment to the inner duct.^280^ Additionally, decreased expression of Wnt5a in PAH PMVECs inhibits Wnt/PCP in co-cultured pericytes, causing diminished migration and polarization of pericytes toward the tubular structures formed by PMVECs, which in turn reduces the activity of newly formed blood vessels.^281^

## Other signaling pathway in PH

### FGF2/FGFR signaling pathway in PH

FGF2 and its receptor, FGFR, play a dual role in the pathogenesis of PH, contributing to adaptive responses in certain contexts while driving pathological progression in conditions like PAH.^212,282–284^ FGF2 is a potent mitogen that influences various cellular processes, including proliferation, migration, and survival, especially in PASMCs and PAECs.^282–284^

#### Protective role of FGF2/FGFR signaling in hypoxia-induced PH

Endothelial FGFR1/2 signaling is crucial for the adaptive response to hypoxia by mitigating TGF-β-mediated EndMT and vascular remodeling. In hypoxic conditions, endothelial FGFR deficiency in mice or FGFR inhibition in human PAECs amplifies TGF-β signaling, resulting in increased EndMT and exacerbated vascular remodeling. This underscores the importance of endothelial FGFR signaling in maintaining vascular integrity and homeostasis under hypoxic stress.^283^

#### Pathological role of FGF2/FGFR signaling in PAH

While FGF2 plays a protective role in hypoxia, its excessive expression drives pathological remodeling in PAH. In IPAH, endothelial overproduction of FGF2 drives PASMC hyperplasia, contributing to disease progression.^284^ In human PAH, distal pulmonary arteries exhibit a notable increase in pericytes, which show heightened proliferation and migration when exposed to conditioned media from PAH endothelial cells. Neutralizing FGF2 reduces these effects, emphasizing the role of FGF2 signaling in driving pericyte involvement in pulmonary vascular remodeling. This excessive pericyte coverage, linked to endothelial dysfunction, plays a key role in the vascular remodeling observed in PAH.^60^ Similarly, the upregulation of FGF2 in the lung tissue of both experimental models and human PAH patients results in the activation of FGFR signaling pathways, which are essential for the progression of vascular changes and disease exacerbation.^285^ The downstream signaling pathways of FGFR, such as the activation of ERK1/2 and Akt, play pivotal roles in cellular proliferation, apoptosis regulation, and the maintenance of vascular homeostasis.^212,282,286^ In PAH, APLN deficiency in PAECs increases FGF2 and FGFR1 expression through downregulation of miR-424 and miR-503, promoting PASMC proliferation.^287^ This FGF2-mediated signaling may contribute to pulmonary arterial remodeling observed in HPAH.^288^

## RhoA/ROCK signaling pathway in PH

### RhoA/ROCK promotes cell proliferation and endothelial dysfunction in PH

In 2002, Takemoto et al. linked the RhoA/ROCK signaling pathway to hypoxia-PH by demonstrating that Rho-kinase mediates the downregulation of eNOS, a critical factor in pulmonary vascular regulation.^289^ This finding highlighted the critical role of RhoA/ROCK signaling in the pathogenesis of PH, where it contributes to vascular remodeling and increased PVR, driving disease progression.^289–291^ RhoA, a small GTPase, activates ROCK, which regulates cytoskeletal dynamics, cell contraction, and proliferation. In PH, this pathway is upregulated, contributing to oxidative stress, PASMC hypercontractility, proliferation, and migration.^292^ These processes lead to the narrowing of the pulmonary vasculature and increased PAP.^292^ In a chronic neonatal rat model of PH, the upregulation of RhoA/ROCK activity specifically in the right ventricle plays a pivotal role in hypoxia-induced systolic dysfunction, partly through the regulation of PDE5 activity.^291^ A study identifies that smooth muscle-enriched lncRNA activates the RhoA/ROCK pathway in PASMCs by targeting and downregulating miR-141, leading to increased cell proliferation and migration.^292^

## PPAR signaling pathway in PH

PPARs are a family of ligand-activated transcription factors that regulate gene expression by binding to specific response elements in the DNA.^293^ These receptors play vital roles in various cellular processes such as metabolism, inflammation, and cellular differentiation.^293,294^ Currently, three main isoforms of PPARs: PPARα, PPARβ/δ, and PPARγ have been identified,^295^ each exhibiting distinct and occasionally overlapping functions in the pulmonary vasculature. The role of PPAR signaling in PH has evolved over time. In 2003, Ameshima et al. linked reduced PPARγ expression to EC proliferation and vascular remodeling.^296^ By 2006, Ali et al. highlighted PPARβ signaling as a potential therapeutic target for reducing pulmonary vascular remodeling.^297^ Later, in 2014, Li et al. demonstrated that PPARα signaling regulates miR-199a-2 and attenuates ET-1,^298^ offering further insight into PPARs’ role in PH. Thus, PPAR signaling is crucial for regulating vascular homeostasis and modulating the responses of PASMCs, PAECs, and fibroblasts.^299^

### PPARγ against cell proliferation and pulmonary vascular remodeling in PH

PPARγ is expressed in the lung and pulmonary vasculature, and its expression is reduced in the vascular lesions of patients with PH and preclinical PH models.^300–304^ Targeted deletion of PPARγ in SMCs induces PH, highlighting its crucial role in vascular protection and suggesting its activation as a potential therapeutic strategy for PH.^305,306^ The reduction in PPARγ expression in PH is driven by ERK1/2,^301^ NF-κB p65,^301^ NOX4,^301^ ROCK^302^, and ET-1^303^pathways. A recent study shows that SMYD2 expression is elevated in PASMCs during PH, promoting proliferation and pulmonary vascular remodeling by inhibiting PPARγ activity through monomethylation, which enhances mitophagy.^307^ Additionally, reduced BMPR2 expression in PAH impairs PAEC function, disrupting PPARγ/β-catenin-mediated APLN production, which promotes PAEC survival.^308^ BMP-2 signaling through BMPR2 prevents PASMC proliferation by activating PPARγ and promoting apoE production, which protects against PAH.

### PPARα and PPARβ/δ attenuate inflammation and vascular remodeling in PH

PPARα signaling also plays a crucial role in mitigating PH through diverse mechanisms across various models and conditions. Activation of PPARα by fenofibrate upregulates miR-199a2 and miR-301a/miR-454, targeting HIF-1α, ET-1, and PAI-1, which are key mediators of vascular remodeling and inflammation in PH. These miRNAs are co-transcriptionally regulated by PPARα through promoters such as DNM3OS and SKA2, highlighting its transcriptional influence.^298^ Similarly, PPARβ/δ signaling has emerged as a compelling target with unique benefits for managing PH. PPARβ/δ plays a pivotal role in PGI signaling, mediating the activation of K(Ca) channels in PASMCs, which contributes to acute vasodilation independently of NO.^309^

## Estrogen receptors signaling pathway in PH

Estrogen exerts its protective effects in PH through both genomic and non-genomic pathways mediated by ERα, ERβ, and the GPER1.^65^ In 2006, Morani et al. first linked ERβ signaling to PH by demonstrating lung dysfunction and systemic hypoxia in *ER*β knockout mice.^310^ A decade later, in 2017, Alencar et al. uncovered the involvement of GPER1 signaling in PH,^311^ highlighting the diverse estrogen receptor pathways in vascular remodeling associated with the disease. These receptors are involved in regulating vascular function, reducing inflammation, and improving cardiac adaptation, thereby highlighting their therapeutic potential in PH management. Each receptor contributes to specific cellular processes in the pulmonary vasculature and RV, offering a multi-faceted approach to mitigating PH-related dysfunction.^311–313^

### ERα mediates the protective effects of estrogen in PH

ERα plays a crucial role in mediating the protective effects of estrogen in PH, particularly in endothelial cells, PASMCs, and RV myocytes. Its activation enhances the expression of BMPR2 and APLN, both of which are essential for vascular homeostasis and RV adaptation.^314,315^ Research in an MCT-PAH model demonstrates that estrogen replacement therapy in ovariectomized female rats significantly reduces RVH and PAP, while estrogen deficiency accelerates disease progression.^313^ These findings underscore the role of ERα in mitigating pulmonary vascular remodeling and preserving RV function. Additionally, ERα signaling helps suppress endothelial cell apoptosis and promotes NO production, further improving pulmonary vascular integrity and resilience in PH.^314^ These effects underscore the importance of ERα in protecting pulmonary and cardiac structures from the detrimental consequences of PH.

### ERβ mediates key anti-inflammatory and antioxidant responses in PH

ERβ complements the protective effects of ERα by mediating key anti-inflammatory and antioxidant responses in PH. ERβ is essential for preserving the ECM composition in the lung, and its loss results in altered lung structure and systemic hypoxia.^310^ In rat models of MCT-PAH, ERβ activation mitigates vascular remodeling, suppresses inflammation, and improves RV function.^313^ These mechanisms emphasize the role of ERβ in reducing oxidative damage and maintaining RV resilience during PH progression. By modulating these pathways, ERβ significantly enhances both cardiac and pulmonary vascular adaptation, making it a promising therapeutic target in PH.

### GPER1 improves RV function in PH

The genomic estrogen receptor, GPER1 adds another layer of estrogen-mediated protection in PH, especially through non-classical signaling pathways. Activation of GPER1 has been shown to improve RV function, reduce PAP, and attenuate pulmonary vascular remodeling in both male and female animal models.^311^ GPER1 signaling enhances NO bioavailability, reduces inflammation, and promotes vascular repair by inhibiting fibrosis.^311^ Unlike ERα and ERβ, GPER1 activation does not induce systemic estrogenic effects, further supporting its potential as a therapeutic target in PH.^316^

## JAK/STAT signaling pathway in PH

### JAK/STAT promotes inflammation and cell proliferation in PH

In 2007, Masri et al. first showed JAK/STAT signaling in PH, highlighting STAT3 activation in IPAH ECs.^27^ The growing body of evidence implicates aberrant JAK/STAT signaling in various forms of PAH, including those induced by hypoxia, schistosomiasis, and mutations in the *CAV1* gene,.^317^ RNA-Seq analysis in schistosomiasis-induced PAH has revealed significant changes in JAK/STAT-associated genes.^318^ In CAV1-deficient PAECs, STAT3 activation is coupled with increased inflammatory cytokine CXCL10 production and endothelial dysfunction, further exacerbating vascular remodeling.^317^ JAK2 over-activation in PASMCs from IPAH patients, promotes IL-6-induced proliferation and migration of PASMCs, thereby elevating PAP and RVH.^319^

## CaSR signaling pathway in PH

Yamamura et al. in 2012 first demonstrated that CaSR signaling contributes to enhanced Ca²⁺ influx and PASMC proliferation in PH.^320^ The CaSR plays a critical role in the development and progression of PH, particularly in IPAH, hypoxia-PH, and PPHN, where its expression is upregulated.^321,322^ Several genetic variants of the *CaSR* gene, including rs1042636, rs6776158, rs1048213, and rs9883099, have been linked to an increased risk and severity of IPAH.^323^ Notably, individuals with the rs1042636 variant exhibit higher mPAP and reduced survival, highlighting the significant role of these variants in IPAH pathogenesis.^323^

### CaSR promotes cell proliferation and anti-apoptosis to contribute to vascular remodeling

These variants contribute to the upregulation of CaSR expression and activity, which in turn enhances Ca²⁺ influx and Ca²⁺-induced cytosolic Ca²⁺ release into PASMCs, promoting cell proliferation and contributing to vascular remodeling.^323^ Furthermore, chronic exposure to phenylalanine induces PH through CaSR activation in PASMCs, which leads to increased intracellular calcium and pulmonary vascular remodeling.^209^ Hypoxia-induced PASMC proliferation is mediated by upregulation of the CaSR-TRPC1/6 pathway, enhancing cell viability and DNA synthesis.^324^ CaSR activation in PAECs contributes to endothelial damage and vascular dysfunction in MCT-PAH models.^325^ Additionally, cigarette smoke-induced PH involves spermine-enriched endothelial EVs that activate CaSR, promoting PASMC proliferation and resistance to apoptosis.^201,208^

## Hippo signaling pathway in PH

### Hippo promotes cell proliferation and vascular remodeling in PH

In 2016, Kudryashova et al. first showed Hippo signaling in PH,^326^ and it is increasingly recognized as a pivotal regulator of pulmonary vascular remodeling in PH. Dysregulation of the Hippo signaling pathway, particularly through the inactivation of LATS1/2, results in the activation of YAP. This effectively inhibits the canonical Hippo pathway, driving key pathological processes in PAH. YAP activation promotes PASMC proliferation and survival by engaging signaling cascades such as mTOR-Akt, stabilizing HIF-1α, Notch3 intracellular domain, and β-catenin, and suppressing pro-apoptotic factors like Bim.^326^ Additionally, Siah2-mediated proteasomal degradation of LATS1/2 leads to YAP dephosphorylation and nuclear localization, further promoting PASMC proliferation and vascular remodeling in PAH models.^327^ Noncanonical Hippo/MST signaling via BUB3 and FOXO has also been shown to support pulmonary vascular cell growth and survival, indicating alternative regulatory mechanisms within the pathway.^328^

## Nrf2/HO-1 signaling pathway in PH

### Nrf2/HO-1 protects against oxidative stress, iron dysregulation, and vascular remodeling in PH

In 2009, Sussan et al. first showed Nrf2 signaling in PH, demonstrating its protective role against cigarette smoke-induced PH in mice.^329^ The Nrf2/HO-1 signaling pathway is an emerging focus in the study of PH, particularly for its role in iron metabolism and oxidative stress regulation.^330^ Nrf2 activation drives the expression of HO-1, a key enzyme that degrades heme into biliverdin, carbon monoxide, and free iron.^331^ This process not only detoxifies excess heme but also influences iron homeostasis in the pulmonary vasculature.^332^ In PH, oxidative stress and iron dysregulation are key contributors to pathological vascular remodeling and endothelial dysfunction,^333^ as highlighted by several clinical studies showing a prevalence of iron deficiency among PH patients.^334–337^ The interplay between oxidative stress and iron metabolism in PH underscores the importance of the Nrf2/HO-1 axis as an emerging pathway. Numerous clinical and pre-clinical studies have implicated the dysregulation of iron-regulatory proteins, such as ferritin, FPN1, hepcidin, and TfR1, as a contributing factor in the pathogenesis of PH.^336,338–340^ For instance, in a clinical study, elevated hepcidin levels have been associated with iron deficiency in IPAH, exacerbating vascular remodeling and disease progression.^336^ Although the Nrf2/HO-1 pathway has been shown to regulate iron storage and efflux through the transcription of genes like ferritin, FPN1, and hepcidin,^341,342^ its specific role in PH, particularly concerning iron deficiency, remains unclear.

## PARP1/PKM2 signaling pathway in PH

### PARP1/PKM2 promotes maladaptive vascular inflammation and RV remodeling in PAH

In 2022, Shimauchi et al. demonstrated PARP1/PKM2 signaling in PH, with the pathway gaining recognition as a critical player in the development of PH.^343^ Specifically, their study identified the PARP1/PKM2 axis as a key driver of maladaptive RV remodeling in PAH. Elevated PARP1 and PKM2 expression were observed in decompensated RVs of patients and animal models.^343^ Over-activated PARP1 promotes PKM2 nuclear function, glycolytic gene expression, and NF-κB-dependent inflammation, impairing CMs function.^343^ PARP1, a key mediator of the DNA damage response, is over-activated in conditions of oxidative stress and hypoxia,^343^ which are hallmarks of PH. This over-activation depletes NAD^+^ reserves, contributing to metabolic reprogramming that favors glycolysis over oxidative phosphorylation.^344^ Concurrently, PKM2, a glycolytic enzyme with non-metabolic roles, undergoes nuclear translocation under hypoxic conditions, where it acts as a transcriptional coactivator of pro-inflammatory and proliferative genes.^343,345^ PARP1-mediated post-translational modifications of PKM2, such as PARylation, enhance its nuclear localization and activity,^346^ driving the expression of genes involved in PASMC proliferation, endothelial dysfunction, and inflammatory cytokine production, which are central to pulmonary vascular remodeling.^343^ This signaling axis may further exacerbate PH by promoting the Warburg effect, a metabolic shift to aerobic glycolysis even in oxygen-sufficient environments, which supports the high energy demands of proliferating VSMCs and inflammatory cells.^343^ Moreover, PARP1 and PKM2-driven inflammation contribute to macrophage polarization toward a pro-inflammatory M1 phenotype, amplifying vascular injury and remodeling.^343,347^

## cGAS-STING signaling pathway in PH

### cGAS-STING promotes inflammation and hyperproliferation in PH

In 2024, Yan et al. demonstrated the role of the cGAS-STING pathway in PH, with calcitonin gene-related peptide (CGRP) inhibiting vascular remodeling via this pathway.^348^ This finding aligns with the growing recognition of the cGAS-STING signaling pathway as a critical contributor to the pathological remodeling of the pulmonary vasculature in PH.^348,349^ Under conditions such as hypoxia, MCT, or PDGF-induced stress, mitochondrial damage in PASMCs leads to the release of mitochondrial DNA into the cytoplasm, triggering the activation of cGAS-STING.^349^ This cascade promotes the secretion of pro-inflammatory cytokines and the activation of NF-κB, which exacerbates hyper-proliferation, migration, and phenotypic switching of PASMCs.^349^ These cellular events underlie vascular remodeling, a hallmark of PH progression.^349^ In this context, studies reveal that targeting the cGAS-STING pathway can attenuate vascular remodeling and improve hemodynamic parameters, highlighting its therapeutic potential.^348,349^

#### The networks among signaling pathways in PH

Research into signaling pathways in PH reveals extensive crosstalk, where pathways collectively contribute to disease pathology. The BMPR2 pathway interacts with TGF-β, MAPK, PI3K/Akt, NF-κB, Notch, HIF-1α, and Wnt, forming a network that regulates cellular proliferation and anti-apoptosis. Similarly, TGF-β engages with PI3K, NF-κB, HIF-1α, MAPK, and Wnt to drive processes like inflammation, EndMT, ECM remodeling, and fibrosis. The PI3K pathway crosstalks with NLRP3, MAPK, Notch, NF-κB, and HIF-1α, influencing cellular proliferation and inflammation, while NF-κB interacts with BMPR2, TGF-β, PI3K/Akt, HIF-1α, and MAPK to promote inflammation. In contrast, AMPK inhibits inflammation, fibrosis, and vascular remodeling by suppressing pathways such as NF-κB, NLRP3, Akt/mTOR, and Notch.

## BMPR2 crosstalk with other signaling pathways

### BMPR2 crosstalk with TGF-β/MAPK/PI3K/Akt

**Fig. 8:**
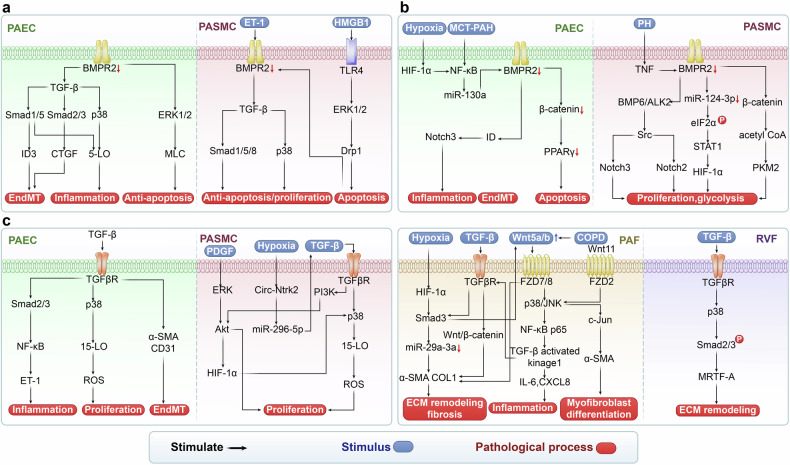
BMPR2 and TGF-β crosstalk with other signaling pathways in PH. **a**, **b** The networks of BMPR2 and other signaling pathways in PH. In PAECs, reduced BMPR2 signaling activates TGF-β, triggering EndMT and inflammation through Smad1/5, Smad2/3, and p38 pathways. Additionally, reduced BMPR2 signaling activates ERK1/2 pathways, promoting anti-apoptosis. In PASMCs, reduced BMPR2 signaling, influenced by ET-1 and HMGB1-induced apoptosis, supports proliferation and survival via the TGF-β/Smad1/5/8, and p38 pathways. Hypoxia and MCT stimulate HIF-1α and NF-κB, reducing BMPR2 expression through miR-130a, which promotes EndMT, inflammation, and apoptosis by suppressing β-catenin pathways in PAECs. In PASMCs, PH-induced TNF downregulates BMPR2 signaling, driving cell proliferation and glycolysis through the activation of BMP6/ALK2 and β-catenin, while suppressing miR-124-3p. **c** The networks of TGF-β and other signaling pathways in PH. Factors such as TGF-β, PDGF, hypoxia, and the condition of COPD contribute to cell proliferation, inflammation, EndMT, ECM remodeling, fibrosis, and myofibroblast differentiation across various cell types (PAECs, PASMCs, PAFs and RVFs), primarily through the activation of the TGFβR and its interactions with key pathways like PI3K/Akt, MAPKs, HIF-1α, and Wnt/β-catenin. *PASMC* pulmonary artery smooth muscle cell, *BMPR2* bone morphogenetic protein receptor type 2, *TGF-β* transforming growth factor beta, *TGFβR* transforming growth factor beta receptor, *PAEC* pulmonary artery endothelial cell, *ET-1* endothelin-1, *HMGB1* high-mobility group box 1, *Akt* protein kinase B, *ERK1/2* extracellular signal-regulated kinase 1/2, *p38* mitogen-activated protein kinase, *NF-κB* nuclear factor kappa-light-chain-enhancer of activated B cells, *HIF-1α* hypoxia-inducible factor 1-alpha, *MAPKs* mitogen-activated protein kinases, *miR* microRNA, *ID* inhibitor of differentiation, *PI3K* phosphoinositide 3-kinase, *Notch3* Notch receptor 3, *PDGF* platelet-derived growth factor, *ROS* reactive oxygen species, *Wnt5a/b* Wnt family member 5A/B, *COPD* chronic obstructive pulmonary disease, *IPAH* idiopathic pulmonary arterial hypertension, *ECM* extracellular matrix, *EndMT* endothelial-to-mesenchymal transition, *Skp2* S-phase kinase-associated protein 2, *Hes1* hairy and enhancer of split 1, *p27Kip1* cyclin-dependent kinase inhibitor 1B, *N1-ICD* Notch1 intracellular domain, *p21* cyclin-dependent kinase inhibitor 1A, *Bcl-2* B-cell lymphoma 2, survivin Baculoviral IAP repeat-containing 5

The expression of BMPR2 is usually reduced in multiple PH groups, which participates in pulmonary vascular remodeling by inhibiting the BMPR2 signaling pathway while activating other signaling pathways especially TGF-β. Therefore, an imbalance in TGF-β and BMPR2 signal transduction is believed to be the molecular level characteristic of PAH. In addition, BMPR2 crosstalks with MAPK and PI3K/Akt signaling pathways, promote hyper-proliferation, resistance to apoptosis, and inflammation in both PAECs and PASMCs (Fig. 8a).

#### PAECs

In PAECs from rats with a *Bmpr2* mutation, TGF-β/Smad2/3 and p38-dependent signaling pathways are enhanced, thereby elevating the nucleoplasmic expression of 5-LO to increase transcripts of *Il1r1, Il6r, Tlr2*, and *Tlr4*, resulting in the promotion of PAEC proliferation.^91,350^ In *Bmpr2* -deficient human PAECs stimulated with TGF-β, EndMT is driven by the upregulation of Slug and Twist, along with increased phosphorylation of cofilin and MLC through the ALK2/3/Smad1/5/ID3 and ALK1/Smad2/3/CTGF signaling pathways.^350^ Knockdown of *Bmpr2* in PAECs stimulates proliferation via activation of ERK pathways mediated by non-muscle MLCK and downregulation of Sprouty 1.^351^ In human PAECs lacking BMPR2, TNF stimulation prolongs p38-MK2 activation to trigger the GADD34-protein phosphatase 1 complex, which dephosphorylates eIF2α, impairing stress granule formation and enhancing the mRNA translation of cytokines like IL-6 and IL-8, eventually exacerbating inflammatory cell recruitment and PAH.^352^

#### PASMCs

In human PASMCs pre-treated with ET-1, BMPR2 expression is reduced, while BMP2 binds to BMPR1B, thereby activating the p38 pathway to drive PASMC proliferation, with the effect amplified under pathological conditions.^353^ In PASMCs from MCT-PAH rat models, HMGB1 triggers ERK1/2 signaling, leading to Drp1 phosphorylation and mitochondrial fragmentation, which promotes autophagy activation, resulting in BMPR2 degradation, ID1 downregulation, and PASMC proliferation/migration.^354^ These pathways appear to operate in parallel rather than as a direct upstream-downstream relationship. The p38 pathway plays a central role in inflammatory signaling associated with BMPR2 deficiency. In human PASMCs where BMPR2 is silenced by small interfering RNA (siRNA), BMP2 stimulation activates the p38 pathway, increasing IL-6 expression.^355^

### BMPR2 and NF-κB, Notch, HIF-1α, Wnt

BMPR2 also modulates inflammation and proliferation in PAECs by interacting with the NF-κB, Notch, HIF-1α, and Wnt signaling pathways. In PASMCs, these interactions regulate inflammation, enhance glycolysis, and drive cell proliferation (Fig. 8b).

#### PAECs

The activation of NF-κB in MCT-PAH leads to disruption of the BMPR2–ID–Notch3 axis, amplification of the inflammatory response, increased cell death in PAECs, and promotion of EndMT.^356^ This process also promotes TGF-β-induced EndMT and is associated with increased expression of inflammatory factors, including IL-1β, IL-6, and TNF in MCT-PAH mice.^356^

Knockout of *BMPR2* in PAECs reduced the expression of miR-124, increased the expression of PTPB1 and PKM2, significantly disrupted glycolysis genes, and enhanced glycolysis leading to excessive PAEC proliferation.^48^ In BMP2-stimulated human PAECs and PMVECs, BMPR2 activation increases nuclear β-catenin levels, promoting the expression of downstream target genes (c-Myc, Cyclin D, and survivin). It also facilitates the formation of a β-catenin/PPARγ complex, which upregulates APLN expression. Collectively, these processes enhance PAEC survival and inhibit PASMC proliferation through paracrine signaling.^308,357,358^

#### PASMCs

TNF downregulates BMPR2 expression by inhibiting its transcription and promoting proteolysis, while simultaneously upregulating BMP6 and ActA receptor 2A expression.^238^ This cascade enhances BMP6/ActA receptor 2A-ALK2 signaling and activates Src, resulting in increased Notch2 and downstream HEY1/2 expression, while inhibiting Notch3 signaling, thereby promoting the proliferation of PASMCs.^238^ In PASMCs derived from *Bmpr2* heterozygous mutant mice, decreased miR-124-3p expression is associated with activation of PKR-like ER kinase, promotion of eIF2α phosphorylation, and increased PDGFR expression. This cascade activates STAT1, leading to upregulation of KLF4 and HIF-1α, enhancing glycolysis and promoting proliferation and viability of PASMCs.^359^ BMPR2 deficiency activates β-catenin, causing it to translocate into the nucleus, where it upregulates ALDH1A3, increasing ACC. This ACC acetylates histone H3K27, promoting NFYA expression, which ultimately upregulates DLD, PKM2, and IDH1, enhancing glycolysis and proliferation of PASMCs.^360^

### TGF-β Crosstalk with PI3K, NF-κB, HIF-1α, MAPK, Wnt

In addition to its interaction with BMPR2 signal transduction, TGF-β can also interact with other signaling pathways (PI3K, NF-κB, HIF-1α, MAPK, and Wnt) to contribute to the EndMT, proliferation, and inflammation of PAECs, as well as promoting the proliferation of PASMCs and enhancing ECM remodeling of PAFs and RVFs (Fig. 8c).

#### PAECs

In PAECs from IPAH patients and MCT-PAH rats, activation of the TGF-β/Smad2/3 pathway enhanced the transcriptional activity of Cyclin D, Snail, and NF-κB promoters. This activation increased NF-κB promoter activity, elevated ET-1 levels, and upregulated the expression of endothelial adhesion molecules and pro-inflammatory cytokines, including VCAM-1, ICAM-1, CCL5, and MCP-1. These changes contributed to heightened cell proliferation, inflammation, and EndMT.^361,362^ In human PAECs exposed to hypoxia, elevated HIF-1α activates the TGF-β/Smad2/3 pathway, resulting in the upregulation of α-SMA, Slug, and vimentin, alongside the downregulation of CD31 and VE-cadherin, thereby inducing EndMT.^363^ Additionally, HIF-1α activates the p38/15-LO pathway, which increases the expression of NDUFA4L2, promoting lipid oxidation and ROS production, ultimately driving cell proliferation.^364^

#### PASMCs

In PASMCs of PAH, increased TGF-β leads to thickening of pulmonary vascular walls by enhancing cell survival and proliferation through the PI3K/Akt pathway.^365,366^ In mouse hypoxic lung tissue and PASMCs, upregulated Circ-Ntrk2 activates TGF-β expression by targeting miR-296-5p, which subsequently upregulates p38 and finally promotes PASMC proliferation and pulmonary vascular remodeling.^367^ In human PASMCs exposed to hypoxia or treated with PDGF, phosphorylation of ERK/Akt increases, contributing to the upregulation of HIF-1α,^368^ which activates p38/15-LO pathway to upregulate NDUFA4L2, thereby promoting lipid oxidation and ROS, leading to increased cell proliferation.^364^

#### PAFs

In PAFs, hypoxia upregulates HIF-1α and subsequently downregulates miR-29a-3p expression by increasing Smad3, thereby increasing α-SMA and COL1 expression to promote ECM remodeling.^131^ Additionally, studies indicate that TGF-β stimulation of PAFs activates the Wnt/β-catenin pathway, facilitating pulmonary fibrosis by downregulating E-cadherin while upregulating N-cadherin and α-SMA.^12,369^ TGF-β stimulation of human PAFs inhibits the phosphorylation of GSK3β at Ser9/21, thereby activating the Wnt/β-catenin pathway. This activation leads to increased expression of FN1 and α-SMA, promoting fibrosis.^12^

Additionally, Wnt5a/b ligand expression is elevated in PAFs from COPD patients.^370^ Wnt5b induces JNK, p38, and p65 NF-κB signaling mediated by the FZD2 receptor and TAK1, resulting in the release of IL-6 and CXCL8.^370^ The upregulated expression of Wnt11 in PAF isolated from IPF patients and animal models leads to upregulation of α-SMA by activating the JNK/c-Jun pathway, thereby promoting myofibroblast differentiation.^371^

#### RVFs

In TGF-β-induced mouse RVFs, p38 promotes collagen production and the formation of stress fibers by participating in Smad2/3 phosphorylation and nuclear translocation of MRTF-A.^372^

### PI3K/Akt crosstalk with NLRP3, MAPK, Notch, NF-κB, and HIF-1α

**Fig. 9:**
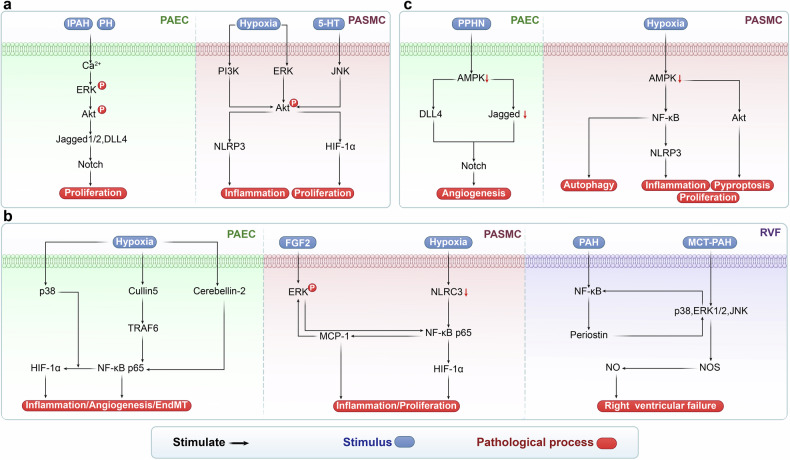
PI3K, NF-κB and AMPK crosstalk with other signaling pathways in PH. **a** The networks of PI3K and other signaling pathways in PH. In PAECs and PASMCs, IPAH, hypoxia, and 5-HT activate ERK, PI3K, JNK, and calcium signaling, promoting proliferation and inflammation through the Akt pathway. **b** The networks of NF-κB and other signaling pathways in PH. Hypoxia and FGF2 activate NF-κB p65 signaling in PAECs and PASMCs through interactions with key pathways, including HIF-1α, p38, and ERK. These networks drive processes such as inflammation, proliferation, angiogenesis, and EndMT. In RVF, MCT induces right ventricular failure through NF-κB activation and its interactions with MAPK signaling pathways. **c** The networks of AMPK and other signaling pathways in PH. In PAECs and PASMCs, hypoxia and PPHN suppress AMPK signaling, leading to the activation of key pathways such as Notch, NF-κB, NLRP3, and Akt, which drive angiogenesis, autophagy, inflammation, pyroptosis, and increased cell proliferation. *IPAH* idiopathic pulmonary arterial hypertension, *PAEC* pulmonary artery endothelial cell, *PASMC* pulmonary artery smooth muscle cell, *5-HT* serotonin, *ERK* extracellular signal-regulated kinase, *PI3K* phosphoinositide 3-kinase, *JNK* c-Jun N-terminal kinase, *Akt* protein kinase B, *FGF2* fibroblast growth factor 2, *HIF-1α* hypoxia-inducible factor 1-alpha, *MAPK* mitogen-activated protein kinase, *RVF* right ventricular failure, *NF-κB* nuclear factor kappa-light-chain-enhancer of activated B cells, *MCT* monocrotaline, *PPHN* persistent pulmonary hypertension of the newborn, *AMPK* AMP-activated protein kinase, *EndMT* endothelial-to-mesenchymal transition

In addition to interacting with BMPR2 and TGF-β signaling pathways, PI3K/Akt can also crosstalk with NLRP3, MAPK, Notch, NF-κB and HIF-1α to promote the pathologic development of PH including cell proliferation and inflammation (Fig. 9a).

#### PAECs

In PAECs from IPAH patients and PH animal models, increased Ca^2+^ influx results in Ca^2+^-dependent phosphorylation of Akt and ERK, which subsequently upregulates the expression of Notch ligands Jagged1/2 and DLL4, contributing to pulmonary vascular remodeling associated with PAH and PH.^373^

#### PASMCs

KIF23 increases pyroptosis and proliferation in PASMCs of IPAH, hypoxia-PH, and MCT-PAH models, by increasing the expression of Caspase-3, NLRP3, and HMGB1 through the activation of PI3K/Akt and MAPK pathways. In PASMCs under hypoxia or PDGF treatment, activated ERK/Akt pathway upregulates HIF-1α expression.^368^ In PASMCs from PAH patients and 5-HT-treated bovine PASMCs, the JNK pathway is activated, driving cell proliferation through Akt activation. Concurrently, Akt activation enhances aerobic glycolysis and glycolysis-dependent lipogenesis.^180,374,375^ In vitro stimulation of bovine PASMCs with 5-HT demonstrated that activated JNK enhances Cyclin D expression by activating the Akt pathway, ultimately leading to increased cell proliferation.^374^ JNK plays a central role in metabolic reprogramming in PASMCs affected by PAH. In PAH patients, SIRT7 is upregulated in PASMCs, where it activates Akt through JNK.^375^ Akt activation then stimulates key adipogenic enzymes, including ACL and ACC, promoting glycolysis-dependent adipogenesis, which supports cell proliferation and survival.^375^ Additionally, Akt is involved in PDGF-induced aerobic glycolysis in PASMCs.^180^ Hypoxia triggers Akt phosphorylation, which enhances HIF-1α expression. This leads to the degradation of p27, further promoting PASMC proliferation.^376^

#### Macrophages

While the pro-inflammatory role of p38 is well-established, some early studies have observed its unexplained and contradictory anti-inflammatory effects.^377^ For instance, Shin et al. discovered that p38 negatively regulates NLRP3 by inhibiting Ca²⁺ mobilization. In p38-deficient mice, inflammasome hyperactivity resulted in pulmonary inflammation and heightened susceptibility to septic shock.^378^ This NLRP3 hyperactivity in p38-deficient macrophages was linked to Caspase-1 hyper-activation and an increased rate of pyroptosis.^378^

### NF-κB crosstalk with HIF-1α, and MAPK

NF-κB, a canonical signaling pathway integral to inflammatory responses, not only interacts with BMPR2, TGF-β, and the PI3K/Akt pathway but also demonstrates crosstalk with HIF-1α and MAPK, as supported by a substantial body of evidence (Fig. 9b).

#### NF-κB crosstalk with HIF-1α in PAECs

In PAECs exposed to hypoxia, increased Cul-5 promotes NF-κB activation by ubiquitinating TRAF6. This, in turn, enhances HIF-1α expression, ultimately fostering angiogenesis and improving PAEC adhesion by upregulating VEGF expression.^14^

Under hypoxic conditions, upregulation of CBLN2 in human PAECs boosts HIF-1α levels by promoting NF-κB expression. This increase in HIF-1α elevates Twist1, leading to a reduction in CD31 and an increase in α-SMA, ultimately driving EndMT.^379^

#### NF-κB crosstalk with HIF-1α in PASMCs

Under hypoxic conditions, PASMCs show significant interaction between HIF-1α and NF-κB. Hypoxia downregulates NLRC3 in PASMCs, which activates the IKK/NF-κB p65/HIF-1α pathway, leading to increased cell proliferation and inflammatory responses.^205^ Moreover, under hypoxic conditions, increased HIF-1α directly binds to the CD146 promoter, upregulating CD146 expression. The accumulation and dimerization of CD146 then activate NF-κB, enhancing HIF-1α transcription. This feedback loop promotes cell proliferation, migration, and ECM remodeling by increasing the expression of COL1, FN1, and vimentin.^121^

#### NF-κB crosstalk with MAPK in PAECs

In hypoxia-induced human PAECs, increased expression of p38 and MK2 leads to elevated NF-κB levels and pro-inflammatory cytokines, such as TNF and IL-6. This also promotes the expression of BET proteins (bromodomain-containing protein 2 and bromodomain-containing protein 4) and phosphorylation of STAT3, while reducing anti-inflammatory cytokines like IL-10.^169^ Additionally, human endogenous retrovirus K dUTPase activates the p38/NF-κB signaling pathway in PAECs, driving EndMT and inflammation.^380^

#### NF-κB crosstalk with MAPK in PASMCs

In FGF2-induced rat PASMCs, phosphorylated ERK activates the NF-κB pathway, leading to the upregulation of MCP-1 and PAI-1, which promotes inflammation and cell proliferation.^212^ Elevated MCP-1 further activates the ERK pathway, establishing a positive feedback loop that amplifies the inflammatory response.^212^

#### NF-κB crosstalk with MAPK in RVFs

Periostin, an ECM protein involved in tissue remodeling after injury, is upregulated in fibroblasts within the RVFs of PAH model rats. This upregulation is driven by the activation of the AP-1 pathway and an independent NF-κB pathway, which subsequently enhances the expression of iNOS through activation of the ERK1/2 and JNK pathways.^381^ The medium derived from periostin-induced RVFs inhibits L-type Ca²⁺ channel activity in CMs through NO production.^381^ This implies that NO generation involving the MAPK pathway may contribute to contractile dysfunction in RV failure associated with PAH. In the RVFs of MCT-PAH rats, the activation of ERK1/2 and JNK pathway upregulated NF-κB pathway, leading to increased iNOS expression and contractile dysfunction.^381^

### AMPK crosstalk with NF-κB, NLRP3, Akt/mTOR, and Notch

The activation of AMPK has emerged as a central regulator in mitigating PH by balancing cellular metabolism and energy homeostasis. AMPK activation has the potential to reduce inflammation, fibrosis, and vascular remodeling, acting as a counterbalance to the excessive metabolic and cellular dysfunction associated with PH through inhibiting key signaling pathways such as NF-κB, NLRP3, Akt/mTOR, and Notch (Fig. 9c).

#### PAECs

In PAECs from lambs with PPHN, reduced AMPK activity causes mitochondrial dysfunction and disrupts the balance of Notch ligands, resulting in increased DLL4 and decreased Jagged, which hinders angiogenesis. Treatment with AMPK agonists, like metformin, restored mitochondrial function and enhanced angiogenesis by normalizing the expression of these Notch ligands, thereby promoting a more favorable angiogenic response.^250^

#### PASMCs

Hypoxia-PH inhibits AMPK causing NF-κB/NLRP3-mediated PASMC inflammation, pyroptosis, and proliferation, ultimately causing pulmonary vascular remodeling.^382^ Activation of AMPK prevents MCT-PAH in rats by suppression of NF-κB-mediated autophagy activation.^383^ Upregulation of α-enolase levels during hypoxia-PH promotes PASMC proliferation via AMPK-Akt activation.^253^

### CaSR crosstalk with other signaling pathways

The interaction between CaSR and other signaling pathways further complicates the regulation of PASMC proliferation in PH. Notch signaling, for example, enhances CaSR expression and function in hypoxic PASMCs, leading to increased cytosolic Ca²⁺ levels and promoting PASMC proliferation. Inhibition of Notch3 or treatment with DAPT (a selective inhibitor of γ-secretase) in experimental models of hypoxia-PH attenuates these effects, highlighting the contribution of Notch-CaSR signaling to PH development.^384^ Moreover, the involvement of CaSR in vascular remodeling is further supported by its interaction with PDGF signaling, where PDGF upregulates CaSR expression in PASMCs, driving excessive proliferation and vascular remodeling in IPAH. Inhibition of PDGF or CaSR effectively reduces progression in IPAH models, suggesting that the CaSR-PDGF signaling axis plays a pivotal role in disease pathogenesis.^385^ The complex interplay between CaSR and various signaling pathways underscores the importance of CaSR in PH pathogenesis and presents numerous opportunities for therapeutic intervention.

#### Clinical and pre-clinical therapeutic strategies targeting signaling pathways

Pre-clinical and clinical studies are currently investigating the targeting of signaling pathways, given their role in PH. Pre-clinical research has demonstrated promising results, and several drugs targeting specific signaling pathways have shown significant efficacy in clinical trials. As a result, targeting these pathways offers important insights for developing therapeutic strategies for PH.

#### Targeting BMPR2 signaling pathway

Based on the critical role of BMPR2 deficiency in the onset and progression of PH, directly upregulating BMPR2 seems to exert beneficial effects on the disease (Table 1). For instance, Reynolds and colleagues developed adenovirus vectors containing the *BMPR2* gene, which targeted pulmonary vascular endothelium and ameliorated pulmonary hemodynamics and RVH in hypoxia-PH, and MCT-PAH rats.^386,387^ These findings suggest the great potential of gene therapy, regardless of whether PH is associated with suppression of BMPR2 levels or a mutation. As vector technology advanced, adeno-associated virus (AAV) vectors demonstrated improved transduction efficiency and reduced immunogenicity.^41^ AAV1.hSIN3a in MCT-PAH rats and Su/Hx-PAH mice reversed hypermethylation of the BMPR2 promoter region and repression of BMPR2, showing attenuated pulmonary vascular remodeling.^41^

**Table 1:** The therapeutic strategies targeting signaling pathways for PH

THERAPEUTIC	INTERVENTION	TARGET	DISEASE	Group	EFFECT		REFERENCE
STRATEGY		PATHWAY	(MODELS)	(PH)	Hemodynamic	Pathological	
**Cell therapy**	Preconditioned MSCs with PGE1	Activates HIF-1α	MCT-PAH rat	Group 1	Decreases mPAP	Inhibits MSC apoptosis and promotes the migration of MSCs to the injury site	^413^
	Medium from M2b macrophages	Inhibits PI3K/Akt	MCT-PAH rat	Group 1	/	Promotes apoptosis, inhibits PASMC proliferation and migration	^182^
	Modified SCs to overproduce FGF2	Activates PI3K/Akt	MCT-PAH rat, MCTP-human PAECs	Group 1	Decreases RVSP and RVHI	Inhibits apoptosis, promotes PAEC proliferation and viability	^424^
**Gene therapy**	AAV1.hSIN3a	Increases BMPR2	MCT-PAH rat, Su/Hx-PAH mouse	Group 1	Decreases mPAP, and RVSP; increases CO	Inhibits PASMC proliferation and migration, attenuates pulmonary vascular remodeling and RVH	^41^
	*Il6* knockout	Increases BMPR2	Bleomycin-PH mouse	Group 3	Decreases RVSP	Attenuates pulmonary fibrosis, pulmonary vascular thickening, and RVH	^395^
	*Inhba* knockout	Stabilizes BMPR2	hypoxia-PH mouse	Group 3	Decreases RVSP	Inhibits PAEC apoptosis and improves tube information; attenuates pulmonary vascular remodeling and RVH	^30^
	*Ctsl* knockdown	Stabilizes BMPR2	MCT-PAH and Su/Hx-PAH rat	Group 1	Decreases RVSP	Inhibits PAEC pyroptosis, attenuates pulmonary vascular remodeling and RVH	^64^
	*CYP2J2* overexpression	Inhibits TGF-β/Smad2	MCT-PAH rat	Group 1	/	Attenuates RVH	^401^
	*Capns1* knockout	Inhibits TGF-β/Smad2	hypoxia-PH mouse	Group 3	Decreases RVSP and RVHI	Reduces medial thickness of the pulmonary vascular wall	^80^
	*CPS1-IT* overexpression	Inhibits HIF-1α	OSA rat	Group 3	Decreases mRVP, mPAP	Inhibits excessive proliferation of vascular cells and inflammation	^513^
	*HIF1A* overexpression	Activates HIF-1α	PASMCs from IPAH patients	Group 1	/	Blocks the contraction of PASMCs	^112^
	*CMG2* overexpression	Activates PI3K/Akt	SPS-PAH rat, human PASMCs, and human PAECs with CMG2 knockdown	Group 5	Decreases mPAP, PASP, and RVSP	Inhibits PASMC proliferation	^425^
	Adenovirus-mediated *A20*	Inhibits NF-κB	hypoxia-PH mouse, hypoxic rat PASMCs	Group 1	Reduces RVSP	Inhibits PASMC proliferation, attenuates pulmonary vascular remodeling and RVH	^436^
	*Nfkbia* mutant plasmid	Inhibits NF-κB	MCT-PAH rat	Group 1	Decreases RVSP	Restores endothelial cell function by inhibiting apoptosis and reversing EndMT	^356^
	*Nkd1* overexpression	Inhibits Wnt/β-catenin	MCT-PAH mouse, hypoxic mouse PASMCs	Group 1	Decreases RVSP	Inhibits PASMC proliferation and migration and attenuates RVH	^444^
	si-circ_0016070	Inhibits TCF4/β-catenin	MCT-PAH rat, hypoxia-PH rat	Group 1, 3	Decreases RVSP and RVHI	Attenuates pulmonary vascular remodeling	^278^
**Medication**	FK506	Activates BMPR2	PAH patients, MCT-PAH, Su/Hx-PAH rat, *BMPR2* mutant PMVECs from IPAH patients	Group 1	Decreases RVSP	Increases angiogenesis and attenuates RVH	^388,390,391^
	Seralutinib	inhibit PDGFR, increase BMPR2	PAH patients; Su/Hx-PAH rat, MCT-PAH rat	Group 1	Decreases PASP and RVSP	Inhibits proliferation of PAECs, PASMCs, and lung fibroblasts, and attenuates pulmonary artery muscularization and RVH	^389,392^
	Enzastaurin	Increase FHIT/BMPR2	Su/Hx-PAH rat; hypoxia-PH mouse with Fhit-/- or *Bmpr2*^*+/-*^	Group 1, 3	Decreases RVSP	Attenuates DNA damage, decreases apoptosis, restores tube formation of PAECs, attenuates RVH, cardiac fibrosis, and vascular remodeling	^31^
	BMP9	Activates BMPR2/Smad1/5	Spontaneous PH mouse, MCT-PAH, Su/Hx-PAH rat	Group 1	Decreases RVSP	Inhibits PAEC apoptosis, enhances monolayer integrity, and attenuates pulmonary arterial muscularization	^39^
	RhBMP9	Activates BMPR2/Smad1/5/9	Bleomycin-PH rat	Group 3	Decreases RVSP	Inhibits PMVEC apoptosis, interstitial collagen deposition, and media thickening	^393^
	Soluble GP130	Increases BMPR2	Bleomycin-PH mouse	Group 3	Decreases RVSP	Attenuates pulmonary fibrosis, pulmonary vascular thickening, and RVH	^395^
	DHEA	Activates BMPR2, inhibits RhoA/ROCK	MCT-PAH rat	Group 1	Decreases PAP	Inhibits proliferation, increases apoptosis of PASMCs, and attenuates RVH	^68,290^
	Isorhamnetin	Activates BMPR2/Smad1/5	MCT-PAH rat	Group 1	Decreases mPAP, and RVSP	Prevents PAEC injury, inhibits PASMC proliferation and hypertrophy,	^396^
	Elafin	Stabilizes BMPR2	Su/Hx-PAH rat, PAECs from PAH patients	Group 1	Decreases RVSP; increases CO	Improves PAEC survival and angiogenesis, inhibits PASMC proliferation and anti-apoptosis, attenuates pulmonary vascular remodeling and RVH	^394^
	Follistatin	Stabilizes BMPR2	Conditioned medium (derived from IPAH-ECs) induced PAECs	Group 1	Decreases RVSP	Inhibits PAEC apoptosis and improves tube information; attenuates pulmonary vascular remodeling and RVH	^30^
	Sotatercept	Inhibits TGF-β/Smad2/3	PAH patients, MCT-PAH, Su/Hx-PAH rat	Group 1	Decreases PVR and RVSP	Reduces inflammation, inhibits PASMC and PMVEC proliferation, attenuates arteriolar muscularization	^399,400^
	IN-1233	Inhibits TGF-β	MCT-PAH rat, TGF-β-rat PASMCs	Group 1	Decreases RVSP	Inhibits PASMC migration and prevents RVH	^77,402^
	SB525334	Inhibits TGF-β	MCT-PAH rat	Group 1	Decreases RVP	Inhibits RVH, and reverses pulmonary artery muscularization	^403^
	SD-208	Inhibits TGF-β	MCT-PAH rat	Group 1	Decreases RVSP, RVDP and increases CO	Inhibits PASMC proliferation	^404^
	Aspirin	Inhibits HIF-1α/TGF-β	hypoxia-PH rat	Group 3	/	Reduces ECM remodeling	^363^
	Berberine	Inhibits TGF-β	hypoxia-PH mouse	Group 3	Decreases RVSP	Inhibits PASMC proliferation and reduces pulmonary artery muscularization	^407^
	Danshensu	Inhibits TGF-β	hypoxia-PH rat	Group 3	Decreases RVSP and RVHI	Inhibits PASMC proliferation	^406^
	Ginsenoside Rg1	Inhibits NF-κB/TGF-β	hypoxia-PH rat	Group 3	Decreases RVSP and mPAP	Reduces inflammatory factors and prevents pulmonary vascular remodeling	^405^
	Pioglitazone	Inhibits TGF-β	LPR1^-/-^ -IPAH mouse, PASMCs from PAH patients	Group 1	Decreases RVSP, RVEDP, LVSP and LVEDP	Inhibits PASMC proliferation and pulmonary vascular remodeling, and reverses RVH	^408^
	C76	Inhibits HIF-2α	Egln1^-/-^ mouse, Su/Hx-PAH rat, MCT-PAH rat	Group 1	Decreases RVSP	Attenuates pulmonary vascular remodeling	^412^
	PT2567	Inhibits HIF-2α	Su/Hx-PAH rat, hypoxia-PH rat	Group 1, 3	Decreases PVR and mPAP	Decreases inflammation, proliferation, and vascular remodeling	^128,411^
	Caffeic Acid Phenethyl Ester	Inhibits HIF-1α	MCT-PAH rat	Group 1	Decreases RVSP	Inhibits proliferation and promotes apoptosis	^368^
	2-Methoxyestradiol	Inhibits HIF-1α	hypoxia-PH in OVX rat, hypoxia- human PASMCs	Group 3	Decreases mPAP	Prevents mitochondrial ultrastructure damage and alleviates oxidative stress	^514,515^
	Digoxin	Inhibits HIF-1α	hypoxia-PH mouse	Group 3	Decreases RVSP	Reduces pulmonary vascular remodeling	^410^
	Luteolin	Inhibits HIF-2α and PI3K/Akt	hypoxia-PH rat and MCT-PAH rat	Group 1, 3	Decreases mPAP	Inhibits PASMC proliferation and migration, pulmonary fibrosis, and pulmonary vascular remodeling, improves endothelial function	^177^
	anti-CD146 antibody	Inhibits HIF-1α	MCT-PAH rat	Group 1	Decreases RVSP	Attenuates pulmonary vascular remodeling	^121^
	Baicalin	Inhibits HIF-1α and Akt	hypoxia-PH rat	Group 3	Decreases RVSP	Inhibits PASMC proliferation	^376^
	Apigenin	Inhibits HIF-1α	hypoxia-PH rat	Group 3	Decreases RVSP	Promotes apoptosis	^133^
	PT2385	Inhibits HIF -2α	Su/Hx-PAH rat	Group 1	Decreases RVSP	Prevents PAEC apoptosis and attenuates RVH	^120^
	Dichloroacetate	Inhibits HIF-1α	hypoxic human PASMCs	Group 3	/	Increases apoptosis and inhibits proliferation	^409^
	3PO	Inhibits ERK	MCT-PAH rats	Group 1	Improves PAAT and CO, decreases RVSP	Improves right ventricular function, attenuates collagen synthesis and PASMC proliferation	^159^
	SB203580 and PH-797804	Inhibits p38	MCT-PAH rat, hypoxia-PH rat	Group 1, 3	Decreases RVSP	Attenuates pulmonary vascular remodeling and RVH	^168^
	Astragaloside IV	Inhibits ERK	MCT-PAH rat, hypoxic human PASMCs and PAECs	Group 1, 3	Decreases RVSP	Decreases inflammation and prevents proliferation and dysfunction	^516^
	Paeoniflorin	Inhibits p38/ERK	MCT-PAH rat	Group 1	Decreases RVSP	Reduces inflammation and EndMT, and attenuates pulmonary vascular and RV remodeling	^414^
	GS-444217	Inhibits p38/JNK	MCT-PAH rat, Su/Hx-PAH rat	Group 1	Decreases mPAP	Attenuates pulmonary vascular remodeling and RVH	^167^
	Baicalin	Inhibits PI3K/Akt	hypoxia-PH rat	Group 3	Decreases RVSP	Attenuates pulmonary arterial remodeling and RVH	^417^
	Ligustrazine	Inhibits PI3K/Akt	MCT-PAH rat	Group 1	Decreases RVSP	Inhibits PASMC proliferation and inflammation, and attenuates pulmonary vascular remodeling and RVH	^418^
	3-Bromopyruvate	Inhibits PI3K/Akt	MCT-PAH rat	Group 1	Increases TAPSE, decreases RVHI	Inhibits PASMC proliferation and pulmonary vascular remodeling, preserves mitochondrial morphology, but causes ascites and organ cirrhosis.	^194^
	Dacomitinib	Inhibits PI3K/Akt	MCT-PAH rat	Group 1	Decreases RVSP	Inhibits PASMC proliferation, migration, and autophagy, reduces pulmonary vascular remodeling, and prevents RVH	^419^
	Spermine	Inhibits PI3K/Akt and ERK	hypoxic human PASMCs	Group 3	/	Causes G1/G0 cell cycle arrest and inhibits PASMC proliferation	^420^
	NPS2390	Inhibits PI3K/Akt	hypoxic human PASMCs	Group 3	/	Inhibits autophagy and proliferation of PASMCs, while promoting a shift from a synthetic to a contractile phenotype	^421^
	Resveratrol	Inhibits PI3K/Akt and NF-κB	hypoxia-PH rat, MCT-PAH rats, hypoxic human PASMCs	Group 1, 3	Decreases RVSP	Attenuates PASMC proliferation, and RVH	^423^
	Genistein	Activates PI3K/Akt	hypoxia-PH and MCT-PAH rat; hypoxic PAECs (Broiler chickens)	Group 1, 3	Decreases PAP	Restores endothelial function, reduces RVH and increases survival rates	^185,186,416^
	Tanshinone IIA	Activates PI3K/Akt	MCT-PAH rat	Group 1	Decreases PAP	Attenuates pulmonary artery remodeling	^415^
	17β-estradiol	Activates PI3K/Akt	MCT-PAH in OVX rat	Group 1	Decreases RVSP, mPAP	Enhances apoptosis, reduces PASMC proliferation, and attenuates RVH	^184^
	Simvastatin	Inhibits NF-κB	MCT-PAH rat, PDGF-rat PVSMCs	Group 1	Decreases mPAP	Enhances PVSMC apoptosis and reduces endomembrane proliferation in pneumono-arteriole	^428^
	Atorvastatin	Inhibits NF-κB	CRP-human PASMCs	Group 1	/	Alleviates inflammation in human PASMCs.	^429^
	Prednisolone	Inhibits NF-κB	PDGF-human PASMCs	Group 1	/	Exerts anti-inflammatory and antiproliferative effects on PASMCs	^430^
	Nicorandil	Inhibits NF-κB	hypoxic human PAECs	Group 3	/	Inhibits PAEC apoptosis	^431^
	Treprostinil	Inhibits NF-κB	Inflammatory cytokines-induced human alveolar macrophages	Group 1	/	Inhibits secretion and gene expression of inflammatory cytokines	^432^
	Pyrrolidine dithiocarbamate	Inhibits NF-κB	MCT-PAH rat, CRP-human PAECs	Group 1, 3	Decreases PAP	Reduces macrophage infiltration and inflammation in PAECs, and prevents RVH	^27,426,517^
	IMD-0354	Inhibits NF-κB	MCT-PAH rat	Group 1	Decreases RVSP	Suppresses PASMC proliferation and induces PASMC apoptosis	^212^
	HGF	Inhibits NF-κB	MCT-PAH rat	Group 1	Decreases mPAP and RVHI	Reduces inflammation	^215^
	Hydrogen sulfide	Inhibits NF-κB	MCT-PAH rat, TGF-β-human PAECs	Group 1	Decreases RVSP	Inhibits EndMT and attenuates RVH	^433,434^
	Paeoniflorin	Inhibits NF-κB and MAPK	MCT-PAH rat, TGF-β-human PAECs, PDGF-B-human PASMCs	Group 1	Decreases RVSP	Partially reverses EndMT, reduces right ventricular pressure and remodeling, and attenuates RVH	^414^
	Alginate oligosaccharides	Inhibits NF-κB and p38	MCT-PAH rat	Group 1	Decreases RVHI	Attenuates pulmonary arteriolar remodeling	^518^
	Enalapril	Inhibits NF-κB	Bleomycin-PH mouse	Group 3	Decreases PAP	Reduces lung injury	^438^
	MnTE-2-PyP	Inhibits NLRP3	hypoxia-PH mouse	Group 3	Decreases RVSP	Decreases inflammation and attenuates vascular remodeling	^218^
	Jiedu Quyu Decoction	Inhibits NLRP3	MCT-PAH rat	Group 1	Improves PAT and CO	Mitigates right-sided heart failure associated with PAH	^223^
	Sulforaphane	Inhibits NLRP3	Su/Hx-PAH mouse	Group 1	Decreases RVHI, improves RV function	Prevents pulmonary vascular remodeling and RVH	^224^
	PNU-282987	Inhibits NLRP3	MCT-PAH rat	Group 1	Decreases RVSP	Attenuates fibrosis, RVH, and improves survival	^441^
	MCC950	Inhibits NLRP3	MCT-PAH rat	Group 1	/	Decreases apoptosis and inflammation	^440^
	Astragaloside IV	Inhibits NLRP3	MCT-PAH rat, MCTP-human PAECs	Group 1	/	Decreases apoptosis and inflammation in PAECs	^440^
	Ellagic Acid	Inhibits NLRP3	MCT-PAH rat	Group 1	Decreases RVSP and RVHI	Reduces oxidative stress and hypertrophy	^439^
	Adenoviral sJag1 transfection	Inhibits Notch1/3	hypoxia-PH mouse, MCT-PAH rat	Group 1, 3	Decreases mPAP and RVHI	Inhibits PASMC proliferation, enhances apoptosis, restores PASMC phenotype, improves survival rate	^235^
	Nitrite plus metformin	Activates AMPK	SU5416/Obese ZSF1 rat (PH-HFpEF)	Group 2	Decreases RVSP	Attenuates hyperglycemia, glucose intolerance, and vascular remodeling	^248^
	Metformin	Activates AMPK	Su/Hx-PAH rat, hypoxia-PH mouse, MCT-PAH rat, Group 3 PH patients	Group 1, 3	Decreases RVSP and RVHI	Attenuates collagen deposition, pulmonary vascular remodeling, RVH, phenylephrine-induced contractions, and endothelial function	^256,442^
	Treprostinil and metformin	Activates AMPK	Metabolic syndrome-associated PH-HFpEF mouse, SU5416/Obese ZSF1 rat	Group 2, 5	Increases PAAT/ET and TAPSE	Attenuates hyperglycemia, and glucose intolerance and improves cardiac function	^519^
	Ponatinib	Inhibits Wnt5a/β-catenin	Bleomycin-PH, hypoxic human PASMCs	Group 3	Decreases RVSP	Attenuates pulmonary vascular remodeling	^445^
	SBFI-26	Inhibits Wnt/β-catenin	PH-LHD mouse	Group 2	Decreases RVSP	Improves RV function and inhibits pulmonary vascular remodeling	^276^
	Doxycycline	Activates FGF2/FGFR	hypoxia-PH mouse	Group 3	Decreases RVP	Reduces EndMT and RVH	^283^
	Dovitinib	Inhibits FGF2/FGFR	MCT-PAH rat	Group 1	Decreases mPAP	Inhibits proliferation of PASMCs and Pulmonary ECs	^282^
	PD173074	Inhibits FGF2/FGFR	MCT-PAH	Group 1	Decreases mPAP, RVSP and PASP	Reduces RVH and proliferation of PASMCs	^446^
	Rosiglitazone	Activates PPARγ	SMYD2 overexpression in hypoxia-PH mouse and PASMCs	Group 3	Decreases RVSP	Decreases PASMC proliferation and attenuates RVH, and pulmonary vascular remodeling	^307^
	Sildenafil	Activates PPARγ	hypoxia-PH rat, hypoxic human PASMCs	Group 3	Decreases RV mean pressure	Inhibits proliferation and attenuates RVH	^475^
	Canagliflozin	Activates PPARγ	hypoxia-PH mouse, Su/Hx-PAH mouse and rat, MCT-PAH rat	Group 1, 3	Decreases RVSP, PAT, and PAT/PET	Inhibits excessive oxidative stress and proliferation, and attenuates pulmonary vascular remodeling	^520^
	Fasudil	Inhibits RhoA/ROCK	hypoxia-PH rat	Group 3	Decreases mPAP and RVSP	Decreases the migration of human PMVECs and attenuates pulmonary vascular remodeling	^455^
	KMUP-1	Inhibits RhoA/ROCK	U46619-PAH, MCT-PAH rat	Group 1	Decreases mPAP	Decreases vascular remodeling and vasoconstriction	^455^
	G1	Activates GPER	MCT-PAH rat	Group 1	Decreases LVCO, RVCO, and RVSP	Promotes pulmonary endothelial NO synthesis, Ca^2+^ handling regulation, reduces inflammation in CMs, and collagen deposition	^311^
	NPS2143	Inhibits CaSR	MCT-PAH rat, hypoxia-PH mouse	Group 1, 3	Decreases RVSP	Attenuates myocardial fibrosis and RVH	^320^
	Docosahexaenoic acid	Inhibits CaSR	hypoxia-PASMCs	Group 1	/	Reduces the proliferation and migration of PASMCs	^487^
	Chloroquine	Inhibits CaSR	hypoxia-PH rat	Group 3	/	Attenuate arteriole thickness	^488^
	NPS2143/sildenafil	Inhibits CaSR	IPAH-PASMCs	Group 1	/	Reduces proliferation of PASMCs	^489^
	Vitamin K3, MG-132	Inhibits Hippo	MCT-PAH rat	Group 1	Decreases RVSP, RVHI	Decreases PASMC proliferation and anti-apoptosis, alleviates pulmonary arterial remodeling	^327^
	Cinnamaldehyde	Inhibits cGAS-STING	MCT-PAH rat	Group 1	Decreases RVOT, increases PATET	Decreases PASMC proliferation and inflammatory responses, alleviates mitochondrial damage and vascular remodeling	^348^
	SITO	Inhibits cGAS-STING	MCT-PAH rat	Group 1	Decreases RVSP	Inhibits proliferation and promotes apoptosis of PASMCs, mitigates phenotypic switching	^349^

*MCT-PAH* monocrotaline induced pulmonary arterial hypertension, *CTEPH* chronic thromboembolic pulmonary hypertension, *IPAH* Idiopathic pulmonary arterial hypertension, *Su/Hx-PAH* SU5416/Hypoxia-induced pulmonary arterial hypertension, *PH-LHD* pulmonary hypertension secondary to left-sided heart disease, *SPS-PAH* systemic-to-pulmonary shunt-induced pulmonary arterial hypertension, *PNT/MCT-PAH* pulmonary arterial hypertension induced by unilateral left pneumonectomy combined with monocrotaline, *PASMCs* pulmonary artery smooth muscle cells, *mPAP* mean pulmonary artery hypertension, *dPAP* diastolic pulmonary artery pressure, *RVSP* right ventricular systolic pressure, *PAT* pulse arrival time, *PAT/PET* pulmonary artery acceleration time to ejection time, *LVCO* left ventricular cardiac output, RVCO right ventricular cardiac output, *RVHI* right ventricular hypertrophic index (RV/(LV + S)), *RVOT* right ventricular outflow tract obstruction, *PATET* pulmonary artery acceleration time to ejection time ratio, *TAPSE* tricuspid annular plane systolic excursion, *PAAT* pulmonary artery acceleration time, *PVR* pulmonary vascular resistance, *PASP* pulmonary artery systolic pressure, *RVEDP* right ventricular end-diastolic pressure, *LVSP* left ventricular systolic pressure and *LVEDP* left ventricular end-diastolic pressure

Although gene delivery still has a long way to go for clinical application, upregulating BMPR2 via medication manifests more development. In vitro *and* in vivo studies demonstrate the efficacy of FK506 (tacrolimus) and Seralutinib in improving PAH by increasing the expression of BMPR2 and activating its downstream signaling.^388,389^ Clinical trials have confirmed the safety of FK506 and Seralutinib in PAH patients, with an increase in BMPR2 expression observed. However, no significant changes in related signaling were noted.^390–392^ Additionally, enzastaurin upregulates BMPR2 by increasing FHIT, ameliorating hypoxia-PH in mice and Su/Hx-PAH in rats.^31^

Other studies suggest targeting BMPR2 signaling could benefit PH treatment, with BMP9 directly activating the BMPR2 pathway.^39,393^ Elafin, an endogenous elastase inhibitor augments BMPR2 signaling by enhancing the interaction between BMPR2 and CAV1.^394^ High inhibin-β-A expression in IPAH patients promotes autocrine ActA secretion, while follistatin stabilizes BMPR2.^30^ Inhibition of IL-6/STAT3 signaling may enhance BMPR2 expression^,^^68,395^. Cathepsin L inhibition by short hairpin RNA (shRNA) can attenuate PAH in MCT- and Su/Hx-induced rats by restoring BMPR2 expression.^64^ Isorhamnetin reduces inflammatory factors and improves BMPR2 signaling in MCT-PAH rats.^396^ Overall, normal BMPR2 expression is vital for pulmonary vascular health, and its deficiency promotes PH progression through inflammation and cellular changes. Investigating the interplay between BMPR2 and inflammatory responses may lead to targeted therapeutic strategies for PH.

#### Targeting TGF-β signaling pathways

Sotatercept and its rodent analog, RAP-011, are ActRIIA-Fc fusion proteins that neutralize activin-class ligands (activins A/B, GDF8, GDF11) elevated in PAH.^73,397^ By modulating Smad2/3 signaling, they exert anti-proliferative, pro-apoptotic, and anti-inflammation effects in cellular and animal PAH models.^73,397,398^ In MCT-PAH and Su/Hx-PAH rat models, it reduces mPAP and pulmonary arterial remodeling without impacting systemic arterial pressure^89^ (Table 1). Additionally, recent Phase II and III trials (NCT03738150, NCT04576988, NCT04811092, NCT04896008) results indicate that patients with PAH receiving sotatercept showed a greater improvement in exercise capacity, as measured by the 6-minute walk test, compared to those receiving a placebo while on stable background therapy.^399,400^

Other pre-clinical studies have demonstrated the potential value of targeting the TGF-β pathway to treat PAH. For example, injection of a plasmid containing the *CYP2J2* gene into MCT-PAH rats to over-express CYP2J2 inhibits the TGF-β/Smad2 pathway, downregulates the expression of IL-6 and IL-10, and upregulates the activity of eNOS and NOS, thereby ameliorating inflammation and RVH.^401^ Inhibition of ALK5 using IN-1233, SB525334, or SD-208 lowers Smad2 phosphorylation levels, leading to reduced migration of PASMCs, decreased RVSP, and alleviated RVH and peripheral arterial muscularization induced by MCT.^402–404^ Knockdown of *Calpain-4* inhibits Smad2/3 phosphorylation, which leads to reduced COL1 accumulation in the lungs of hypoxic mice, thereby preventing the progression of pulmonary vascular remodeling.^80^ Aspirin or *ginsenoside Rg1* inhibits hypoxia-induced EndMT by inhibiting the TGF-β/Smad2/3 signaling pathway, resulting in downregulation of VE-cadherin, α-SMA, and vimentin expression.^363,405^ Treatment with danshensu or berberine inhibited the TGF-β/Smad2 signaling pathway in hypoxia-PH rats. This led to reduced expression of PCNA and decreased proliferation of PASMCs. Additionally, berberine treatment activated the BMPR2/Smad1/5 signaling pathway.^406,407^ LRP1 inhibits TGF-β-CTGF signaling. In LRP1-deficient mice with IPAH and PASMCs from IPAH patients, treatment with pioglitazone activates PPARγ, allowing it to bind to Smad2 and compensate for the lack of LRP1^408^ (Table 1).

Collectively, excessive activation of the TGF-β signaling pathway promotes the development of PH. A deeper understanding of the relationship between TGF-β and PH suggests that therapeutic strategies aimed at inhibiting the TGF-β pathway may be highly effective in improving PH.

#### Targeting HIF signaling pathways

Relevant studies indicate that therapeutic effects can be achieved at the gene level through methods like transfection of siRNA targeting *Hif-1*α and specific knockout of *Hif-1*α (Table 1). Additionally, various inhibitors have been shown to be effective in treating hypoxia-PH. Caffeic Acid Phenethyl Ester,^368^ Dichloroacetate,^409^ and digoxin^410^ inhibit HIF-1α to reduce migration and proliferation of vascular cells, pulmonary vascular remodeling, RVSP, and RVH. PT2567,^128,411^ luteolin,^177^ PT2385,^120^ and C76^412^ have shown promise in inhibiting HIF-2α to reduce PVR, mPAP, inflammation, the proliferation of vascular cells, and vascular remodeling. In summary, targeting *Hif-1*α or *Hif-2*α inhibition in PH often achieves therapeutic objectives. However, some studies indicate that the protein expression of HIF-1α in PASMCs of IPAH patients is decreased, leading to an increase in contractile force.^112,113^ Conversely, transfecting *Hif-1*α can reduce this contractile force,^112^ highlighting the need to consider the specific type of PH when implementing HIF-targeted therapies. Additionally, the transplantation of mesenchymal stem cells into rats with MCT-PAH, followed by stimulation with prostaglandin E1, resulted in increased expression of HIF-1α.^413^ This upregulation enhanced the migration of mesenchymal stem cells to the injury site by increasing SDF-1α/CXCR4 expression, ultimately alleviating PH.^413^ These findings suggest that HIF-1α may play varying roles in stem cells, indicating that stem cell therapy could emerge as a promising approach for treating PH and provide a new direction for future research.

#### Targeting MAPK signaling pathways

Many existing drugs that target the MAPK signaling pathway may have potential applications in PH (Table 1). *Paeoniflorin*, a monoterpene glycoside, offers various health benefits, including vasodilation, anti-inflammatory effects, and immunomodulation. *Paeoniflorin* increases BMPR2 in MCT-PAH rats and reduces the phosphorylation of TAK1 in vivo.^414^ This action suppresses the MAPK/NF-κB signaling pathways, suggesting that *Paeoniflorin* may serve as a potential therapeutic agent for PH.^414^ Moreover, inhibitors targeting molecules upstream of the MAPK pathway may similarly alleviate PH. The PFKFB3 inhibitor 3-(3-pyridinyl)-1-(4-pyridinyl)-2-propen-1-one diminished the activation of the ERK pathway by downregulating lactate levels, and subsequently attenuated the increased spectrin breakdown product levels in the PASMCs of hypoxic mice and MCT-PAH rats.^159^ The ASK1 inhibitor GS-444217 was tested in MCT- and Su/Hx-PAH rats. This inhibitor effectively reduced JNK/p38 activation, leading to decreased PAP and RVH.^167^ In summary, inhibiting the aberrantly activated MAPK pathway can slow PH progression. Additionally, some existing anti-inflammatory medications may be valuable in PH treatment strategies.

#### Targeting PI3K/Akt signaling pathway

In pre-clinical studies, phosphatase and tension homolog is acknowledged as a negative regulator of the PI3K/Akt pathway in PH treatment^190^ (Table 1). It accomplishes this by dephosphorylating PIP3 to PIP2, influencing key cellular processes associated with the disease. Studies have shown that inhibition of TGF-β^365^ can modulate the PTEN/PI3K/Akt signaling, ultimately attenuating PH. Various therapeutic substances and methods have been investigated for their potential to modulate the PI3K/Akt signaling pathway, subsequently alleviating PH. Among pharmaceutical compounds, estradiol,^184^ tanshinone IIA,^415^ genistein,^185,186,416^ baicalin,^417^ ligustrazine,^418^ 3-bromopyruvate,^194^ dacomitinib,^419^ exogenous spermine,^420^ and NPS2390^421^ have been explored. Natural compounds such as luteolin,^177,422^ and resveratrol^423^ have also shown promise. Additionally, interventions involving cell and molecular approaches, including conditioned medium from M2b macrophages,^182^ FGF2 over-expression adipose-derived mesenchymal stem cells^424^ and genetic modulation of CMG2 have been explored.^425^

#### Targeting NF-κB signaling pathway

Various therapeutic substances have been explored in pre-clinical studies for their potential to modulate NF-κB signaling to alleviate PH (Table 1). NF-κB inhibitors, such as Pyrrolidine dithiocarbamate, N-(3,5-Bis-trifluoromethyl-phenyl)-5-chloro-2-hydroxy-benzamide, and BAY11-7082, along with gene therapy using the dominant-negative *I*κ*B*α triple mutant gene, have been demonstrated to mitigate PH in various models by preventing excessive NF-κB activation, reducing inflammation and PH-associated pathological processes.^200,212,356,426,427^ For instance, Pyrrolidine dithiocarbamate, has been shown to ameliorate MCT-PH in rats by preventing NF-κB nuclear localization and reducing VCAM-1 expression.^426^ Moreover, pharmacological compounds, such as Simvastatin,^428^ Atorvastatin,^429^ Prednisolone,^430^ Nicorandil,^431^ Treprostinil,^432^ Baicalein,^216^ exert their effects by suppressing NF-κB expression, preventing vascular remodeling and PH. Biological factors like Hydrogen sulfide,^433–435^ Hepatocyte Growth Factor,^215^ and Zinc finger protein A20^436^ reduce cell proliferation, inflammation, and disease severity by inhibiting NF-κB signaling. In the field of advanced delivery methods, nanoparticle-mediated delivery of NF-κB decoy into lungs has shown promise in ameliorating MCT-PAH.^204^ Among natural compounds, *Srolo Bzhtang*,^214^ *Paeoniflorin*,^414^ Resveratrol^437^ have demonstrated efficacy in attenuating PAH through modulation of the NF-κB signaling pathway. Traditional therapies like *Enalapril*^438^ protect against PAH by inhibiting TNF-mediated NF-κB and AP-1 activation.

#### Targeting NLRP3 signaling pathway

Several therapeutic substances have emerged as direct inhibitors of the NLRP3 inflammasome (Table 1), showing promise in mitigating pre-clinical models of MCT-PAH. Natural compounds including ellagic acid (a natural compound found in various fruits) and sulforaphane (a phytochemical that is found in cruciferous vegetables) are noteworthy examples. Ellagic acid exhibits preventive effects against MCT-PAH by specifically targeting and inhibiting NLRP3 inflammasome activation.^439^ Moreover, the protective effect of Sulforaphane on PAH via the prevention of RV injury and pulmonary vascular remodeling was associated with the reduction of the NLRP3 expression.^224^ Pharmacological interventions also play a crucial role in NLRP3 signaling modulation for PAH therapy. Additionally, the SOD mimetic, MnTE-2-PyP, emerges as a potent therapeutic agent, attenuating chronic hypoxia-PH, vascular remodeling, and NLRP3 inflammasome activation.^218^ Astragaloside IV, a natural compound, also contributes to this group, attenuating inflammatory response mediated by NLRP3/Calpain-1 and being involved in the development of PH.^440^ A novel complement C3 inhibitor, CP40-KK, has been demonstrated to protect against experimental PAH via an inflammasome NLRP3-associated pathway.^440^ According to Deng et al., the activation of Nicotinic Acetylcholine α7 Receptor with PNU-282987 attenuates the progression of MCT-PAH in rats by downregulating the NLRP3 Inflammasome.^441^

#### Targeting notch signaling pathway

In the field of therapeutic interventions targeting the Notch signaling pathway, soluble Jagged-1 stands out as a direct inhibitor with notable effects in inhibiting PH (Table 1). This soluble form of the Jagged-1 ligand acts by attenuating Notch signaling, thereby presenting a pharmacologic approach to mitigate PH.^235^

The direct targeting of Notch signaling showcases the potential of pharmacological interventions in modulating this pathway to alleviate PAH, emphasizing the importance of understanding the molecular mechanisms involved.

#### Targeting AMPK signaling pathway

Metformin, an established anti-diabetic medication, has gained attention for its potential therapeutic impact on PH by activating AMPK (Table 1). In preclinical studies, metformin emerges as a potent AMPK activator, providing protection against and reversing the development of PH.^256,442^ Its therapeutic effects during PH-induced RV dysfunction have been demonstrated to vary based on age and sex, and notably, these variations are observed independently of cardiac AMPK.^443^ Nitrite, in conjunction with metformin, activates AMPK through SIRT3, addressing hyperglycemia and normalizing PH associated with heart failure with preserved ejection fraction.^248^ The diverse therapeutic approaches targeting AMPK signaling, including both pharmacological and natural interventions, present a comprehensive strategy for combating PH and associated cardiovascular complications. Human evidence to preclinical studies of various interventions underscores the potential clinical significance of AMPK modulation in PH management.

#### Targeting Wnt signaling pathways

Wnt signaling is vital for regulating pulmonary angiogenesis and vascular remodeling, suggesting that therapies targeting Wnt pathway activity could benefit patients with PH (Table 1). NKD1 is downregulated in hypoxic mouse PASMCs. Introducing exogenous NKD1 can inhibit both β-catenin and ROS levels,^444^ suggesting its potential therapeutic effects in MCT-PAH mice. Kang et al. found that Ponatinib, a multi-target tyrosine kinase inhibitor, decreased the expression of Wnt5a, β-catenin, and Cyclin D in hypoxic human PASMCs, improving vascular remodeling and slowing disease progression in bleomycin-induced PAH rat models.^445^ Currently, most PH treatments targeting the Wnt signaling pathway focus on inhibiting the canonical pathway. However, due to the complexity of Wnt signal transduction, including its crosstalk with other pathways and the numerous signaling components involved, there is potential for broader therapeutic options within the Wnt pathway.

#### Targeting FGFR signaling pathway

Although a study suggests that activating endothelial FGFR signaling may offer therapeutic potential by inhibiting TGF-β-mediated EndMT in hypoxia-PH,^283^ a number of studies have demonstrated that inhibiting FGFRs, either through selective inhibitors or broader strategies, can attenuate the hyper-proliferation of PASMCs, prevent further vascular remodeling, and reduce hemodynamic impairments in experimental PAH and idiopathic PH models.^282,286,446^ FGFR inhibition in the MCT-PAH model reverses vascular remodeling, reduces pulmonary pressure, prevents RVH, and restores BMPR2 by suppressing ERK1/2 and Akt signaling.^446^ The ability of FGFR inhibitors to curb FGF2-driven proliferation and restore BMPR2 highlights the complex interplay between FGF2 and BMPR2 signaling in PAH pathogenesis. Over-activated p130^Cas^ signaling amplifies FGF2, EGF, and PDGF signaling in PAH, driving PASMC and PAEC proliferation and migration. Inhibition with gefitinib, dovitinib, and imatinib significantly attenuates PAH.^282^ Additionally, repeated FGF2-siRNA administration or FGFR1 inhibition with SU5402 effectively reverses idiopathic PH in rats by suppressing lung FGF2 production.^284^ A case report showed that anlotinib improved PH and respiratory dysfunction in pulmonary tumor thrombotic microangiopathy associated with gastric carcinoma by targeting VEGFR, FGFR, PDGFR, and c-kit.^447^ These findings suggest that targeting FGFR may restore vascular homeostasis and slow PH progression, making FGF2/FGFR signaling a key therapeutic focus.

#### Targeting RhoA/ROCK signaling pathway

Therapeutically, targeting the RhoA/ROCK pathway offers significant promise in managing PH. ROCK inhibitors, such as fasudil, ripasudil, KMUP-1, and SB-772077-B have been shown to improve pulmonary hemodynamics and reduce vascular remodeling in preclinical and clinical studies, highlighting their potential as a therapy for PH.^448–455^ Additionally, the modulation of RhoA/ROCK activity and signaling by substances such as dehydroepiandrosterone (DHEA),^290^ statins,^456,457^ heparin,^458^ fluoxetine^459^ and 18β-glycyrrhetinic acid^460^ has been shown to exhibit potential in the treatment of PH. These inhibitors and substances work by relaxing pulmonary vasculature, reducing PASMC proliferation, and restoring endothelial function.^455,461^ A study found that the RhoA/ROCK pathway mediates 8-isoprostane-induced ET-1 production in PASMCs, which may help explain the therapeutic effects of antioxidants and ROCK inhibitors in chronic PH.^462^ Despite these promising findings, the clinical use of ROCK inhibitors is limited by potential systemic side effects, such as hypotension.^463^ Therefore, research efforts are ongoing to develop more selective inhibitors and strategies that target the RhoA/ROCK pathway specifically in the pulmonary circulation.

#### Targeting PPAR signaling pathways

Pharmacological activation of p53 and PPARγ-p53 facilitates DNA repair in PAECs from PAH patients with *BMPR2* mutations without inducing apoptosis.^464^ In apoE-deficient mice with insulin resistance, PPARγ activation by rosiglitazone reversed PAH, RVH, and abnormal pulmonary artery muscularization.^465^ Numerous studies have shown that PPARγ activation reduces PASMC proliferation, inflammation, endothelial dysfunction, vasoconstriction, and vascular remodeling in PH.^158,308,466–469^ The pharmacological effect of PPARγ activation in attenuating various forms of PH involves modulating 5-HT-induced AP-1 activity,^470^ HO-1 and p21 (WAF1),^471^ store-operated Ca^2+^ entry, TRPC and HIF-1α,^130,472–475^ ERK1/2-NF-κB-NOX4-H_2_O_2_ signaling axis,^158,476^ miRNAs,^477,478^ and HUWE1/miR-98^479^ pathways.

In CTEPH, PPARα agonists effectively reduce TAFIα, which contributes to thrombus formation, vascular remodeling, and inflammation.^480^ In MCT-PAH, PPARα activation by fenofibrate alleviates oxidative stress and inflammation, downregulates NOX-1 expression, and preserves RV function.^481^

Advancing our understanding of selective modulators could enable the development of PPARβ/δ-based therapies that target PAH and right heart dysfunction while minimizing adverse effects, offering a balanced and innovative therapeutic strategy for PH.

#### Targeting ER signaling pathways

Therapeutically, targeting these distinct ER pathways offers significant opportunities for PH management. Selective ER modulators that activate ERα or ERβ in a tissue-specific manner could enhance pulmonary vascular function and RV adaptation without causing systemic estrogenic side effects.^315,482^ GPER1 agonists, such as G1, show particular promise for ameliorating PH-related dysfunction in postmenopausal women and men while maintaining a favorable safety profile, as evidenced in pre-clinical studies.^311^ Combining receptor-specific therapies with complementary strategies, such as enhancing BMPR2 signaling or inhibiting pro-fibrotic pathways like TGF-β, may optimize outcomes in PH. These approaches target the complex interplay between inflammation, vascular remodeling, and RV dysfunction, offering a personalized therapeutic framework to address the sex-specific and systemic challenges of PH.

#### Targeting JAK/STAT signaling pathway

From a therapeutic perspective, targeting JAK/STAT signaling presents a promising approach for PH management. Inhibition of the JAK/STAT pathway, particularly through JAK1/2 inhibitors like ruxolitinib, offers a promising therapeutic strategy in PAH by reducing vascular remodeling, counteracting CAV1 deficiency-induced endothelial dysfunction, and limiting aberrant STAT3-driven endothelial proliferation in IPAH.^27^^,^^317^^,^^319^

#### Targeting CaSR signaling pathway

Dihydropyridine Ca²⁺ channel blockers, such as nifedipine, exacerbate mPAP in IPAH patients by potentiating CaSR activity in PASMCs, independent of their effects on Ca²⁺ channels.^483^ This suggests that targeting CaSR could potentially provide a more effective therapeutic strategy by avoiding the adverse effects of these blockers in IPAH patients, who often have upregulated CaSR activity. Combining statins with methyl-allylthiosulfinate modulate GGPP levels to provide therapeutic benefits for hypoxia-PH by regulating RhoA/ROCK signaling, Rab10-mediated trafficking of CaSR to the cell membrane, hypoxia-induced CaSR over-expression, and HIMF binding to CaSR.^207,210^ In PH models, CaSR targeting with calcilytics like NPS 2143 reduces RV pressure, hypertrophy, and fibrosis, suggesting a promising therapeutic approach. In IPAH patients, calcilytics inhibit excessive PASMC proliferation, highlighting the potential of CaSR blockade in reducing vascular remodeling.^320,484,485^ MiRNAs such as miR-16, miR-429, and miR-424-5p regulate CaSR expression, reducing Ca²⁺ influx and inhibiting PASMC proliferation, offering a potential therapeutic strategy for PH.^486,487^ Compounds like tetramethylpyrazine, sulfur dioxide, and chloroquine downregulate CaSR expression in PASMCs under hypoxia, supporting the therapeutic potential of targeting CaSR in PH,.^488^ Combination therapies with calcilytics and PDE5 inhibitors, like sildenafil, enhance anti-proliferative effects in IPAH-PASMCs, suggesting CaSR targeting could boost current IPAH treatments.^489^

#### Targeting Hippo signaling pathway

Therapeutic strategies targeting Hippo signaling components hold significant potential in managing PH. ILK inhibition with Cpd22 restores LATS1 activity, suppresses YAP, and reduces vascular remodeling in experimental PH models.^326^ Similarly, ACE2 activation upregulates LATS1 and induces apoptosis through Hippo/YAP signaling, ameliorating pulmonary arterial remodeling, with effects mitigated by ACE2 inhibitors.^490^ Siah2 inhibition using compounds like Vitamin K3 or MG-132 effectively restores LATS1/2 levels, attenuates YAP activation, and decreases PAVSMC proliferation and remodeling.^327^ Other approaches include AMPK activation and luteolin, which suppress YAP via the Hippo/PI3K/Akt pathway,^251,422^ and blocking upstream signaling such as the S1P/S1PR/STAT3/miR-135b/YAP/Notch3 axis, which mitigates PAVSMC hyper-proliferation.^491^ Noncanonical pathways involving BUB3 and FOXO or regulating SIK1 may also provide novel therapeutic targets.^328^ These findings underscore the therapeutic promise of targeting Hippo/YAP signaling to reverse pulmonary vascular remodeling in PH.

#### Targeting Nrf2/HO-1 signaling pathway

Activation of Nrf2/HO-1 offers a protective mechanism by reducing oxidative stress and mediating iron detoxification, highlighting its potential as a therapeutic target in PH management, as supported by recent pre-clinical studies.^330,492,493^ Dysregulated iron can exacerbate oxidative damage via the Fenton reaction, contributing to inflammation, VSMC proliferation, and endothelial injury,^494,495^ hallmarks of PH. Nrf2-induced HO-1 expression may counter these effects by facilitating controlled iron release and sequestration, while carbon monoxide and biliverdin exert anti-inflammatory and vasoprotective effects.^496,497^ This dual regulatory role positions the Nrf2/HO-1 pathway as a promising target for innovative PH therapies aimed at mitigating iron-driven oxidative stress and vascular remodeling. Research into this pathway may uncover novel strategies for addressing the iron dysregulation underlying PH progression.

#### Targeting PARP1/PKM2 signaling pathway

In a study, CMs-specific deletion of PARP1 in Su/Hx-PAH mice and pharmacologic inhibition of PARP1 with olaparib or enforced PKM2 tetramerization with TEPP-46 in Prealbumin (PAB)-induced rats improved RV function, reduced fibrosis, inflammation, oxidative stress, and maladaptive remodeling by reversing glycolytic shifts and modulating metabolic and inflammatory pathways.^343^ Thus, targeting this axis therapeutically could restore metabolic balance and mitigate vascular remodeling in PAH. Approaches such as PARP inhibitors or modulators of PKM2 activity offer potential to slow disease progression and support RV function during PH.

#### Targeting cGAS-STING signaling pathway

Recent findings demonstrate that pharmacological agents, including CGRP and β-sitosterol (SITO), exhibit protective effects against PH by modulating the cGAS-STING axis. CGRP, induced by cinnamaldehyde, protects mitochondria and inhibits the cGAS-STING-NF-κB pathway via PKA, thereby alleviating vascular remodeling and inflammatory responses.^348^ Similarly, SITO prevents PASMC hyper-proliferation, promotes apoptosis, and mitigates phenotypic switching by suppressing DNA damage and cGAS-STING pathway activation.^349^ In vivo models of PH show that both CGRP and SITO effectively reverse MCT-induced pulmonary vascular remodeling and improve RV function.^348,349^ However, while these findings highlight the therapeutic promise of targeting the cGAS-STING axis, more research is needed to fully elucidate the underlying mechanisms, optimize dosing strategies, and evaluate long-term efficacy and safety in preclinical and clinical settings.

### Clinical implications, challenges, and side effects: insights based on targeting crosstalks

PAH (Group 1) has long been a key focus of drug development, as outlined in clinical practice guidelines. Currently, treatment options commonly used in clinical practice include calcium channel blockers, PGI analogs, and PGI receptor agonists targeting the PGI pathway; PDE5 inhibitors and Sgc stimulators targeting the NO pathway; and ET-1 receptor antagonists targeting the ET-1 pathway.^1,2,6^ Despite these available treatments, not all patients respond effectively. This limitation arises from the complexity of the involved cells and signaling pathways, with many of the underlying mechanisms still not fully understood.

According to the literature, more targeted approaches, such as small molecule agonists or gene therapies aimed at enhancing BMPR2 signaling, could be explored to treat the underlying pathophysiology of PH with abnormal BMPR2 signaling.^21,386,498^ Improving the balance between TGF-β and BMPR2 signaling may also offer a viable therapeutic strategy, with the potential to reduce fibrosis and vascular remodeling in PH.^499^ Moreover, the promising results from Phase III clinical trials on Sotatercept targeting the TGF-β pathway reinforce this approach, highlighting a compelling path forward for innovative treatments. TGF-β inhibitors, such as pirfenidone or losartan, which are already in clinical use for conditions like IPF,^500^ could be repurposed to manage PH by mitigating EndMT and the inflammatory processes contributing to vascular remodeling. However, systemic inhibition of TGF-β signaling could potentially impair wound healing, increase susceptibility to infections, or exacerbate fibrosis in other tissues.^20,501,502^ In particular, Notch inhibitors, such as γ-secretase inhibitors, hold promise in modulating SMC proliferation and migration, but their use might lead to adverse effects like increased susceptibility to cancer, developmental defects, cognitive decline, or altered immune function.^503,504^ Furthermore, by enhancing AMPK signaling, we may be able to reduce PASMC proliferation, inhibit EndMT, and prevent excessive fibrosis. However, the activation of AMPK could also have unintended effects, such as compromising normal cellular functions related to autophagy or metabolic regulation, potentially leading to metabolic imbalances or impaired tissue repair in certain contexts.^505,506^

Additionally, by unraveling the intricate interactions among these signaling pathways, we gain deeper insight into the mechanisms driving pulmonary vascular remodeling and dysfunction. Targeting key downstream pathways such as TGF-β, PI3K/Akt, NF-κB, and MAPKs opens a dynamic avenue for mitigating vascular remodeling by reversing endothelial dysfunction in PAECs, tackling excessive proliferation in PASMCs, inhibiting ECM remodeling in PAFs as thoroughly reviewed. The complexity of PH determines that single-pathway targeted therapy is difficult to cope with. Multi-pathway targeted therapy strategies can simultaneously regulate multiple pathological processes, thereby improving treatment efficacy, reducing disease progression, and increasing long-term survival rates. For example, the AMBITION trial showed that combining ET-1 receptor antagonists with PDE5 inhibitors is more effective than monotherapy.^507–512^ This validates the effectiveness of multi-channel regulatory strategies in the treatment of PH. Additionally, combination therapies involving calcilytics and other pharmacological agents such as PDE5 inhibitors, including sildenafil, have shown enhanced anti-proliferative effects in IPAH-PASMCs, suggesting that targeting CaSR could improve the efficacy of current treatments for IPAH.^489^ Combining these approaches in multi-target therapies could enhance treatment efficacy but also necessitate careful monitoring of adverse interactions between pathways. Side effects such as organ toxicity, immunosuppression, dysregulated cellular differentiation, or other adverse events may arise from the non-specific effects of these interventions, particularly at inappropriate dosages. This underscores the importance of developing personalized treatment strategies that account for patient-specific variables, including individual sensitivities, molecular profiles, and optimal therapeutic dosages to minimize risks and maximize efficacy.

Incorporating the findings from these molecular insights into existing treatment regimens may yield more personalized and effective therapies for PH. Combining current therapies like PDE5 inhibitors with BMPR2 pathway enhancers, AMPK activators, or TGF-β modulators could improve overall treatment outcomes by directly addressing the underlying molecular mechanisms driving the disease. For instance, combining TGF-β inhibitors with drugs like PDE5 inhibitors may increase the risk of systemic hypotension, while simultaneous inhibition of multiple pathways, such as TGF-β, PI3K/Akt, and Notch, could lead to cardiac or liver toxicity. More research is needed to optimize these combinations, minimize potential risks, and ensure their safety and efficacy in clinical settings.

## Conclusion and perspectives

PH remains a complex and challenging condition with significant morbidity and mortality. Current therapeutic approaches primarily target the PGI, NO, and ET-1 pathways; However, research indicates that, beyond these conventional pathways, several abnormal signaling pathways play crucial roles in the pathogenesis of PH. Through a comprehensive analysis of the existing literature, fourteen signaling pathways (BMPR2, TGF-β, HIF, MAPK, NF-κB, NLRP3, Notch, Wnt, RhoA/ROCK, JAK/STAT, CasR, Hippo, PARP1/PKM2, cGAS-STING) have been identified to exhibit consistent activation patterns across different groups of PH. These pathways contribute to pulmonary vascular remodeling or RVH by regulating key processes, including inflammation, anti-apoptosis, proliferation, ECM remodeling, EndMT, fibrosis, and mitochondrial metabolic reprogramming. In different types of PH or at various stages within the same type, some pathways exhibit opposing roles. For instance, in PAECs from patients with PAH, the PI3K/Akt pathway is typically suppressed, leading to inflammation, apoptosis, and endothelial damage. However, in PAECs associated with MCT-PAH, PAH due to congenital heart disease, and hypoxia-PH, the activation of the PI3K/Akt pathway promotes excessive cell proliferation. This discrepancy aligns with the dynamic nature of PAH progression, where the early stages are dominated by cell apoptosis and injury, while later stages shift towards excessive cell proliferation. It is noteworthy that a pivotal hub molecule, Akt, has emerged within the network of crosstalk among signaling pathways. Therefore, we speculate that targeting Akt may hold greater promise for the treatment of various groups of PH.

Moreover, the same signaling pathway can have contrasting effects depending on the cell type. For example, NF-κB activation promotes apoptosis in PAECs but enhances anti-apoptotic capabilities in PASMCs and PAFs. The FGF2/FGFR pathway shows dual roles, with FGFR activation in PASMCs promoting proliferation and remodeling, while inhibition in PAECs prevents EndMT and vascular remodeling. These cell-type-specific differences further complicate the pathology of PH, highlighting the importance of considering specific signaling pathways’ roles across different cell types and disease stages when developing treatment strategies. Although the role of PAECs and PASMCs in the pathogenesis of PH has been the focus of predominant research, the contributions of PAFs, pericytes, and immune cells are also garnering increasing attention. However, further in-depth investigation is warranted to elucidate their specific contributions to PH.

Notably, the AMPK signaling pathway appears to have a protective role in PH, as it shows significant inhibition across various cell types in multiple PH models. Even though AMPK is activated by short-term hypoxic stimulation in vitro, it is speculated that it should be a protective defense mechanism of the body. Consequently, activating this pathway with AMPK agonists could offer a promising new therapeutic approach for PH management. In preclinical trials, Metformin as an anagogic of AMPK showed beneficial effects on Group 1 and 3 of PH, and the combination of it and other drug treatments also improved Group 2 and 5 of PH. Moreover, PPARγ inhibition may contribute to cellular processes that drive inflammation, vascular remodeling, and vasoconstriction at the tissue level. Estrogen signaling, via GPER1 activation, mitigates inflammation and remodeling, while ERα and ERβ inhibition limits proliferation and ECM remodeling. Nrf2/HO-1 inhibition exacerbates oxidative stress and iron dysregulation, leading to iron deficiency in PH. Therapeutic strategies targeting PPARγ and GPER1 have demonstrated benefits in animal models. Given that some signaling pathways have only recently been implicated in the pathogenesis and progression of PH, further research is warranted to elucidate their feasibility as targets for therapeutic intervention.

Additionally, some drugs can target multiple signaling pathways to treat multiple groups of PH, such as luteolin, Resveratrol, and Dioscin. The use of these drugs alone or in combination with other drugs may improve the effectiveness of treatment. Although multi-target therapy has broad prospects, it faces challenges such as drug interactions, high costs, and individual differences. Future research directions should focus on optimizing combination therapies through biomarkers and molecular diagnostics to achieve precision and personalized medicine. Future treatment options also include clustered regularly interspaced short palindromic repeats gene editing and stem cell therapy aimed at restoring pathway function caused by gene mutations such as *BMPR2*. The treatment strategy for PH is shifting from targeting a single pathway to addressing multiple pathways, and the successful implementation of this approach holds the potential to transform PH into a manageable chronic disease and may lead to a cure in the future.
