# Correction: Signaling pathways and targeted therapy for pulmonary hypertension

**DOI:** 10.1038/s41392-025-02564-6

**Published:** 2026-01-09

**Authors:** Joseph Adu-Amankwaah, Yue Shi, Hequn Song, Yixuan Ma, Jia Liu, Hao Wang, Jinxiang Yuan, Kun Sun, Qinghua Hu, Rubin Tan

**Affiliations:** 1 Department of Physiology, School of Basic Medical Sciences, Xuzhou Medical University, Xuzhou, Jiangsu China; 2 First Clinical Medical School, Xuzhou Medical University, Xuzhou, Jiangsu China; 3 Second Clinical Medical School, Xuzhou Medical University, Xuzhou, Jiangsu China; 4 Lin He’s Academician Workstation of New Medicine and Clinical Translation, Jining Medical University, Jining, Shandong China; 5 Institute for Developmental and Regenerative Cardiovascular Medicine, Xinhua Hospital, School of Medicine, Shanghai Jiao Tong University, Shanghai, China; 6 Department of Pathophysiology, School of Basic Medicine, Tongji Medical College, Huazhong University of Science and Technology (HUST), Wuhan, China; 7 Key Laboratory of Pulmonary Diseases of Ministry of Health, Tongji Medical College, HUST, Wuhan, China

Correction to: *Signal Transduction and Targeted Therapy* 10.1038/s41392-025-02287-8, published online 01 July 2025

In this article the e-mail address of the corresponding author Rubin Tan was incorrectly given as tanrubin@126.com where it should have been tanrubin11@126.com.

